# Regulation of gene expression is associated with tolerance of the Arctic copepod *Calanus glacialis* to CO_2_‐acidified sea water

**DOI:** 10.1002/ece3.3063

**Published:** 2017-08-02

**Authors:** Allison Bailey, Pierre De Wit, Peter Thor, Howard I. Browman, Reidun Bjelland, Steven Shema, David M. Fields, Jeffrey A. Runge, Cameron Thompson, Haakon Hop

**Affiliations:** ^1^ Norwegian Polar Institute Tromsø Norway; ^2^ Department of Arctic and Marine Biology Faculty of Biosciences Fisheries and Economics UiT The Arctic University of Norway Tromsø Norway; ^3^ University of Gothenburg Department of Marine Sciences Sven Lovén Centre for Marine Sciences Tjärnö Sweden; ^4^ Austevoll Research Station Institute of Marine Research Storebø Norway; ^5^ Bigelow Laboratory for Ocean Sciences East Boothbay ME USA; ^6^ Gulf of Maine Research Institute University of Maine Orono ME USA

**Keywords:** ocean acidification, pH, phenotypic buffering, RNA‐seq, stress response, transcriptomics

## Abstract

Ocean acidification is the increase in seawater *p*CO_2_ due to the uptake of atmospheric anthropogenic CO_2_, with the largest changes predicted to occur in the Arctic seas. For some marine organisms, this change in *p*CO_2_, and associated decrease in pH, represents a climate change‐related stressor. In this study, we investigated the gene expression patterns of nauplii of the Arctic copepod *Calanus glacialis* cultured at low pH levels. We have previously shown that organismal‐level performance (development, growth, respiration) of *C. glacialis* nauplii is unaffected by low pH. Here, we investigated the molecular‐level response to lowered pH in order to elucidate the physiological processes involved in this tolerance. Nauplii from wild‐caught *C. glacialis* were cultured at four pH levels (8.05, 7.9, 7.7, 7.5). At stage N6, mRNA was extracted and sequenced using RNA‐seq. The physiological functionality of the proteins identified was categorized using Gene Ontology and KEGG pathways. We found that the expression of 151 contigs varied significantly with pH on a continuous scale (93% downregulated with decreasing pH). Gene set enrichment analysis revealed that, of the processes downregulated, many were components of the universal cellular stress response, including DNA repair, redox regulation, protein folding, and proteolysis. Sodium:proton antiporters were among the processes significantly upregulated, indicating that these ion pumps were involved in maintaining cellular pH homeostasis. *C. glacialis* significantly alters its gene expression at low pH, although they maintain normal larval development. Understanding what confers tolerance to some species will support our ability to predict the effects of future ocean acidification on marine organisms.

## 1 INTRODUCTION

Anthropogenic CO_2_ emissions are increasing the *p*CO_2_ of the atmosphere and the oceans (Le Quéré, Raupach, & Canadell, 2009). Increased *p*CO_2_ in surface waters alters the carbonate chemistry of sea water, ultimately increasing hydrogen ion (H^+^), bicarbonate ion (HCO_3_^−^), and dissolved inorganic carbon (DIC) concentrations and decreasing pH and carbonate ion (CO_3_^2−^) concentrations. This process is termed ocean acidification (Caldeira & Wickett, 2003; Orr, Fabry, & Aumont, 2005). While occurring in all of the world's oceans, the largest changes in carbonate chemistry are expected to occur in the Arctic seas (Steinacher et al., 2009; Bellerby, Anderson, & Azetsu‐Scott, 2013). Low‐salinity Arctic waters have low buffering capability, resulting in lower pHs for a given amount of CO_2_, and projections indicate further freshening from ice melt and river input (Steinacher et al., 2009; Bellerby et al., 2013). The reduction in sea ice with climate change may also increase the atmosphere‐ocean flux of CO_2_ (Bates, Moran, Hansell, & Mathis, 2006; Arrigo, Pabi, Van Dijken, & Maslowski, 2010; Parmentier et al., 2013; Barber, Hop, & Mundy, 2015).

High *p*CO_2_, and associated low pH, is a stressor for some marine organisms (Kroeker, Kordas, Crim, & Singh, 2010; Wittmann & Pörtner, 2013). Many physiological processes including gas exchange, ion transport, enzyme activity, and protein function rely on a specific range of intracellular pH (Whiteley, 2011). Maintaining pH homeostasis can incur additional energetic costs when organisms must actively compensate for low extracellular pH using energy‐expensive ion pumps (Pörtner, 2008; Whiteley, 2011), leaving less energy for growth, development, and reproduction (e.g., Stumpp, Wren, Melzner, Thorndyke, & Dupont, 2011).

In calanoid copepods, exposure to pH levels projected for the next century has produced inconsistent effects on organismal‐level measures of fitness and performance. Some species show no changes in development, respiration, and feeding rates with lowered pH (Kurihara & Ishimatsu, 2008; Mayor, Everett, & Cook, 2012; McConville et al., 2013; Runge, Fields, & Thompson, 2016), whereas other calanoid species are detrimentally affected (Zhang, Li, Wang, & Guo, 2011; Fitzer et al., 2012; Pedersen, Håkedal, & Salaberria, 2014; Thor & Dupont, 2015). Effects may vary by species, but also by life stage, with eggs and early naupliar stages being most vulnerable (Cripps, Lindeque, & Flynn, 2014; Pedersen, Våge, Olsen, Hammer, & Altin, 2014).

The ability of an organism to maintain fitness‐related traits, like growth, reproduction, and development, while exposed to a stressful environment by adjusting underlying physiological processes has been referred to as phenotypic buffering (Reusch, 2014). Phenotypic buffering is crucial for tolerance of spatially and temporally variable environments, and organisms that are capable of phenotypic buffering may be able to adapt more easily to long‐term environmental change (Donelson, Munday, McCormick, & Pitcher, 2012; Miller, Watson, Donelson, McCormick, & Munday, 2012). However, depending on the mechanism, long‐term utilization of phenotypic buffering in a stressful environment may also incur an energetic cost. Phenotypic buffering may involve, among other things, altered gene expression, compensatory feeding, and changes in energy allocation (Sunday et al., 2014).

Recent progress in high‐throughput genomic technology has made possible the sequencing of entire transcriptomes (RNA‐seq), with the potential to measure expression in all transcribed genes, not limited to a targeted set of genes in a microarray. This has allowed for a universal, nontargeted exploration of gene expression (or transcriptomic) responses to environmental drivers. Investigating the transcriptomic response of species to climate change‐related environmental drivers has the potential to provide greater understanding of why and how some species are more tolerant to change than others (Franssen, Gu, & Bergmann, 2011; Reusch, 2014; Seneca & Palumbi, 2015).

The calanoid copepod *Calanus glacialis* is a key component of the Arctic marine ecosystem, comprising up to 90% of the zooplankton biomass in shelf regions (Conover & Huntley, 1991; Blachowiak‐Samolyk et al., 2008). It is an important prey for fish, seabirds, and baleen whales (Lowry, 1993; Karnovsky, Kwasniewski, Weslawski, Walkusz, & Beszczynska‐Möller, 2003; Hop & Gjøsæter, 2013). *Calanus glacialis* maintains its vital rates at normal levels when exposed to low pH. Organismal‐level measures such as respiration, ingestion, survival, gonad maturation, egg production, and naupliar growth and development are unaffected by low pH (Weydmann, Søreide, Kwasniewski, & Widdicombe, 2012; Hildebrandt, Niehoff, & Sartoris, 2014; Hildebrandt, Sartoris, Schulz, Riebesell, & Niehoff, 2015; Bailey, Thor, & Browman, 2016). In this study, we sought to characterize the molecular‐level response of *C. glacialis* nauplii to lowered pH to elucidate the physiological processes involved in this tolerance. Understanding the mechanisms of phenotypic buffering that allow some species to tolerate a wide range of pH is important for the field of ocean acidification research. Not only can it help detect low‐level stress potentially unnoticed in organismal‐level measurements, but it also contributes to understanding how some species, but not others, can tolerate low pH. We quantified whole transcriptome gene expression in the last naupliar stage (N6) of *C. glacialis* which had been exposed to lowered pH as they developed from eggs (over a month). We hypothesized that gene expression would respond to the pH treatment despite the lack of change in developmental rate, growth, and respiration observed during the same experiment (Bailey et al., 2016) and that these modulations would be related to the maintenance of growth and development. Furthermore, we expected that the physiological functions affected by pH, as indicated by altered expression of genes coding for functional groups of proteins, would be similar to those affected in other marine organisms at low pH and would, therefore, contribute to an understanding of a generalized molecular response to low pH.

## 2 METHODS

### 2.1 Collection and exposure

The collection and culturing of the copepods are detailed in Bailey et al. (2016). Briefly, *C. glacialis* were collected in Rijpfjorden, northeast Svalbard (80°27′54″N, 021°56′63″E) in January 2013 (−1 to 0°C) and held for 50 days prior to the experiment in 40 L flow‐through tanks in a cooling container at 2°C. Once females were mature, a pool of 1,900 females was used to inoculate 12 tanks (3 replicate tanks for each of four pCO2 treatments) of 40 L with eggs (7,039 ± 1,178 SE eggs per tank) in turn over 19 days. Females laid eggs in mesh‐bottomed buckets suspended in the tanks, which allowed eggs to settle into the tank but retained females. During inoculations, the tanks were filled with ambient sea water; the pH treatment began after the females were removed, with tanks reaching target pH in about 4 hr.

The eggs developed through six naupliar stages to the final naupliar stage, N6. Development was tracked every second day by collecting 30 individuals from each tank and identifying naupliar stages under a stereoscope. As stages N1 and N2 do not feed, the nauplii were fed with a mix of live algae mixture starting at stage N3 (*Isochrysis* sp. [CCAP 927/14]*, Rhodomonas baltica* [NIVA 5/91], *Chaetocerous muelleri* [CCAP 1010/3], and *Skeletonema costatum* [NIVA BAC‐1]) at satiating concentrations (>200 μgC/L, Campbell, Wagner, Teegarden, Boudreau, & Durbin, 2001).

### 2.2 Treatment water

Projections of Arctic ocean acidification for 2,100 are ΔpH = −0.45 following the Intergovernmental Panel on Climate Change's (IPCC) RCP8.5/SRES A2 carbon emission scenario (Steinacher et al., 2009; Ciais, Sabine, & Bala, 2013), and ΔpH = ‐0.7 by 2,300 following the extended IPCC ERC8.5 scenario (Caldeira & Wickett, 2003; Collins, Knutti, & Arblaster, 2013). Accordingly, four pH treatments were chosen to simulate the current conditions at the collection site (high pH; pH 8.05; 320 μatm pCO_2_), ambient water at the research station (ambient; pH 7.90; 530 μatm), and projected conditions in Arctic seas in years 2100 (mid‐pH; pH 7.70; 800 μatm) and 2300 (low‐ pH; pH 7.50; 1,700 μatm).

For each treatment, pH was controlled in a 100 L mixing tank by a pH controller (Endress and Hauser, Liquiline CM 442) connected to a pH electrode (Endress and Hauser Orbisint CPS11D glass electrode) calibrated with NIST pH standards (pH_NBS_). The controller maintained the target pH by regulating the addition of either CO_2_‐enriched sea water (for the low‐ and mid‐pH treatments) or CO_2_‐stripped air (for the high‐pH treatment) to ambient sea water in the mixing tanks. The CO_2_‐enriched sea water (pH ~5.5) was created by bubbling pure CO_2_ continuously into ambient sea water. CO_2_‐stripped air was produced by forcing air through a CAS series CO_2_ adsorber (Twin Tower Engineering). Sea water from each of the four mixing tanks was pumped into header tanks, each of which fed three 40‐L experimental tanks. Inflow of treatment water to the experimental tanks was 10–20 L/min.

The pH_NBS_ of each treatment was logged continuously by the pH electrode in each mixing tank. Additionally, water samples (100 mL) from each experimental tank were taken every few days (*n* = 10 during the experiment) for spectrophotometric measurement of total scale pH (pH_T_) with the pH‐sensitive indicator dye m‐cresol purple (MCP) (Dickson, Sabine, & Christian, 2007). Carbonate chemistry and nutrient concentrations were analyzed collected weekly intervals (*n* = 8) from one experimental tank of each treatment and preserved with a saturated mercuric chloride solution (Riebesell, Fabry, Hansson, & Gattuso, 2010). Temperature was measured daily in every tank. Methods of spectrophotometric pH_T_ and carbonate chemistry measurements are described in more detail in Bailey et al. (2016). Carbonate chemistry parameters in the experiment are given in Table S9.

### 2.3 Nauplii sampling

Nauplii were collected from tanks when development had reached stage N6, 35–38 days post‐egg laying. The sample was gently sieved and transferred to a petri dish with sea water, where the nauplii were identified to stage by stereoscope. Stage N6 nauplii were transferred to a 10‐mL Falcon tube, and RNA*later* (Qiagen) was added immediately to preserve the RNA from degradation; the time from collection to preservation was <10 min. Two pools of 10 individuals were collected from each tank. With three replicate tanks per pH, this resulted in six samples from each pH treatment (with five in the pH 7.95 treatment due to sample loss, 23 samples total). The samples were then stored at −80°C for 9 months until extraction.

### 2.4 RNA‐seq

Total RNA was extracted from each of the 23 samples using an RNeasy Mini kit (Qiagen). RNA concentration was measured with a high‐sensitivity QuBit fluorometric assay (Life Technologies), and its quality was tested with a denaturing MOPS gel electrophoresis assay. From total RNA, mRNA was isolated and fragmented, and the complementary DNA (cDNA) libraries were synthesized according to the TruSeq RNA sample preparation v2 low sample protocol (Illumina). Concentrations of cDNA were measured with the high‐sensitivity QuBit fluorometric assay. Barcoded index adapters were ligated, and samples were pooled equimolarly (5–6 samples per pool) and sequenced single‐end, with a 50 bp read length in four lanes in an Illumina HiSeq 2500 high‐throughput sequencing machine (High Output mode) at the Swedish National Genomics Infrastructure (SNP & SEQ platform) in Uppsala, Sweden. All raw data were submitted to National Center for Biotechnology Information's (NCBI) Short Read Archive (SRA, accession numbers SAMN05990738‐SAMN05990760; BioProject ID: PRJNA352656).

Sequenced reads were quality‐checked and used for *de novo* transcriptome assembly and quantification of gene expression following the pipeline in De Wit, Pespeni, and Ladner (2012) and the associated open‐access scripts (https://github.com/DeWitP/SFG; downloaded on 13 January 2016). In short, low‐quality sequenced bases (Phred score < 20) were removed using the Fastx‐toolkit (0.0.13), as were adapter sequences. Duplicate reads were identified and counted using MarkDuplicates (Picard tools 2.0.1).

A *de novo* transcriptome was compiled from 636,738,663 reads into contiguous sequences (contigs, putative transcripts) using CLC Genomics Workbench (Ver. 8.5.1; QIAGEN Aarhus A/S). The CLC De Novo Assembly tool was used with simultaneous mapping (minimum contig length = 200 bp, mismatch cost = 1, insertion cost = 2, deletion cost = 2, no global alignment, update contigs based on mapping) and default settings (length fraction = 0.5, similarity fraction = 0.8). Transcriptome assembly and annotation completeness were evaluated using Benchmarking Universal Single‐Copy Orthologs (BUSCO) analysis, which is based on evolutionarily informed expectations of gene content (ver 2.0; Simão, Waterhouse, Ioannidis, Kriventseva, & Zdobnov, 2015). Individual reads were mapped to this transcriptome using the Burrows‐Wheeler Aligner (0.7.12) with the following settings: maxDiff = 0.01, maxSeedDiff = 5, seedLen = 30, and nThrds = 2. As a proxy of gene expression, the counts of uniquely mapped reads mapping to each contig were compiled for each sample using a custom script from De Wit et al. (2012) with a mapping quality threshold of 20 and a read length threshold of 20 bp.

The proportion of duplicate reads was high. Removal of 110 contigs which matched known *C. glacialis* ribosomal sequences (1,006 sequences, downloaded from NCBI on 12 April 2016) reduced the number of duplicates, resulting in 45%–93% duplicate reads per sample. As duplicate reads may arise from highly expressed genes and may have biological significance, they were not removed (Parekh, Ziegenhain, Vieth, Enard, & Hellmann, 2015).

To associate physiological function to each of the contigs, the proteins for which the contigs coded were identified by searching the arthropod sequences in NCBI's nonredundant (nr) protein database and the entire UniProt protein database (downloaded on July 6 2016) (e‐value cutoff = 1 × 10^−5^; Blastx 2.3.0+). Hypothetical or predicted protein annotations in nr were excluded by discarding matches with the following words: “hypothetical,” “predicted,” “unknown,” and “putative.” The sequences corresponding to known proteins were annotated with biological process and molecular function Gene Ontologies (GO), a classification of physiological function spanning from very broad to specific descriptions for each protein, as well as Kyoto Encylopedia of Genes and Genomes (KEGG) Gene ID using their Uniprot ID and the Uniprot flatfile database (uniprot.org, downloaded on 13 July 2016, http://www.genome.jp/kegg). For each contig with KEGG Gene ID, the KEGG orthologs and their associated KEGG pathways were downloaded from the KEGG REST API using the R package KEGGREST (Tenenbaum, 2016). KEGG orthologs, and the KEGG pathways they are associated with, are not species specific and are thus designed for cross‐species comparisons, allowing their application in nonmodel species like *C. glacialis*.

### 2.5 Gene expression analyses

Differentially expressed contigs (DECs), those whose expression varied significantly with pH (treated as a continuous variable), were identified with the R package DESeq2 (version 1.10.1, Love, Huber, & Anders, 2014a). DESeq2 outperforms nearly all other HTS gene expression R packages in detecting significantly differently expressed genes (Seyednasrollah, Laiho, & Elo, 2013; Love, Huber, & Anders, 2014b; Schurch, Schofield, & Gierliński, 2016), taking into account common sources of error such as low sample size, low‐count genes and high variability using shrinkage estimators of dispersion and fold change (Love et al., 2014a). Contig expression is discussed here as gene expression.

To determine whether gene expression varied significantly along a continuous pH scale, DESeq2 fits generalized linear models (GLMs) to raw counts of each gene, with a logarithmic link (log2) and negative binomial distribution using a shrunken dispersion estimate (Love et al., 2014a). The GLM fit produces the overall expression strength of the gene and the log fold change (LFC) per unit of pH. LFCs are then shrunk to reduce heteroscedasticity caused by low‐count genes showing high LFCs, with low‐count, low sample size, or high‐dispersion genes shrunk the most. Ward tests with Benjamini‐Hochberg procedure are used to adjust for multiple testing in DESeq2. Significant differently expressed contigs were defined as those with |LFC| > 1 and adjusted *p*‐value (false discovery rate, FDR) <0.1.

To visualize the similarity of expression of the DECs across samples, a heatmap was created, with the raw contig counts transformed using the rlog function in DESeq2 and plotted using “aheatmap” in the R package NMF, which centers and scales expression of each contig (0.17.6, Gaujoux & Seoighe, 2010). DESeq2 and heatmap were run on R version 3.2.3 (R Core Team, 2015) and the graphics package ggplot2, version 2.0.0 (Wickham, 2009).

### 2.6 Gene set enrichment

FatiScan of the Babelomics5 suite was used to detect functional sets of contigs significantly affected by pH (Al‐Shahrour et al., 2007; Montaner & Dopazo, 2010; Alonso, Salavert, & Garcia‐Garcia, 2015). FatiScan is a gene set enrichment analysis (GSEA) tool sensu Khatri, Sirota, and Butte (2012). The first‐generation over‐representation tools searched for enriched pathways in a subset of genes selected by a threshold of fold change or *p*‐value. However, focusing only on a subset of high‐fold‐change genes can introduce bias (Dalman, Deeter, Nimishakavi, & Duan, 2012) and distracts from detecting genes that truly affect fitness, which often show mild fold changes (Evans, 2015). In contrast, second‐generation GSEA tools use a threshold‐free method that utilizes all genes and their fold changes. They can, therefore, more sensitively detect functional sets of genes (genes in the same GO or pathway) that behave in moderate, but coordinated, manner in response to treatment. Enrichment of GO and KEGG pathways in genes up‐ and downregulated with pH was analyzed by the FatiScan logistic model using the log fold changes of all genes, with our *de novo* transcriptome as a reference, and a significance cutoff (α) of FDR‐adjusted *p*‐value of .05. GOs with log‐odds ratio (LOR) < 0 were taken to be downregulated, and LOR > 0 upregulated.

To distill the most important functions affected by pH, the significant GOs were clustered based on their semantic similarity and GSEA *p*‐value (SimRel > 0.5; Schlicker, Domingues, Rahnenführer, & Lengauer, 2006) using ReViGO (Supek, Bošnjak, Škunca, & Šmuc, 2011). These results were represented in a network using the network‐plotting tool Cytoscape (version 3.4.0, Shannon, Markiel, & Ozier, 2003) with the Prefuse Force‐directed Layout based on SimRel weight between GO nodes.

## 3 RESULTS

The cDNA library sizes differed between treatments and were normalized by DESeq2's coefficient (size factors), which ranged from 0.26 to 3.33, with the size factors of the pH 7.95 treatment being consistently low (Fig. S1). There were 59,353 contigs identified in the de novo transcriptome, with a N50 of 1,019 bp (Supporting Information). BUSCO analysis indicated that the transcriptome was relatively complete: of the 1,066 arthropod orthologs queried, the transcriptome contained 75.4% complete orthologs (71.5% single and 3.9% duplicate copies), with 18.1% fragmented and only 6.5% missing. Of 59,353 contigs, 19,790 (33%) were associated with GO classifications, with an average of 3.54 classifications per contig.

**Figure 1:**
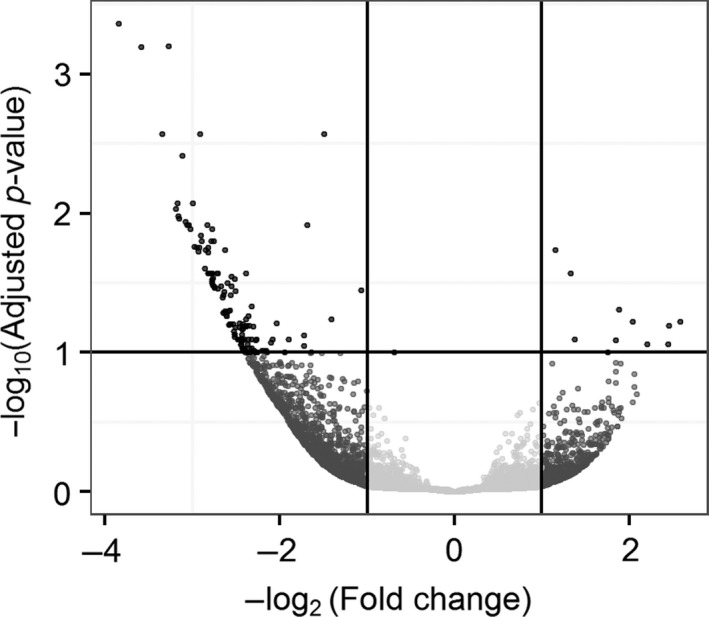
**“**Volcano plot” illustrating the selection of genes that were significantly differently expressed with pH in *C. glacialis*. Plotted are ‐log‐transformed Ward test *p*‐values for the slope of expression with pH, corrected for multiple tests by the Benjamini‐Hochberg method, and presented as false discovery rate (FDR), and –(log2 fold change) of expression over one unit of pH. The log2 fold change (LFC) is negative to indicate that those with a negative value are downregulated as pH is reduced. Significantly differently expressed genes (DECs, in darkest gray) were defined as those with |LFC| > 1 and FDR < 0.1

**Figure 2:**
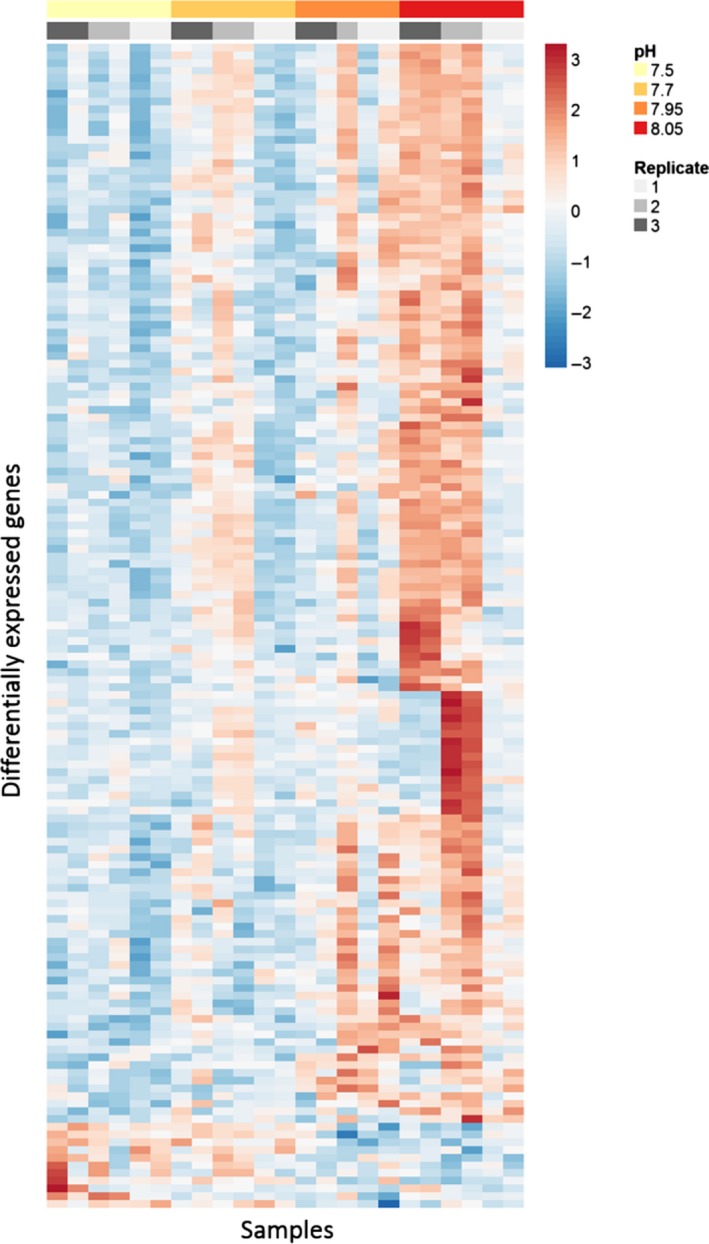
Heatmap of rlog‐transformed gene expression in *C. glacialis* at different pH treatments, centered, and standardized by gene for each sample (in columns). The color of the cell indicates relative gene expression, with red being higher than the gene's average, and blue lower. Only the 151 significantly differentially expressed genes are shown. The pH treatment and biological replicate (tank) are indicated in shaded red‐yellow and gray, respectively, above the heatmap. On the left vertical axis, genes are arranged according to their position in the hierarchical clustering (similarity measure was Pearson correlation coefficient; clustering by complete linkage)

Of these 59,353 contigs, 151 were identified as significantly differently expressed with pH between pH 7.5 and 8.05 (Figure 1). The majority of the DE contigs (140 of 151; 93%) were downregulated as pH decreased (hereafter referred to as downregulated), with 11 contigs upregulated at lower pH (Figure 1). Biological replicates (tanks) and, to a greater degree, technical replicates (different animals from the same tank) showed similar expression of many DECs, although certain tanks and technical replicates show different expression in parts of the profile (Figure 2).

**Figure 3:**
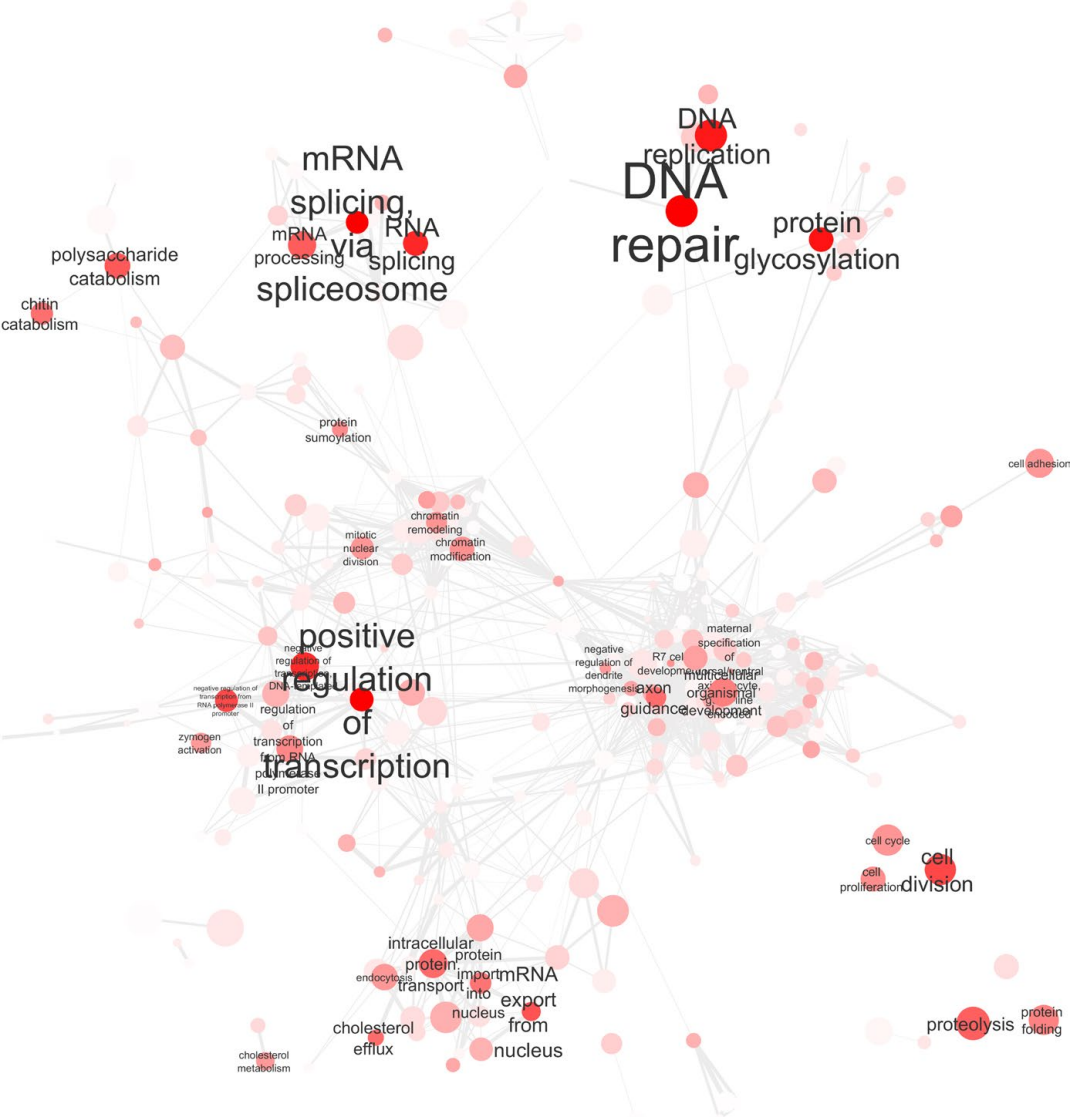
Cytoscape network of the 350 most significant downregulated biological process Gene Ontology (GO) terms in *C. glacialis*, linked by SimRel semantic similarity of the terms by ReViGO. The color of each GO term node (from white to dark red) indicates the adjusted *p*‐value, with more significant terms in darker red. Likewise, the larger the label name, the more significant the adjusted *p*‐value. The size of each node indicates the size of the GO term in the entire UniProt database

Numerous cellular processes (1,001 biological process GO terms, 358 molecular function GO terms and 16 KEGG pathways) were significantly downregulated with lowered pH. Clustering these GO terms by their similarity reduced the list to 87 key biological processes, including DNA repair, cell adhesion, growth, cholesterol homeostasis, chromatin modification, development, protein glycosylation, endocytosis, mRNA splicing, regulation of transcription, regulation of protein stability, protein folding, and signaling pathways (Figure 3; Table S2). There were 184 key downregulated molecular functions, including transcription factors, serine‐type endopeptidase activity, receptor activity, oxidoreductase activity, ubiquitin‐protein transferase activity, chitinase activity, and helicase activity (Fig. S3 and Table S3). The KEGG pathways significantly downregulated at lowered pH included metabolic pathways, lysosome, cytochrome P450, amino acid metabolism, spliceosome, peroxisome, and RNA transport (Table S4).

**Figure 4:**
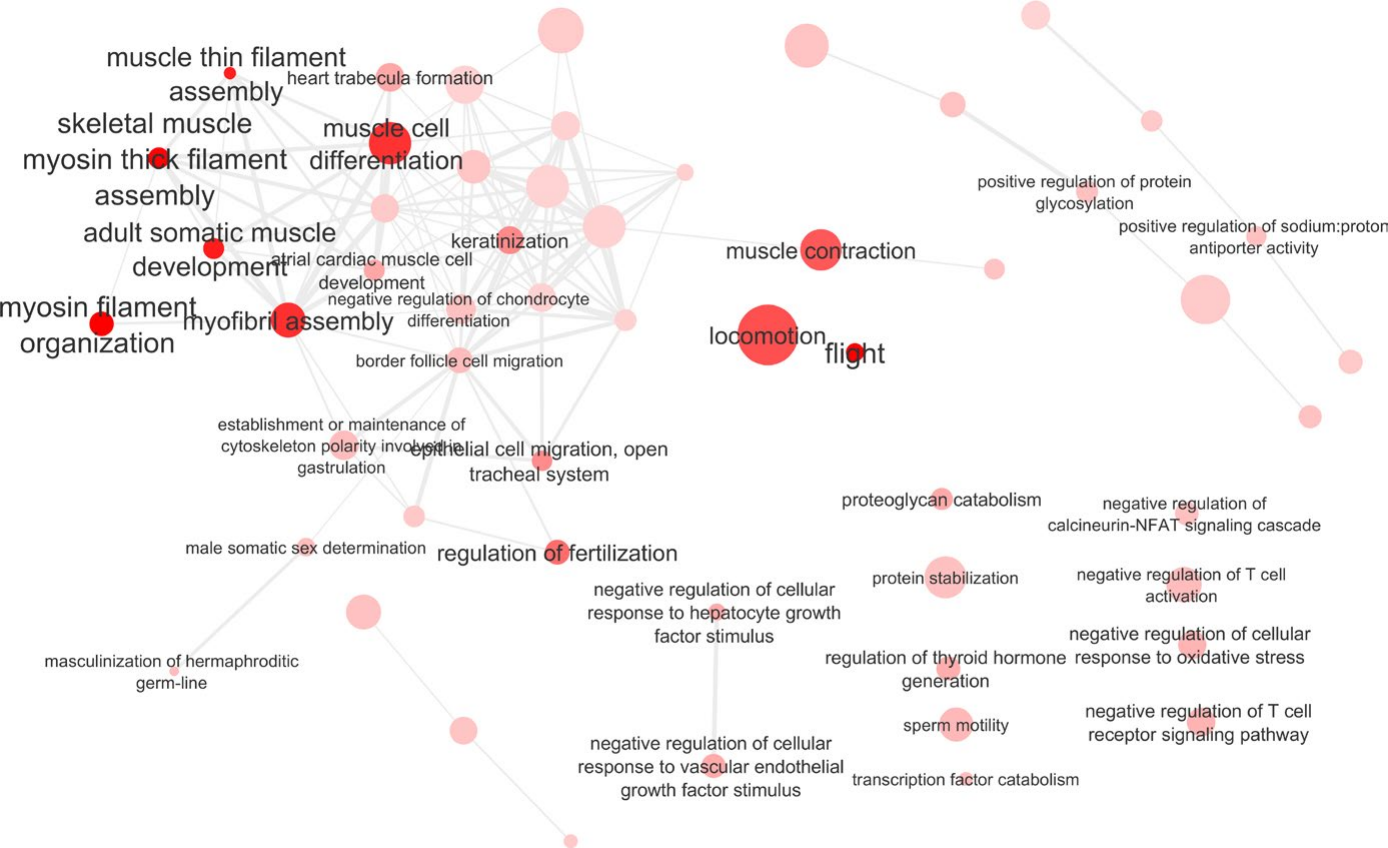
Cytoscape network of the most significant upregulated biological process Gene Ontology (GO) terms in *C. glacialis*, linked by SimRel semantic similarity of the terms by ReViGO. Plot components as in Figure 3

As fewer contigs were significantly upregulated at lowered pH, fewer GO terms (91 biological process GO terms and 39 molecular function GOs) and KEGG pathways (13) were identified as significantly upregulated. The condensed list of biological process GOs that were upregulated at lowered pH include myosin filament organization, locomotion, proteoglycan catabolic process, muscle contraction, transposition, regulation of sodium:proton antiporter, protein deamination, and keratinization (Figure 4, Table S5). Molecular function GOs upregulated at low pH included motor activity, calmodulin‐dependent protein kinase activity, and MAP kinase phosphatase activity (Fig. S4, Table S6). The upregulated KEGG pathways included tight junction, cardiac muscle contraction, pathogenic *Escherichia coli* infection, ribosome, AMPK signaling pathway, plant‐pathogen interaction, and regulation of actin cytoskeleton (Table S7).

## 4 DISCUSSION

In a parallel study on the same experimental cultures, *C. glacialis* nauplii were able to develop, grow, and respire at normal rates in CO_2_‐acidified sea water down to pH 7.5 (Bailey et al., 2016). On the organismal level, therefore, *C. glacialis* nauplii were tolerant of pH levels relevant for future ocean acidification scenarios. However, in the present study, significant changes occurred at the transcriptomic level, despite the lack of an observable response on organismal‐level vital rates (Bailey et al., 2016). Altered gene expression at low pH indicated regulation of ion exchange activity, DNA transcription, molecular chaperones, and processes involved in locomotor activity in N6 nauplii. This may have allowed *C. glacialis* to phenotypically buffer the change in pH, thus maintaining normal nauplii development. By measuring the response to low pH at multiple levels of biological organization, both organismal and molecular, we detected physiological changes in response to low pH that were undetected in organismal measures. Additionally, there continues to be considerable inconsistency in the measured responses of marine organisms to low pH, even within taxa (Kroeker et al., 2010; Kroeker, Kordas, & Crim, 2013). Therefore, these results on the molecular response of an apparently tolerant species will improve our understanding of the physiological basis of low pH tolerance that is found in some, but not all species.

### 4.1 Percentage of transcriptome altered

Investigating the expression of single genes identified 151 genes as significantly differently expressed with pH between pH 7.5 and 8.05, representing ~0.25% of the genes in the transcriptome. The *de novo* transcriptome created in this study was of similar size to that of another *C. glacialis* assembly (55,562 contigs, Ramos et al., 2015) and that of *Calanus sinicus* (56,809, Ning, Wang, Li, & Sun, 2013) but larger than another *C. glacialis* transcriptome (36,880, Smolina et al., 2015). Our transcriptome had a higher N50 than the previous *C. glacialis* transcriptomes. Combined with a good BUSCO completeness measure, this indicates that our transcriptome is of high quality. While ~0.25% of the transcriptome significantly differentially expressed is a low percentage, it is similar to other studies using nontargeted investigations of the entire transcriptome: in a copepod in response to heat stress (0.88%; Schoville, Barreto, Moy, Wolff, & Burton, 2012), an Antarctic fish in response to pH and heat stress (0.08%–1.0%; Huth & Place, 2016), corals in response to heat stress (0.4%–0.7%; Barshis et al., 2013), and sea grass in response to heat stress (0.8%; Franssen et al., 2011). However, other studies have found greater percentages of the transcriptome affected (e.g., corals in response to low pH; 12%–19%; Moya, Huisman, & Ball, 2012; or heat shock; 27%; Seneca & Palumbi, 2015). The number of genes that have a significant impact on phenotype is very low, and the percent that modify sensitivity to abiotic stressors is even smaller (0.6%–2% in Brewer's yeast (*Saccharomyces cerevisae*) deletion strains) (Feder & Walser, 2005; Auesukaree et al., 2009; Evans, 2015). This indicates that even changes in a small percent of the transcriptome can have considerable physiological impact.

Acclimation to altered pH over the exposure period may have reduced the number of differentially expressed genes over time, as differential gene expression is often highest immediately after the introduction of the stressor. Huth and Place (2016) found that the degree of altered gene expression in fish exposed to pH and temperature stress decreased with time, being highest 7 days after the start of exposure and decreasing by 80%–90% at 28 and 56 days postexposure start (from 1.0% to 0.1% to 0.2%, respectively). Evans, Chan, Menge, and Hofmann (2013) found a strong reduction in the number of genes differentially expressed with time in purple sea urchin larvae (*Strongylocentrotus purpuratus*), from 153 at 44 hr of age to only five genes at 96 hr. The acclimation of gene expression can occur over even shorter timescales. Seneca and Palumbi (2015) found many coral genes that were differentially expressed 5 hr after a simulated heat wave but not after 20 hr. The copepods in this study were exposed for 35–38 days before sampling and therefore may have had larger differences in gene expression between high and low pH immediately after the pH treatments began. Investigating gene expression in N6 nauplii that have been chronically exposed to lowered pH throughout their lives, from the time of hatching through all naupliar stages, reflects an acclimation to low pH rather than the short‐term response. Short‐term gene expression changes are unlikely to have a long‐term effect on fitness, which is why we chose to focus on the long‐term, acclimation response, which more closely reflects how gene expression may respond to near‐term climate change with increased pCO_2_ in natural systems.

Further, mortality was not tracked in these experiments and is known to be high in broadcast spawned copepod larvae (Kiørboe & Sabatini, 1994). Our measurements, both on the organismal level, as discussed in Bailey et al. (2016), and on the transcriptomic level may therefore reflect the surviving, low pH‐tolerant portion of the population. However, this does not change the importance of these data for understanding the transcriptomic basis of tolerance to low pH.

### 4.2 Regulation of gene set expression

Investigating the coordinated expression of sets of genes revealed that a wide range of biological processes were affected by pH in *C. glacialis* N6 nauplii. Many of the biological processes downregulated by pH are key components of the evolutionarily conserved, universal cellular stress response (CSR; Kültz, 2003, 2005). Cells activate the CSR in response to damage and deformation of macromolecules (proteins, DNA, RNA, and membrane lipids) that occurs as a result of a broad range of stressors (Kültz, 2003). The universal stress response includes the following functions: redox regulation, DNA damage sensing and repair, molecular chaperones, protein degradation, fatty acid/lipid metabolism, and energy metabolism (Kültz, 2005). In a meta‐analysis of 14 studies of transcriptomic responses of oysters (genera *Crassostrea*,* Ostrea*, and *Saccostrea*) to various stressors, Anderson et al. (2015) defined 12 intracellular processes affected by stress as follows: cell cycle, communication/signaling, cytoskeleton, extracellular matrix, immunity, metabolism, nucleic acid regulation (DNA repair), protein regulation (protease, proteosome), membrane transport, stress (chaperones, detox, redox), transcription, and translation. The processes significantly downregulated in *C. glacialis* at lower pH include nearly all of the main functionalities of the CSR, including cell cycle regulation, metabolism, DNA repair, protein modification and degradation, redox regulation, and transcription regulation (Table 1).

**Table 1:** Key Gene Ontology (GO) terms and Kyoto Encylopedia of Genes and Genomes (KEGG) pathways regulated significantly with pH in *C. glacialis* and associated with the universal cellular stress response, as outlined by Kültz (2005) and Anderson et al. (2015). Negative log‐odd ratio (LOR) indicates downregulation with lower pH. Number of contigs in the GO term or KEGG pathway (#) and adjusted *p*‐value (*p*) are also presented

Kültz, 2005	Anderson et al., 2015		#	*p*	LOR
*Energy metabolism*	*Metabolism: lipid binding, lipid metabolism, electron transport, glycolysis, TCA cycle*				
*Fatty acid lipid metabolism*	GO:0010906	regulation of glucose metabolic process	138	4.2E−03	−0.40
	GO:0006099	tricarboxylic acid cycle	75	5.2E−03	−0.53
	GO:0042157	lipoprotein metabolic process	25	2.9E−03	−0.99
	GO:0005975	carbohydrate metabolic process	131	1.4E−07	−0.77
	GO:0042632	cholesterol homeostasis	63	8.3E−06	−0.94
	GO:0004467	long‐chain fatty acid‐CoA ligase activity	80	4.8E−03	−0.52
	GO:0045922	negative regulation of fatty acid metabolic process	2	5.2E−03	−2.70
	KEGG 1100	metabolic pathways	1,950	4.4E−35	−0.47
*DNA repair and damage sensing*	*Nucleic acid repair: DNA repair*				
	GO:0006281	DNA repair	268	9.9E−18	−0.88
	GO:0003684	damaged DNA binding	56	1.2E−02	−0.56
	GO:0006974	cellular response to DNA damage stimulus	222	3.4E−09	−0.66
	GO:0042769	DNA damage response, detection of DNA damage	5	1.2E−02	−1.76
	GO:0042770	signal transduction in response to DNA damage	13	3.3E−02	−0.99
	GO:0000724	double‐strand break repair via homologous recombination	67	7.2E−04	−0.69
	GO:0000731	DNA synthesis involved in DNA repair	17	1.5E−02	−0.99
*Protein degradation*	*Protein regulation: protease, proteosome*				
	GO:0004843	ubiquitin‐specific protease activity	59	1.1E−03	−0.71
	GO:0010499	proteasomal ubiquitin‐independent protein catabolic process	21	1.9E−05	−1.51
	GO:0006508	Proteolysis	260	5.9E−09	−0.60
	GO:0031647	regulation of protein stability	54	6.5E−04	−0.77
	GO:0010499	proteasomal ubiquitin‐independent protein catabolic process	21	1.9E−05	−1.51
	GO:0006511	ubiquitin‐dependent protein catabolic process	138	2.5E−05	−0.59
	*Transmembrane gates, channels, pumps*				
	GO:0005231	excitatory extracellular ligand‐gated ion channel activity	2	3.0E−02	−2.24
	GO:0005245	voltage‐gated calcium channel activity	49	2.5E−02	−0.53
	GO:0005262	calcium channel activity	100	5.8E−03	−0.45
*Redox*	*Detox and intracellular stress: oxidative stress*				
	GO:0045454	cell redox homeostasis	87	2.2E−04	−0.66
	GO:1900408	negative regulation of cellular response to oxidative stress	1	5.1E−03	2.98
	GO:1900034	regulation of cellular response to heat	25	6.1E−05	−1.32
*Molecular chaperones*	*Chaperones*				
	GO:0061077	chaperone‐mediated protein folding	29	1.2E−02	−0.78
	GO:0031072	heat shock protein binding	23	4.6E−02	−0.69
	GO:0006457	protein folding	172	1.2E−07	−0.67
	*Transcription: nucleosomes*				
	GO:0006351	transcription, DNA‐templated	1,855	8.1E−20	−0.35
	GO:0006355	regulation of transcription, DNA‐templated	1,134	4.0E−15	−0.38
	GO:0016568	chromatin modification	164	4.5E−07	−0.66
	GO:0006337	nucleosome disassembly	10	4.9E−03	−1.45
	GO:0000398	mRNA splicing, via spliceosome	183	2.2E−13	−0.91
	GO:0010468	regulation of gene expression	118	4.0E−04	−0.54
	KEGG 3040	spliceosome	173	1.0E−13	−0.95
	*Translation, post‐translational processing: ribosomes*				
	GO:0006486	protein glycosylation	203	6.8E−12	−0.81
	GO:0003743	translation initiation factor activity	67	1.1E−02	−0.51
	GO:0006446	regulation of translational initiation	36	2.6E−03	−0.84
	*Cell cycle: differentiation, proliferation, apoptosis, death*				
	GO:0060548	negative regulation of cell death	47	1.9E−03	−0.76
	GO:0030154	cell differentiation	293	1.3E−03	−0.31
	GO:0051301	cell division	352	7.5E−10	−0.54
	GO:0007049	cell cycle	191	6.6E−07	−0.60
	GO:0007050	cell cycle arrest	71	4.7E−02	−0.38
	GO:0051302	regulation of cell division	6	4.1E−02	−1.36
	GO:0042127	regulation of cell proliferation	116	1.7E−05	−0.66
	GO:0045595	regulation of cell differentiation	25	1.2E−02	−0.84
	GO:0006915	apoptotic process	397	1.6E−03	−0.26
	GO:1902177	pos. reg. of oxidative stress‐induced intrinsic apoptotic signaling	2	2.1E−02	−2.35
	*Communication: membrane receptors, intracellular signaling*				
	GO:0010649	regulation of cell communication by electrical coupling	4	1.5E−02	−1.87
	GO:0030518	intracellular steroid hormone receptor signaling pathway	11	2.5E−02	−1.13
	GO:2000323	negative regulation of glucocorticoid receptor signaling pathway	4	2.5E−02	−1.76
	*Cytoskeleton: vesicular trafficking*				
	GO:0030866	cortical actin cytoskeleton organization	32	5.8E−03	−0.81
	GO:0070062	extracellular vesicular exosome	1,506	4.8E−23	−0.42
	*Extracellular matrix*				
	GO:0030198	extracellular matrix organization	103	5.8E−03	−0.45
	*Immunity*				
	GO:0045087	innate immune response	156	5.1E−06	−0.61
	GO:0006955	immune response	107	1.7E−03	−0.50
	GO:0002682	regulation of immune system process	7	4.8E−03	−1.69

### 4.3 Downregulation of stress genes

Interestingly, these CSR‐related GO terms and KEGG pathways were downregulated in response to low pH, rather than upregulated. In a short‐term response to stress, an upregulation in CSR proteins (or a transcriptomic upregulation of genes coding for them) is expected (Tomanek & Somero, 2000; Kültz, 2003), as has been reported in numerous marine organisms (Lauritano, Procaccini, & Ianora, 2012; Tomanek, 2014; Huth & Place, 2016). However, several studies show both up‐ and downregulation of CSR‐related genes in response to a range of abiotic stressors (Anderson et al., 2015; Goncalves et al., 2016). Downregulation of stress‐related genes has been reported in the Sydney rock oyster (*Saccostrea glomerata*) in response to lowered pH (Goncalves et al., 2016). Downregulation of stress‐related genes when exposed to a stressor may also be a characteristic of tolerant populations. In a study comparing wild oysters to a selectively bred line that had developed tolerance to low pH, Goncalves et al. (2016) found that the line that had developed tolerance to low pH reacted to decreased pH by downregulating genes associated with stress response. In contrast, the sensitive wild population upregulated the same genes. Raising wild oysters in low pH water for only three generations produced oysters that responded to low pH stress in the same way as the selectively bred line (selected for fast growth and disease immunity over seven generations), namely downregulation of stress genes (Parker et al., 2012). Similarly, gene expression of an Antarctic fish, whose adaptation to cold waters involves constitutively elevated stress‐related genes, shows a downregulation of those genes when exposed to temperature stress (Huth & Place, 2016). Heat‐tolerant strains of *Daphnia pulex* showed a general downregulation of genes involved in transcription, translation, DNA replication, DNA repair, and core metabolic pathways in response to heat stress, a response not present in heat‐sensitive strains (Yampolsky et al., 2014). Downregulation of helicase, involved in DNA replication, was also involved in tolerance to low pH in *Pseudocalanus acuspes* copepods which had gained some tolerance to low pH after being exposed for three generations (De Wit, Dupont, & Thor, 2015). In this context, the downregulation of the majority of DECs in this study can reasonably be interpreted as an indication that *C. glacialis* is naturally tolerant of lowered pH, which is consistent with the organismal‐level tolerance measured on the same nauplii (Bailey et al., 2016).

### 4.4 General downregulation

*Calanus glacialis* nauplii showed an overall downregulation of transcription in the contigs whose expression was significantly related to pH. Of the 151 differentially expressed contigs, 93% were downregulated at low pH. Other studies have also found a general downregulation of proteins and genes in response to low pH stress (Todgham & Hofmann, 2009; Evans & Watson‐Wynn, 2014; Dineshram et al., 2016). In a meta‐analysis of eight papers evaluating the transcriptomic response of sea urchins to low pH, Evans and Watson‐Wynn (2014) found an overwhelming downregulation, with 80% of differentially expressed genes downregulated at low pH (Evans & Watson‐Wynn, 2014). Notably, in one paper, 90%–100% of the differentially expressed genes were downregulated (Todgham & Hofmann, 2009). This included cellular stress response genes, metabolism, and apoptosis in urchins exposed to lowered pH, both at pH 7.96 and 7.88. In larvae of the Pacific oyster (*Crassostrea gigas*), 70% of the differentially expressed proteins were downregulated at low pH, overshadowing the influence of temperature and salinity stressors (Dineshram et al., 2016). They suggested that while the function of general downregulation of protein synthesis and transcription at low pH is unknown, protein synthesis is both energetically costly (Rolfe & Brown, 1997; Wieser & Krumschnabel, 2001; Sokolova, Frederich, Bagwe, Lannig, & Sukhotin, 2012) and produces reactive oxygen species (ROS; Tomanek, 2014). Thus, downregulating protein synthesis at low pH may be a strategy to conserve energy and minimize oxidative damage from ROS. If the downregulation of transcription of some genes observed in *C. glacialis* nauplii indicates a downregulation of their protein synthesis, this energy conservation strategy may at least partially explain why we did not observe an increase in metabolic rate in the larvae during this experiment (Bailey et al., 2016).

The direction of gene regulation (up‐ or downregulation) can change with time after onset of stress, with some genes being initially upregulated and then later downregulated in response to heat stress in *C. gigas* (Meistertzheim, Tanguy, Moraga, Thébault, & Thebault, 2007) or pH and heat stress in the staghorn coral *Acropora aspera* (Ogawa, Bobeszko, Ainsworth, & Leggat, 2013). In response to stress, *Daphnia pulex* shows an initial upregulation and then a longer‐term downregulation of stress‐related genes (Heckmann, Sibly, & Connon, 2008). This may indicate that the downregulation we measured is a result of the long‐term stress exposure prior to sampling. An upregulation of the same stress‐related genes may have occurred earlier in the exposure. Depending on the half‐life of the proteins they code for, an early upregulation of these stress genes could have produced enough protein that the transcription of more mRNA had been negatively regulated by the time we sampled, thus explaining the general downregulation of many stress‐related genes. An exciting future avenue for research would thus be investigation of protein content in combination with transcript‐level data over a temporal sampling regime.

### 4.5 Upregulation

Among the processes upregulated at low pH was the positive regulation of sodium:proton antiporter activity (GO:0032417). The elevated proton concentrations in low pH sea water likely increase the proton concentrations in the extracellular fluid of *C. glacialis* (Pörtner, 2008; Whiteley, 2011; Melzner, Thomsen, & Koeve, 2013). Aquatic crustaceans maintain pH homeostasis primarily via ion transport (Henry & Wheatly, 1992) and the sodium:proton antiporter is key to maintaining intracellular pH (Casey, Grinstein, & Orlowski, 2010). By removing a proton in exchange for a sodium ion, sodium:proton antiporters directly regulate intracellular pH. *Calanus glacialis* appears to increase the transcription of genes which upregulate the activity of these antiporters in response to low pH sea water. Other organisms upregulate sodium:proton antiporters in response to low pH water (fish: Catches, Burns, Edwards, & Claiborne, 2006; Huth & Place, 2016) or other ion pumps (sodium:potassium ATPase; Pan, Applebaum, & Manahan, 2015), while corals (Moya et al., 2012; Evans et al., 2013) and urchins (Todgham & Hofmann, 2009; Stumpp, Dupont, Thorndyke, & Melzner, 2011) did not upregulate them at low pH, potentially being able to regulate pH sufficiently with the existing antiporters. Our findings indicate that sodium:proton pumps were upregulated by *C. glacialis* nauplii in an attempt to maintain cellular pH homeostasis at low pH, although the wide range of altered gene expression at low pH indicates that they did not succeed in fully maintaining homeostasis. Unlike active ion pumps which hydrolyze ATP to move ions across a membrane, sodium:proton antiporters passively exchange ions, a process that does not require the expenditure of metabolic energy (Aharonovitz, Demaurex, Woodside, & Grinstein, 1999). The upregulation of these antiporters, therefore, does not represent an increased energetic cost of pH homeostasis for *C. glacialis*, indicating that it may be an energetically sustainable component of their response to low pH.

The upregulation of sodium:proton antiporters is also of interest for general copepod physiology. Little is known about the osmoregulation of copepods, and marine copepods are considered osmoconformers (Mauchline, 1998). However, the calanoid copepod *Eurytemora affinis* has the capability to osmoregulate via the ion‐transport enzymes V‐type H^+^‐ATPase and Na^+^/K^+^‐ ATPase, an ability that allowed it to colonize freshwater from marine environments (Lee, Kiergaard, Gelembiuk, Eads, & Posavi, 2011). Our findings with *C. glacialis* lend additional support to the osmoregulatory capacity of marine copepods, possibly via sodium:proton antiporters.

Also upregulated were molecular functions related to locomotion, actin‐dependent ATPase activity and structural constituents of cuticle and muscle, suggesting altered locomotory muscle use. While we did not measure swimming behavior or escape response speed, larvae of other crustaceans including the American lobster (*Homarus americanus*) show higher swimming speeds at low pH (Waller, Wahle, Mcveigh, & Fields, 2016). Actinomyosin ATPase is a considerable component of the cellular energy budget (Rolfe & Brown, 1997). If an organism is energy‐limited, upregulation of this energy‐demanding process may lead to less energy for fitness‐related processes such as growth and development, although we did not detect declines in developmental time, body weight, or C:N ratio, or an increase in respiration in the *C. glacialis* nauplii with low pH (Bailey et al., 2016).

### 4.6 Transcriptomic expression of tolerance

Understanding the transcriptomic response of an organism to an environmental variable provides information on the extent to which the variable induces a stress response and which physiological functions are induced by that stress response, indicating whether they are components of the universal stress response or specific to that stressor or species. Altered gene expression can indicate stress but also the ability to deal with stressors: Some indicate beneficial responses and some maladaptive.

In evaluating altered gene expression in response to potential stressors, it is important to consider that not all genes have a similar ability to affect fitness. For many genes, the silencing of a single gene produces no phenotypic change at all (Feder & Walser, 2005; Evans, 2015), while other genes have a disproportionate effect on fitness because they are involved in regulatory networks and therefore have the capacity to influence many more proteins than just the one protein that they code for. Additionally, genes which are differentially expressed in response to a stress are not necessarily the same as those that confer tolerance to the same stress (de Nadal, Ammerer, & Posas, 2011; Gibney, Lu, Caudy, Hess, & Botstein, 2013; Giaever & Nislow, 2014; De Wit et al., 2015; Evans, 2015). Two types of genes that usually are not differentially expressed under stress, but whose expression have far‐reaching effects that can confer tolerance to that stress, are those involved in chromatin modification and cell signaling (Gibney et al., 2013; Jarolim, Ayer, & Pillay, 2013; Evans, 2015). Interestingly, in *C. glacialis*, genes controlling both chromatin modification (GO:0016568) and intracellular signaling transduction (GO:0035556) were significantly affected by pH, being downregulated in lower pH. As both chromatin and signaling pathways have the potential to regulate a large number of other genes and proteins, this indicates that large physiological changes may have been altered in *C. glacialis* via these transcriptional changes. Chromatin modification affects DNA replication, transcription, and repair and is a critical component of an organism's response to stress (Smith & Workman, 2012) and acclimation to their environment (van Zanten, Tessadori, Peeters, & Fransz, 2013). Changes to chromatin can also be passed on to offspring via epigenetic inheritance, thus potentially affecting the response of future generations to the environment (Campos, Stafford, & Reinberg, 2014). Indeed, epigenetic acclimation to stressors may be possible in copepods. Transgenerational effects (either adaptation or epigenetic acclimation) in the copepod *Pseudocalanus acuspes* alleviated the negative effect of low pH after two generations in low pH (Thor & Dupont, 2015). While the temporal duration of the chromatin modifications in *C. glacialis* at low pH, or their ability to be epigenetically passed on to the next generation, is unknown, they may play a part in both acclimatization within a single lifetime and over several generations.

The degree of altered gene expression during stress does not necessarily correlate with a reduction in performance or fitness. Activation of the cellular stress response immediately after the onset of the stress can help protect cells against protein unfolding, DNA damage, and oxidative damage (Kültz, 2003, 2005). Differential gene expression can therefore indicate adaptive responses and contribute to tolerance. Smolina et al. (2015) suggested that a strong transcriptomic response to stress underpins tolerance at the organismal level (i.e., respiration, feeding, mortality). They showed that *C. finmarchicus* strongly altered their gene expression during heat stress, while *C. glacialis* exhibited a near lack of response, altering the expression of only one gene transcript. As a result, *C. finmarchicus* showed mild changes in feeding and mortality at increased temperature, while *C. glacialis* was strongly affected, reducing grazing and showing higher mortality rates than *C. finmarchicus* under heat stress. In contrast to Smolina et al.'s (2015) findings on *C. glacialis’* mild gene expression response to increased temperature, we found that *C. glacialis* exhibited a significant change in gene expression in response to low pH. Similar to their findings, we found that significant regulation of gene expression was associated with tolerance to stress. The gene expression regulation in *C. glacialis* nauplii is associated with tolerance of low pH for a range of organismal‐level vital rates (Bailey et al., 2016).

In evaluating the implication of transcriptomic regulation on tolerance, it is important to consider the time course of changes in gene expression. With the emergence of transcriptomics studies in investigating organismal responses to environmental stressors, the concept of “transcriptomic resilience” has recently been introduced (Franssen et al., 2011). They propose that the quick return to normal gene expression after exposure to a stressor is a sign that an organism can tolerate environmental changes. Seneca and Palumbi (2015) support this theory, showing that gene expression in corals from a tolerant population returned to control levels to a greater extent than in corals from a sensitive population, in response to heat stress. Those individuals whose gene expression returned to control levels exhibited less bleaching than those whose gene expression remained altered. Tracking the temporal signal of differential gene expression is therefore of utmost importance when comparing the stress tolerance of different species or the effect of different stressors. To achieve this, we suggest that future studies on gene expression responses to stressors collect samples over time, starting immediately after exposure initiation, in order to track the temporal change in gene expression.

The concentration of specific proteins is not only the result of transcription, but also the balance between mRNA degradation, translation, and protein degradation (Schwanhäusser, Busse, & Li, 2011). While stress can alter translation (Picard, Loubière, Girbal, & Cocaign‐Bousquet, 2013), mRNA degradation (Huch & Nissan, 2014), protein degradation (Flick & Kaiser, 2012), and protein activity (Krebs & Holbrook, 2001; Pan et al., 2015), transcriptomic regulation nonetheless can show a clear pattern of response to environmental stressors and, thus, indicate the involvement of genes and physiological processes in the organism's response (de Nadal et al., 2011; Evans & Hofmann, 2012; Evans & Watson‐Wynn, 2014; Anderson et al., 2015). With six measurements at each pH (five in the pH 7.95 treatment) and samples consisting of pooled individuals, we were able to conservatively select only the consistent signals in gene expression (Schurch et al., 2016). Furthermore, few papers examine differential gene expression over >2 pH treatments or use pH as a continuous variable (i.e., Barshis et al., 2013; Seneca & Palumbi, 2015; Huth & Place, 2016). By treating pH as a continuous variable (as opposed to a factor) and collecting data from four pHs, we narrowed the focus of this study to genes differentially expressed consistently over the investigated pH range, rather than those reacting to specific treatments, and reduced the chance of false positives.

Altered gene expression allowed *C. glacialis* to phenotypically buffer the change in pH, thereby maintaining normal nauplii development. The observed downregulation of stress‐related genes in response to stress appears to be a characteristic of stress‐tolerant populations and species. Additionally, the upregulation of energy‐neutral ion exchangers may contribute to *C. glacialis’* response to low pH without adding additional cost. In predicting the effects of ocean acidification on marine organisms, understanding the molecular mechanisms that confer tolerance to some species will support our ability to predict the effects of future ocean acidification on marine organisms.

## CONFLICT OF INTEREST

None declared.

## AUTHOR CONTRIBUTIONS

HIB, PT, HH, AB, DMF, JR: Concept and idea. AB, PDW, PT: Study design and methods. AB, PDW, SS, CT, RB: Data gathering. AB, PDW: Data analyses and interpretation. AB: Manuscript writing. PDW, HIB, PT, HH, DMF, JR: Manuscript interpretation/comments/revisions.
