# Supplementary material for: Regulation of gene expression is associated with tolerance of the Arctic copepod Calanus glacialis to CO 2‐acidified sea water
Source: Ecol Evol. 2017 Aug 2;7(18):7145–60. doi: 10.1002/ece3.3063 (PMC5606855; doi:10.1002/ece3.3063)
Supplement: Supplementary file 1 [file ECE3-7-7145-s001.docx]

SUPPORTING INFORMATION

Regulation of gene expression is associated with tolerance of the Arctic copepod *Calanus glacialis* CO_2_-acidified seawater

Allison Bailey^1, 2^, Pierre de Wit^3^, Peter Thor^1^, Howard I. Browman^4^, Reidun Bjelland^4^, Steven Shema^4^, David M. Fields^5^, Jeffrey A. Runge^6^, Cameron Thompson^6^, Haakon Hop^1, 2^

**Corresponding author:** Allison Bailey, Email: allison.bailey@npolar.no, phone: Telephone: +47 77 75 05 00, Telefax: +47 77 75 05 01


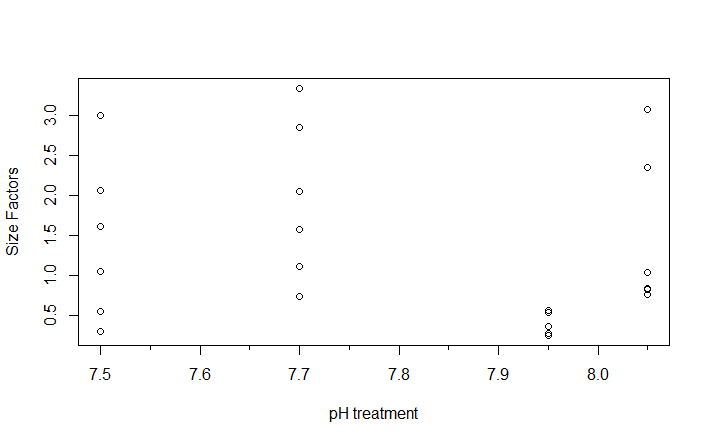


**Supplementary Figure 1.** To normalize *C. glacialis* cDNA library sizes between samples, each sample was multiplied by the DESeq2 coefficient “SizeFactor.” Here, size factors are presented by pH treatment.


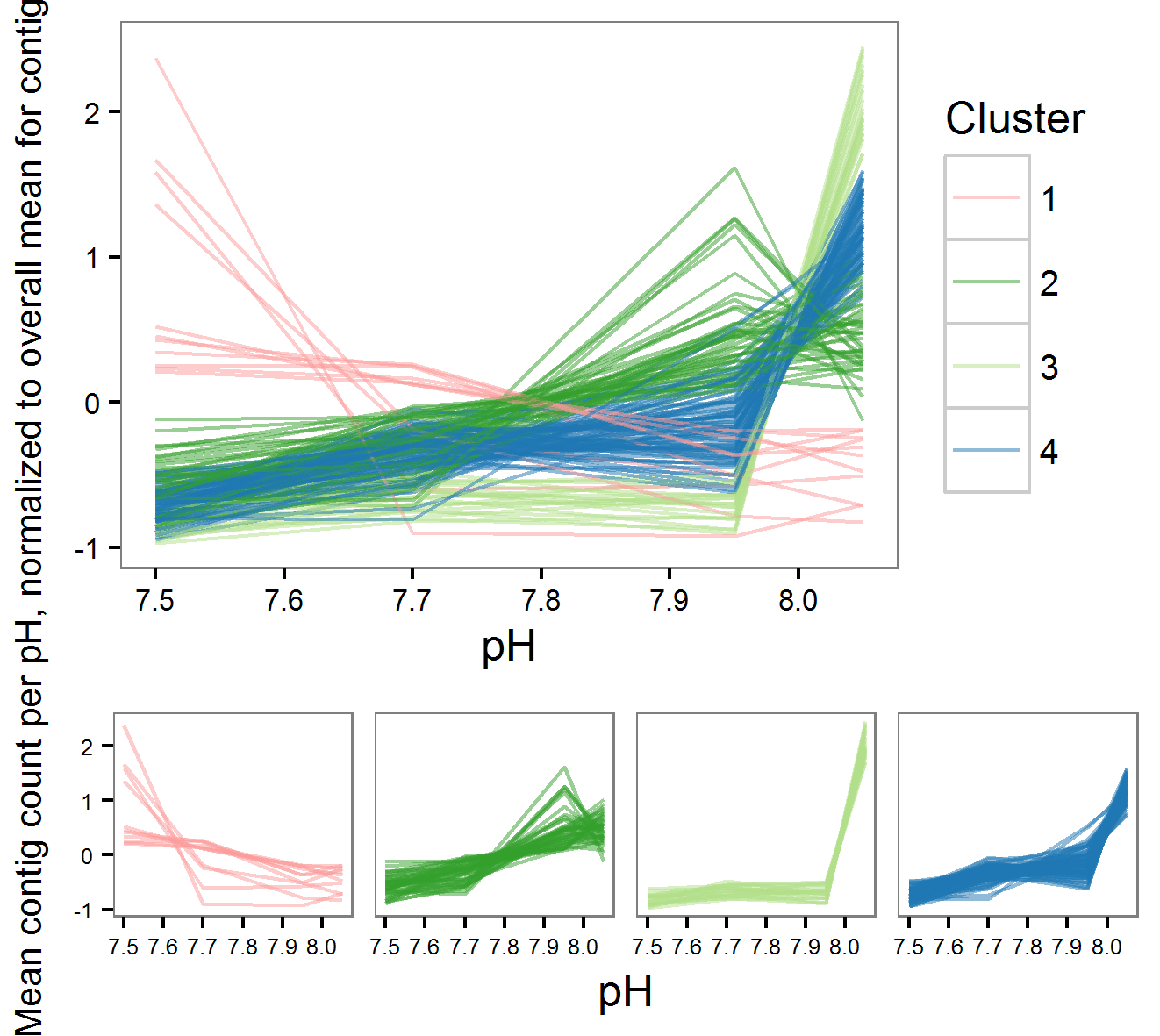


**Supplementary Figure 2.** Gene expression profiles in *C. glacialis* across four pH treatments. The 151 significantly differently expressed contigs (DECs) were grouped by expression profile using k-means clustering (Euclidean distance metric, 4 clusters, 1000 iterations, in R). Expression is the DESeq2-normalized count per contig and pH averaged over the 6 replicates divided by the contig’s overall mean and -1 (to center at zero). The lower panel shows expression in each cluster separately.

**
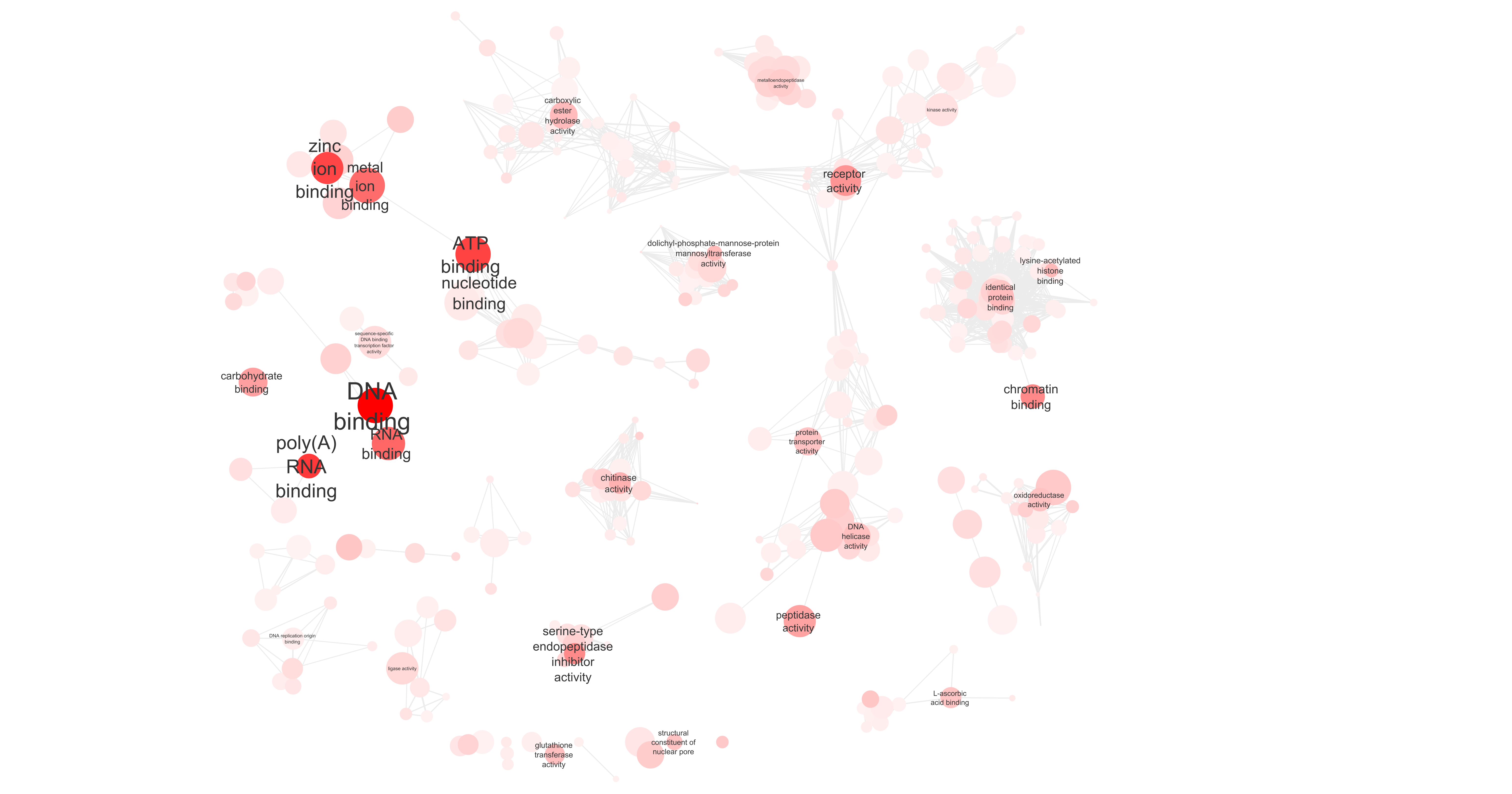
Supplementary Figure 3.** Cytoscape network of the 350 most significant down-regulated molecular function Gene Ontology (GO) terms in *C. glacialis*, linked by SimRel semantic similarity of the terms by ReViGO. The color of each GO term node (from white to dark red) indicates the adjusted p-value, with more significant terms in darker red. Likewise, the larger the label name, the more significant the adjusted p-value. The size of each node indicates the size of the GO term in the entire UniProt database.

**
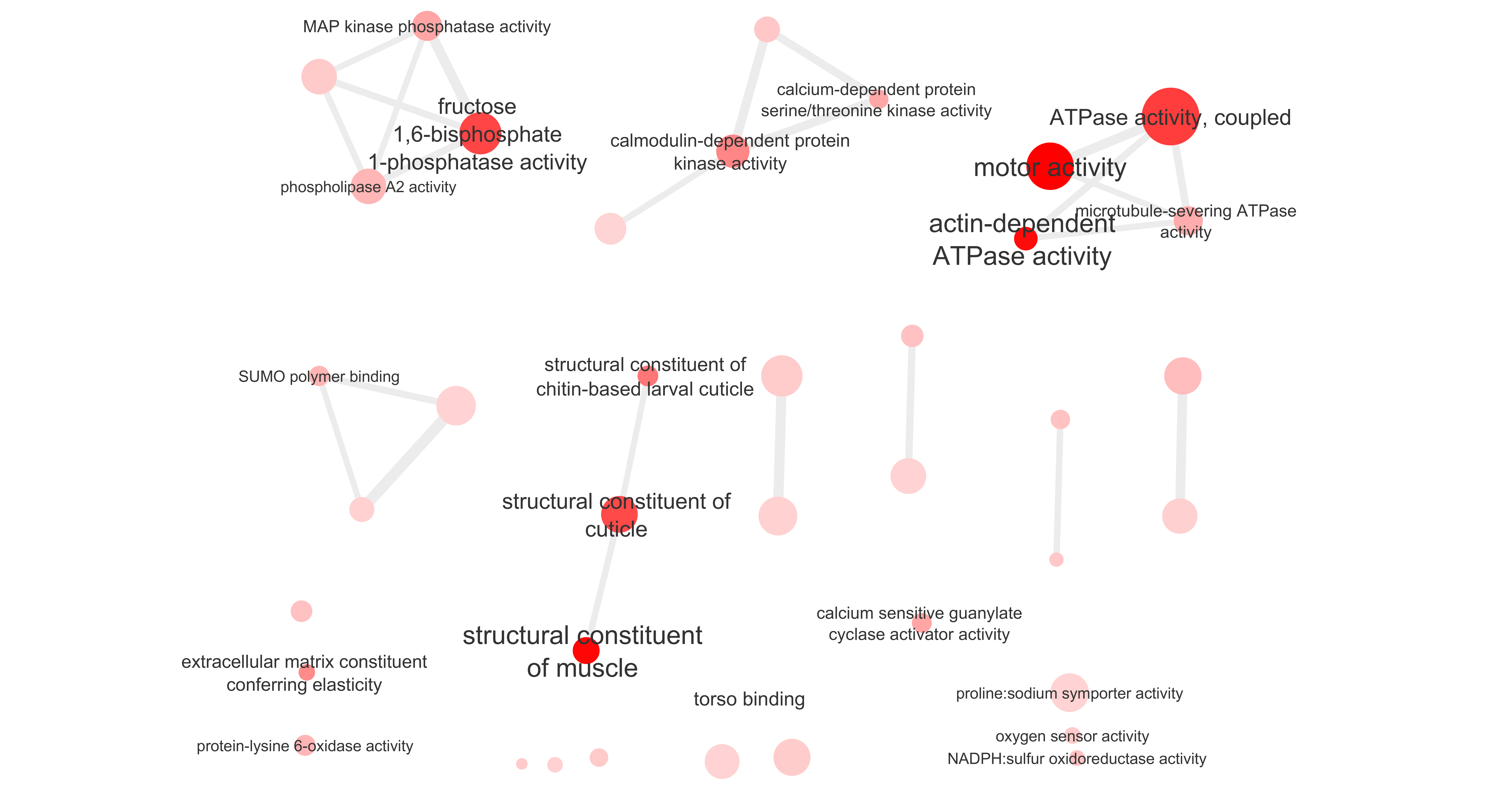
Supplementary Figure 4.** Cytoscape network of the 39 most significant up-regulated molecular function Gene Ontology (GO) terms in *C. glacialis*, linked by SimRel semantic similarity of the terms by ReViGO. The color of each GO term node (from white to dark red) indicates the adjusted p-value, with more significant terms in darker red. Likewise, the larger the label name, the more significant the adjusted p-value. The size of each node indicates the size of the GO term in the entire UniProt database.

| **Supplementary Table 1.** Overview of RNA-seq samples, with measures of duplicate reads. | | | | | | | | | | | |
| --- | --- | --- | --- | --- | --- | --- | --- | --- | --- | --- | --- |
| pH | Rep. | Tech. Rep. | Total Number of Reads | Number of unique reads with duplicates | Number of collapsed duplicates | Number of singletons | Total number of reads | Proportion singletons | Proportion duplicate reads | Proportion unique reads with duplicates | Proportion duplicate reads, after removal of ribosomal contigs |
| 8.05 | A | 1 | 6366266 | 1338817 | 26571456 | 3691555 | 30263011 | 0.1220 | 0.8780 | 0.0504 | 0.74 |
| 8.05 | A | 2 | 8243082 | 1449980 | 19507069 | 3950736 | 23457805 | 0.1684 | 0.8316 | 0.0743 | 0.64 |
| 8.05 | B | 1 | 10948129 | 993540 | 14562160 | 4068247 | 18630407 | 0.2184 | 0.7816 | 0.0682 | 0.67 |
| 8.05 | B | 2 | 15720891 | 1148333 | 23454108 | 4182193 | 27636301 | 0.1513 | 0.8487 | 0.0490 | 0.75 |
| 8.05 | C | 1 | 20231517 | 3646064 | 22405358 | 11358726 | 33764084 | 0.3364 | 0.6636 | 0.1627 | 0.61 |
| 8.05 | C | 2 | 16259933 | 2735746 | 15754829 | 10123157 | 25877986 | 0.3912 | 0.6088 | 0.1736 | 0.56 |
| 7.95 | A | 1 | 17938463 | 1222817 | 36265089 | 621977 | 36887066 | 0.0169 | 0.9831 | 0.0337 | 0.93 |
| 7.95 | A | 2 | 11759529 | 910078 | 24704707 | 1398404 | 26103111 | 0.0536 | 0.9464 | 0.0368 | 0.86 |
| 7.95 | B | 1 | 6768514 | 720365 | 26577062 | 2335863 | 28912925 | 0.0808 | 0.9192 | 0.0271 | 0.84 |
| 7.95 | C | 1 | 7125396 | 1349177 | 23491320 | 1475367 | 24966687 | 0.0591 | 0.9409 | 0.0574 | 0.77 |
| 7.95 | C | 2 | 6528068 | 630253 | 20378972 | 1684901 | 22063873 | 0.0764 | 0.9236 | 0.0309 | 0.81 |
| 7.7 | A | 1 | 7652902 | 1119937 | 21008704 | 3221646 | 24230350 | 0.1330 | 0.8670 | 0.0533 | 0.71 |
| 7.7 | A | 2 | 14088584 | 1321660 | 20180187 | 4585898 | 24766085 | 0.1852 | 0.8148 | 0.0655 | 0.69 |
| 7.7 | B | 1 | 19715922 | 3485725 | 20621675 | 12370026 | 32991701 | 0.3749 | 0.6251 | 0.1690 | 0.59 |
| 7.7 | B | 2 | 16887059 | 2800703 | 16021387 | 11364804 | 27386191 | 0.4150 | 0.5850 | 0.1748 | 0.56 |
| 7.7 | C | 1 | 19484164 | 2617153 | 28786450 | 5261835 | 34048285 | 0.1545 | 0.8455 | 0.0909 | 0.71 |
| 7.7 | C | 2 | 20354397 | 3161430 | 31335623 | 5883153 | 37218776 | 0.1581 | 0.8419 | 0.1009 | 0.72 |
| 7.5 | A | 1 | 18064815 | 3065776 | 20735073 | 10405990 | 31141063 | 0.3342 | 0.6658 | 0.1479 | 0.61 |
| 7.5 | A | 2 | 13063785 | 1969418 | 12359669 | 8421437 | 20781106 | 0.4052 | 0.5948 | 0.1593 | 0.53 |
| 7.5 | B | 1 | 14689007 | 667994 | 25580549 | 2064924 | 27645473 | 0.0747 | 0.9253 | 0.0261 | 0.85 |
| 7.5 | B | 2 | 8015131 | 1456459 | 27105919 | 4144647 | 31250566 | 0.1326 | 0.8674 | 0.0537 | 0.71 |
| 7.5 | C | 1 | 12920854 | 1776403 | 14373051 | 6973807 | 21346858 | 0.3267 | 0.6733 | 0.1236 | 0.56 |
| 7.5 | C | 2 | 7940009 | 897969 | 22624232 | 2744721 | 25368953 | 0.1082 | 0.8918 | 0.0397 | 0.75 |

| **Supplementary Table 2.** Biological process GO terms significantly associated with down-regulation in lowered pH, as evaluated by Babelomics’ logistic regression. The number of contigs that annotated to each GO term is given. P-values were adjusted using FDR. The 350 most significant GO terms are shown here (p<0.006) For all significant BP GOs, see Supplementary Info. Negative log odd ratio (LOR) indicates association with down-regulated contigs. Groupings of GO terms are based on their similarity (SimRel semantic similarity >0.5) by ReViGO. | | | | | | |
| --- | --- | --- | --- | --- | --- | --- |
| GO ID | GO Description | | # contigs | Adj *p-*value | LOR |  |
| GO:0006281 | **DNA repair** | | 268 | 9.92E-18 | -0.88 |  |
| GO:0034976 |  | *response to endoplasmic reticulum stress* | 72 | 1.11E-05 | -0.87 |  |
| GO:0006283 |  | *transcription-coupled nucleotide-excision repair* | 23 | 3.17E-03 | -1.03 |  |
| GO:0035556 |  | *intracellular signal transduction* | 303 | 5.90E-03 | -0.26 |  |
| GO:0006260 |  | *DNA replication* | 160 | 1.14E-11 | -0.90 |  |
| GO:0002682 |  | *regulation of immune system process* | 7 | 4.83E-03 | -1.69 |  |
| GO:0045087 |  | *innate immune response* | 156 | 5.06E-06 | -0.61 |  |
| GO:0006974 |  | *cellular response to DNA damage stimulus* | 222 | 3.37E-09 | -0.66 |  |
| GO:0006952 |  | *defense response* | 95 | 6.84E-05 | -0.68 |  |
| GO:0006955 |  | *immune response* | 107 | 1.69E-03 | -0.50 |  |
| GO:0006954 |  | *inflammatory response* | 92 | 1.84E-03 | -0.54 |  |
| GO:0000724 |  | *double-strand break repair via homologous recombination* | 67 | 7.19E-04 | -0.69 |  |
| GO:0007155 | **cell adhesion** | | 401 | 6.96E-07 | -0.41 |  |
| GO:0016339 |  | *calcium-dependent cell-cell adhesion via plasma membrane cell adhesion molecules* | 70 | 1.49E-04 | -0.76 |  |
| GO:0007156 |  | *homophilic cell adhesion via plasma membrane adhesion molecules* | 160 | 2.63E-06 | -0.62 |  |
| GO:0045585 |  | *positive regulation of cytotoxic T cell differentiation* | 2 | 3.28E-03 | -2.81 |  |
| GO:0044331 |  | *cell-cell adhesion mediated by cadherin* | 53 | 2.06E-05 | -0.98 |  |
| GO:0019886 | **antigen processing and presentation of exogenous peptide antigen via MHC class II** | | 25 | 2.58E-03 | -1.01 |  |
| GO:0040007 | **growth** | | 14 | 1.02E-03 | -1.43 |  |
| GO:0042632 | **cholesterol homeostasis** | | 63 | 8.32E-06 | -0.94 |  |
| GO:0055072 |  | *iron ion homeostasis* | 21 | 5.08E-03 | -1.02 |  |
| GO:0055062 |  | *phosphate ion homeostasis* | 11 | 2.20E-03 | -1.50 |  |
| GO:0006879 |  | *cellular iron ion homeostasis* | 32 | 4.86E-03 | -0.83 |  |
| GO:2000189 |  | *positive regulation of cholesterol homeostasis* | 21 | 1.29E-05 | -1.54 |  |
| GO:0045454 |  | *cell redox homeostasis* | 87 | 2.21E-04 | -0.66 |  |
| GO:0016568 | **chromatin modification** | | 164 | 4.51E-07 | -0.66 |  |
| GO:0006337 |  | *nucleosome disassembly* | 10 | 4.87E-03 | -1.45 |  |
| GO:0006338 |  | *chromatin remodeling* | 76 | 5.70E-07 | -0.96 |  |
| GO:0006336 |  | *DNA replication-independent nucleosome assembly* | 15 | 1.70E-05 | -1.75 |  |
| GO:0006333 |  | *chromatin assembly or disassembly* | 6 | 4.41E-04 | -2.14 |  |
| GO:0006334 |  | *nucleosome assembly* | 62 | 5.47E-05 | -0.86 |  |
| GO:0006325 |  | *chromatin organization* | 22 | 8.72E-04 | -1.18 |  |
| GO:0007281 |  | *germ cell development* | 60 | 3.94E-03 | -0.62 |  |
| GO:0007283 |  | *spermatogenesis* | 275 | 3.25E-05 | -0.41 |  |
| GO:0000972 |  | *transcription-dependent tethering of RNA polymerase II gene DNA at nuclear periphery* | 7 | 2.76E-03 | -1.77 |  |
| GO:0033522 |  | *histone H2A ubiquitination* | 17 | 3.62E-03 | -1.17 |  |
| GO:0000910 |  | *cytokinesis* | 43 | 1.65E-03 | -0.80 |  |
| GO:0032465 |  | *regulation of cytokinesis* | 13 | 1.07E-04 | -1.70 |  |
| GO:0043044 |  | *ATP-dependent chromatin remodeling* | 19 | 1.39E-06 | -1.75 |  |
| GO:0048477 |  | *oogenesis* | 213 | 1.56E-04 | -0.42 |  |
| GO:0007126 |  | *meiotic nuclear division* | 36 | 4.68E-04 | -0.97 |  |
| GO:0043486 |  | *histone exchange* | 11 | 2.69E-03 | -1.47 |  |
| GO:0031065 |  | *positive regulation of histone deacetylation* | 8 | 2.24E-03 | -1.71 |  |
| GO:0007095 |  | *mitotic G2 DNA damage checkpoint* | 22 | 1.20E-03 | -1.15 |  |
| GO:0051569 |  | *regulation of histone H3-K4 methylation* | 8 | 6.09E-03 | -1.56 |  |
| GO:0007077 |  | *mitotic nuclear envelope disassembly* | 19 | 1.82E-04 | -1.40 |  |
| GO:0016584 |  | *nucleosome positioning* | 13 | 5.55E-03 | -1.27 |  |
| GO:0007062 |  | *sister chromatid cohesion* | 50 | 1.26E-03 | -0.76 |  |
| GO:0007067 |  | *mitotic nuclear division* | 284 | 7.08E-07 | -0.48 |  |
| GO:0043966 |  | *histone H3 acetylation* | 31 | 3.14E-03 | -0.89 |  |
| GO:0016571 |  | *histone methylation* | 27 | 1.56E-03 | -1.02 |  |
| GO:0007052 |  | *mitotic spindle organization* | 77 | 1.42E-03 | -0.60 |  |
| GO:0007030 |  | *Golgi organization* | 93 | 1.22E-03 | -0.55 |  |
| GO:0030707 |  | *ovarian follicle cell development* | 121 | 2.96E-03 | -0.44 |  |
| GO:0035093 |  | *spermatogenesis, exchange of chromosomal proteins* | 19 | 4.64E-06 | -1.67 |  |
| GO:0070050 |  | *neuron cellular homeostasis* | 24 | 2.64E-03 | -1.02 |  |
| GO:0042766 |  | *nucleosome mobilization* | 10 | 5.93E-03 | -1.42 |  |
| GO:0000723 |  | *telomere maintenance* | 24 | 7.60E-05 | -1.33 |  |
| GO:0000722 |  | *telomere maintenance via recombination* | 24 | 5.93E-03 | -0.94 |  |
| GO:0016032 | **viral process** | | 96 | 7.77E-06 | -0.76 |  |
| GO:0046718 |  | *viral entry into host cell* | 29 | 2.57E-04 | -1.13 |  |
| GO:0019083 |  | *viral transcription* | 16 | 1.42E-03 | -1.31 |  |
| GO:0019740 | **nitrogen utilization** | | 10 | 1.95E-03 | -1.58 |  |
| GO:0008283 | **cell proliferation** | | 201 | 3.14E-07 | -0.60 |  |
| GO:0097252 | **oligodendrocyte apoptotic process** | | 1 | 4.60E-03 | -4.62 |  |
| GO:0007275 | **multicellular organismal development** | | 489 | 9.76E-08 | -0.39 |  |
| GO:0046331 |  | *lateral inhibition* | 122 | 1.93E-03 | -0.46 |  |
| GO:0007399 |  | *nervous system development* | 261 | 3.39E-06 | -0.47 |  |
| GO:0007409 |  | *axonogenesis* | 123 | 1.27E-04 | -0.57 |  |
| GO:0050908 |  | *detection of light stimulus involved in visual perception* | 49 | 9.73E-05 | -0.93 |  |
| GO:0007412 |  | *axon target recognition* | 23 | 1.29E-04 | -1.31 |  |
| GO:0007411 |  | *axon guidance* | 253 | 2.03E-08 | -0.58 |  |
| GO:0060441 |  | *epithelial tube branching involved in lung morphogenesis* | 12 | 2.33E-03 | -1.43 |  |
| GO:0050885 |  | *neuromuscular process controlling balance* | 23 | 4.11E-03 | -1.00 |  |
| GO:0001942 |  | *hair follicle development* | 14 | 7.99E-04 | -1.46 |  |
| GO:0008045 |  | *motor neuron axon guidance* | 85 | 1.95E-04 | -0.67 |  |
| GO:0021680 |  | *cerebellar Purkinje cell layer development* | 10 | 3.93E-03 | -1.48 |  |
| GO:0035019 |  | *somatic stem cell maintenance* | 41 | 1.96E-03 | -0.81 |  |
| GO:0071907 |  | *determination of digestive tract left/right asymmetry* | 9 | 4.28E-03 | -1.54 |  |
| GO:0002532 |  | *production of molecular mediator involved in inflammatory response* | 7 | 4.37E-03 | -1.71 |  |
| GO:0061031 |  | *endodermal digestive tract morphogenesis* | 4 | 5.88E-03 | -2.07 |  |
| GO:0019233 |  | *sensory perception of pain* | 218 | 3.18E-06 | -0.52 |  |
| GO:0048598 |  | *embryonic morphogenesis* | 12 | 5.16E-04 | -1.61 |  |
| GO:0060119 |  | *inner ear receptor cell development* | 4 | 6.56E-05 | -2.73 |  |
| GO:0001503 |  | *ossification* | 42 | 5.09E-05 | -1.04 |  |
| GO:0050774 |  | *negative regulation of dendrite morphogenesis* | 17 | 9.80E-07 | -1.85 |  |
| GO:0045989 |  | *positive regulation of striated muscle contraction* | 3 | 1.57E-03 | -2.55 |  |
| GO:0050769 |  | *positive regulation of neurogenesis* | 18 | 5.18E-03 | -1.10 |  |
| GO:0001822 |  | *kidney development* | 78 | 2.80E-03 | -0.56 |  |
| GO:0022008 |  | *neurogenesis* | 216 | 1.56E-06 | -0.54 |  |
| GO:0042711 |  | *maternal behavior* | 5 | 1.84E-03 | -2.09 |  |
| GO:0045475 |  | *locomotor rhythm* | 37 | 3.68E-04 | -0.98 |  |
| GO:0060041 |  | *retina development in camera-type eye* | 29 | 1.24E-03 | -1.00 |  |
| GO:0010976 |  | *positive regulation of neuron projection development* | 85 | 4.33E-03 | -0.51 |  |
| GO:0007628 |  | *adult walking behavior* | 52 | 1.26E-05 | -1.01 |  |
| GO:0030574 |  | *collagen catabolic process* | 57 | 1.90E-03 | -0.68 |  |
| GO:0045638 |  | *negative regulation of myeloid cell differentiation* | 12 | 3.76E-03 | -1.37 |  |
| GO:0032504 |  | *multicellular organism reproduction* | 29 | 5.33E-05 | -1.24 |  |
| GO:0007605 |  | *sensory perception of sound* | 129 | 2.55E-04 | -0.53 |  |
| GO:0007608 |  | *sensory perception of smell* | 86 | 2.43E-03 | -0.54 |  |
| GO:0007612 |  | *learning* | 89 | 2.18E-04 | -0.65 |  |
| GO:0007613 |  | *memory* | 81 | 3.97E-05 | -0.76 |  |
| GO:0007614 |  | *short-term memory* | 35 | 1.37E-03 | -0.90 |  |
| GO:0007616 |  | *long-term memory* | 84 | 1.04E-03 | -0.59 |  |
| GO:0030097 |  | *hemopoiesis* | 63 | 2.86E-03 | -0.62 |  |
| GO:0021987 |  | *cerebral cortex development* | 41 | 8.98E-04 | -0.87 |  |
| GO:0048814 |  | *regulation of dendrite morphogenesis* | 37 | 7.09E-05 | -1.09 |  |
| GO:0048813 |  | *dendrite morphogenesis* | 242 | 5.73E-06 | -0.48 |  |
| GO:0048266 |  | *behavioral response to pain* | 22 | 5.01E-03 | -1.00 |  |
| GO:0007569 |  | *cell aging* | 31 | 2.46E-03 | -0.91 |  |
| GO:0007568 |  | *aging* | 82 | 9.23E-04 | -0.61 |  |
| GO:0048846 |  | *axon extension involved in axon guidance* | 27 | 2.40E-06 | -1.48 |  |
| GO:0030154 |  | *cell differentiation* | 293 | 1.27E-03 | -0.31 |  |
| GO:0048841 |  | *regulation of axon extension involved in axon guidance* | 18 | 2.89E-06 | -1.74 |  |
| GO:0007601 |  | *visual perception* | 204 | 2.79E-05 | -0.48 |  |
| GO:0045606 |  | *positive regulation of epidermal cell differentiation* | 4 | 3.52E-03 | -2.16 |  |
| GO:0009791 |  | *post-embryonic development* | 69 | 5.39E-03 | -0.55 |  |
| GO:0030182 |  | *neuron differentiation* | 62 | 2.01E-03 | -0.65 |  |
| GO:0008217 |  | *regulation of blood pressure* | 29 | 6.83E-05 | -1.22 |  |
| GO:0097105 |  | *presynaptic membrane assembly* | 18 | 2.30E-03 | -1.19 |  |
| GO:0060736 |  | *prostate gland growth* | 12 | 5.62E-03 | -1.32 |  |
| GO:0048149 |  | *behavioral response to ethanol* | 59 | 5.00E-03 | -0.61 |  |
| GO:0007507 |  | *heart development* | 194 | 4.97E-04 | -0.41 |  |
| GO:0007494 |  | *midgut development* | 30 | 7.59E-05 | -1.20 |  |
| GO:0035108 |  | *limb morphogenesis* | 20 | 5.66E-04 | -1.27 |  |
| GO:0007480 |  | *imaginal disc-derived leg morphogenesis* | 33 | 6.16E-03 | -0.80 |  |
| GO:0072553 |  | *terminal button organization* | 23 | 1.05E-03 | -1.14 |  |
| GO:0009887 |  | *organ morphogenesis* | 42 | 1.25E-03 | -0.83 |  |
| GO:0051124 |  | *synaptic growth at neuromuscular junction* | 43 | 6.55E-04 | -0.87 |  |
| GO:0007526 |  | *larval somatic muscle development* | 55 | 4.08E-03 | -0.64 |  |
| GO:0060325 |  | *face morphogenesis* | 25 | 1.01E-03 | -1.10 |  |
| GO:0042384 |  | *cilium assembly* | 134 | 4.52E-03 | -0.40 |  |
| GO:0001701 |  | *in utero embryonic development* | 157 | 6.15E-03 | -0.36 |  |
| GO:0048703 |  | *embryonic viscerocranium morphogenesis* | 31 | 4.52E-03 | -0.85 |  |
| GO:0001709 |  | *cell fate determination* | 47 | 2.26E-04 | -0.90 |  |
| GO:0072001 |  | *renal system development* | 12 | 3.04E-03 | -1.40 |  |
| GO:0007422 |  | *peripheral nervous system development* | 97 | 2.01E-05 | -0.72 |  |
| GO:0007424 |  | *open tracheal system development* | 112 | 1.15E-04 | -0.60 |  |
| GO:0007417 |  | *central nervous system development* | 164 | 3.20E-06 | -0.60 |  |
| GO:0007420 |  | *brain development* | 200 | 6.85E-04 | -0.39 |  |
| GO:0035158 |  | *regulation of tube diameter, open tracheal system* | 15 | 5.57E-03 | -1.19 |  |
| GO:0007413 |  | *axonal fasciculation* | 34 | 7.22E-05 | -1.13 |  |
| GO:0007476 |  | *imaginal disc-derived wing morphogenesis* | 172 | 6.74E-05 | -0.50 |  |
| GO:0008360 |  | *regulation of cell shape* | 145 | 6.90E-04 | -0.46 |  |
| GO:0008355 |  | *olfactory learning* | 64 | 4.66E-03 | -0.59 |  |
| GO:0008344 |  | *adult locomotory behavior* | 85 | 1.50E-05 | -0.78 |  |
| GO:0048749 |  | *compound eye development* | 114 | 1.62E-03 | -0.49 |  |
| GO:0045197 | **establishment or maintenance of epithelial cell apical/basal polarity** | | 26 | 4.25E-04 | -1.15 |  |
| GO:0001519 | **peptide amidation** | | 13 | 3.45E-05 | -1.80 |  |
| GO:1901687 | **glutathione derivative biosynthetic process** | | 5 | 4.86E-03 | -1.93 |  |
| GO:0005975 | **carbohydrate metabolic process** | | 131 | 1.41E-07 | -0.77 |  |
| GO:0009115 | **xanthine catabolic process** | | 23 | 7.20E-05 | -1.36 |  |
| GO:0008203 | **cholesterol metabolic process** | | 96 | 9.44E-07 | -0.84 |  |
| GO:0008206 |  | *bile acid metabolic process* | 24 | 2.54E-04 | -1.24 |  |
| GO:0007059 | **chromosome segregation** | | 56 | 4.99E-03 | -0.62 |  |
| GO:0009698 | **phenylpropanoid metabolic process** | | 14 | 1.58E-03 | -1.38 |  |
| GO:0006406 | **mRNA export from nucleus** | | 61 | 1.91E-09 | -1.27 |  |
| GO:0016256 |  | *N-glycan processing to lysosome* | 4 | 1.74E-03 | -2.28 |  |
| GO:0015031 |  | *protein transport* | 378 | 6.15E-06 | -0.38 |  |
| GO:0075733 |  | *intracellular transport of virus* | 21 | 1.30E-04 | -1.37 |  |
| GO:0006886 |  | *intracellular protein transport* | 248 | 1.47E-08 | -0.60 |  |
| GO:0034504 |  | *protein localization to nucleus* | 21 | 3.98E-04 | -1.28 |  |
| GO:0006606 |  | *protein import into nucleus* | 49 | 3.78E-08 | -1.30 |  |
| GO:0006607 |  | *NLS-bearing protein import into nucleus* | 17 | 2.75E-03 | -1.20 |  |
| GO:0051028 |  | *mRNA transport* | 50 | 7.85E-06 | -1.05 |  |
| GO:0000055 |  | *ribosomal large subunit export from nucleus* | 11 | 5.64E-03 | -1.37 |  |
| GO:0000056 |  | *ribosomal small subunit export from nucleus* | 6 | 3.05E-03 | -1.87 |  |
| GO:0007041 |  | *lysosomal transport* | 34 | 2.10E-03 | -0.88 |  |
| GO:0006409 |  | *tRNA export from nucleus* | 20 | 1.59E-03 | -1.17 |  |
| GO:0006405 |  | *RNA export from nucleus* | 28 | 9.67E-05 | -1.22 |  |
| GO:0090150 |  | *establishment of protein localization to membrane* | 19 | 3.11E-06 | -1.70 |  |
| GO:0006809 | **nitric oxide biosynthetic process** | | 26 | 2.65E-03 | -0.98 |  |
| GO:0051301 | **cell division** | | 352 | 7.47E-10 | -0.54 |  |
| GO:0007049 | **cell cycle** | | 191 | 6.61E-07 | -0.60 |  |
| GO:0010501 | **RNA secondary structure unwinding** | | 61 | 1.65E-03 | -0.67 |  |
| GO:0032091 | **negative regulation of protein binding** | | 22 | 1.24E-03 | -1.14 |  |
| GO:1904224 |  | *negative regulation of glucuronosyltransferase activity* | 2 | 5.19E-03 | -2.70 |  |
| GO:0006486 | **protein glycosylation** | | 203 | 6.79E-12 | -0.81 |  |
| GO:0036071 |  | *N-glycan fucosylation* | 23 | 3.64E-04 | -1.23 |  |
| GO:0044845 |  | *chain elongation of O-linked mannose residue* | 3 | 7.00E-05 | -3.05 |  |
| GO:0015012 |  | *heparan sulfate proteoglycan biosynthetic process* | 25 | 4.53E-03 | -0.95 |  |
| GO:0030206 |  | *chondroitin sulfate biosynthetic process* | 38 | 6.05E-03 | -0.74 |  |
| GO:0030259 |  | *lipid glycosylation* | 9 | 8.75E-05 | -1.99 |  |
| GO:0000032 |  | *cell wall mannoprotein biosynthetic process* | 4 | 1.14E-04 | -2.66 |  |
| GO:0006468 |  | *protein phosphorylation* | 329 | 2.28E-03 | -0.27 |  |
| GO:0006487 |  | *protein N-linked glycosylation* | 36 | 2.14E-04 | -1.03 |  |
| GO:0006491 |  | *N-glycan processing* | 24 | 8.01E-05 | -1.33 |  |
| GO:0006493 |  | *protein O-linked glycosylation* | 42 | 3.40E-04 | -0.92 |  |
| GO:0006897 | **endocytosis** | | 218 | 7.71E-07 | -0.55 |  |
| GO:0031338 |  | *regulation of vesicle fusion* | 41 | 2.58E-03 | -0.79 |  |
| GO:0016080 |  | *synaptic vesicle targeting* | 15 | 2.68E-03 | -1.28 |  |
| GO:0035526 |  | *retrograde transport, plasma membrane to Golgi* | 5 | 3.62E-03 | -1.98 |  |
| GO:0006898 |  | *receptor-mediated endocy*  *tosis* | 73 | 7.75E-04 | -0.65 |  |
| GO:0006890 |  | *retrograde vesicle-mediated transport, Golgi to ER* | 52 | 3.58E-04 | -0.83 |  |
| GO:0048489 |  | *synaptic vesicle transport* | 46 | 1.93E-03 | -0.76 |  |
| GO:0000398 | **mRNA splicing, via spliceosome** | | 183 | 2.16E-13 | -0.91 |  |
| GO:0000245 |  | *spliceosomal complex assembly* | 24 | 1.93E-03 | -1.05 |  |
| GO:0000956 |  | *nuclear-transcribed mRNA catabolic process* | 14 | 1.57E-04 | -1.62 |  |
| GO:0000381 |  | *regulation of alternative mRNA splicing, via spliceosome* | 89 | 8.26E-06 | -0.79 |  |
| GO:0000375 |  | *RNA splicing, via transesterification reactions* | 17 | 2.83E-03 | -1.20 |  |
| GO:0006397 |  | *mRNA processing* | 212 | 4.56E-09 | -0.67 |  |
| GO:0071044 |  | *histone mRNA catabolic process* | 14 | 3.48E-03 | -1.29 |  |
| GO:0000184 |  | *nuclear-transcribed mRNA catabolic process, nonsense-mediated decay* | 41 | 4.00E-03 | -0.75 |  |
| GO:0006914 | **autophagy** | | 138 | 1.51E-04 | -0.53 |  |
| GO:0045944 | **positive regulation of transcription from RNA polymerase II promoter** | | 601 | 2.25E-13 | -0.49 |  |
| GO:0006366 |  | *transcription from RNA polymerase II promoter* | 156 | 7.64E-06 | -0.59 |  |
| GO:0006367 |  | *transcription initiation from RNA polymerase II promoter* | 59 | 5.30E-04 | -0.75 |  |
| GO:0006357 |  | *regulation of transcription from RNA polymerase II promoter* | 343 | 1.98E-07 | -0.46 |  |
| GO:0045892 |  | *negative regulation of transcription, DNA-templated* | 453 | 5.54E-11 | -0.51 |  |
| GO:0010628 |  | *positive regulation of gene expression* | 159 | 2.50E-03 | -0.39 |  |
| GO:0006355 |  | *regulation of transcription, DNA-templated* | 1134 | 3.97E-15 | -0.38 |  |
| GO:0006351 |  | *transcription, DNA-templated* | 1855 | 8.12E-20 | -0.35 |  |
| GO:0045893 |  | *positive regulation of transcription, DNA-templated* | 406 | 1.50E-06 | -0.39 |  |
| GO:0006346 |  | *methylation-dependent chromatin silencing* | 4 | 5.91E-03 | -2.07 |  |
| GO:0006342 |  | *chromatin silencing* | 120 | 4.02E-05 | -0.62 |  |
| GO:0060548 |  | *negative regulation of cell death* | 47 | 1.88E-03 | -0.76 |  |
| GO:0043065 |  | *positive regulation of apoptotic process* | 163 | 1.73E-04 | -0.48 |  |
| GO:0070868 |  | *heterochromatin organization involved in chromatin silencing* | 5 | 2.41E-03 | -2.05 |  |
| GO:0010524 |  | *positive regulation of calcium ion transport into cytosol* | 18 | 1.24E-03 | -1.26 |  |
| GO:0031053 |  | *primary miRNA processing* | 16 | 5.44E-04 | -1.41 |  |
| GO:0035774 |  | *positive regulation of insulin secretion involved in cellular response to glucose stimulus* | 13 | 1.45E-03 | -1.44 |  |
| GO:0031585 |  | *regulation of inositol 1,4,5-trisphosphate-sensitive calcium-release channel activity* | 7 | 9.05E-04 | -1.93 |  |
| GO:0097202 |  | *activation of cysteine-type endopeptidase activity* | 6 | 1.91E-03 | -1.94 |  |
| GO:0000122 |  | *negative regulation of transcription from RNA polymerase II promoter* | 518 | 8.31E-10 | -0.44 |  |
| GO:0090197 |  | *positive regulation of chemokine secretion* | 6 | 3.13E-03 | -1.87 |  |
| GO:0010827 |  | *regulation of glucose transport* | 15 | 8.30E-04 | -1.41 |  |
| GO:0060316 |  | *positive regulation of ryanodine-sensitive calcium-release channel activity* | 5 | 5.85E-04 | -2.25 |  |
| GO:0032237 |  | *activation of store-operated calcium channel activity* | 6 | 3.18E-03 | -1.87 |  |
| GO:0090630 |  | *activation of GTPase activity* | 69 | 1.87E-03 | -0.62 |  |
| GO:0006915 |  | *apoptotic process* | 397 | 1.63E-03 | -0.26 |  |
| GO:0007220 | **Notch receptor processing** | | 13 | 5.38E-04 | -1.55 |  |
| GO:0031638 | **zymogen activation** | | 31 | 9.71E-07 | -1.44 |  |
| GO:0042127 | **regulation of cell proliferation** | | 116 | 1.67E-05 | -0.66 |  |
| GO:0061351 |  | *neural precursor cell proliferation* | 8 | 2.62E-03 | -1.69 |  |
| GO:0008284 |  | *positive regulation of cell proliferation* | 193 | 4.40E-05 | -0.48 |  |
| GO:0008285 |  | *negative regulation of cell proliferation* | 258 | 3.34E-04 | -0.36 |  |
| GO:0048134 |  | *germ-line cyst formation* | 7 | 4.38E-03 | -1.70 |  |
| GO:0016192 | **vesicle-mediated transport** | | 181 | 8.20E-05 | -0.48 |  |
| GO:0006817 | **phosphate ion transport** | | 22 | 1.33E-04 | -1.34 |  |
| GO:0000481 | **maturation of 5S rRNA** | | 1 | 4.65E-03 | -4.64 |  |
| GO:0043153 | **entrainment of circadian clock by photoperiod** | | 15 | 7.88E-04 | -1.42 |  |
| GO:1904380 | **endoplasmic reticulum mannose trimming** | | 7 | 2.54E-03 | -1.79 |  |
| GO:1904382 |  | *mannose trimming involved in glycoprotein ERAD pathway* | 6 | 3.50E-03 | -1.85 |  |
| GO:0019991 | **septate junction assembly** | | 56 | 1.42E-03 | -0.71 |  |
| GO:0045186 |  | *zonula adherens assembly* | 17 | 3.92E-03 | -1.16 |  |
| GO:0071404 | **cellular response to low-density lipoprotein particle stimulus** | | 24 | 3.23E-06 | -1.54 |  |
| GO:0006805 |  | *xenobiotic metabolic process* | 31 | 4.96E-03 | -0.84 |  |
| GO:0001975 |  | *response to amphetamine* | 14 | 2.84E-03 | -1.31 |  |
| GO:0071383 |  | *cellular response to steroid hormone stimulus* | 21 | 1.89E-05 | -1.51 |  |
| GO:0006490 | **oligosaccharide-lipid intermediate biosynthetic process** | | 4 | 1.94E-03 | -2.26 |  |
| GO:0000733 | **DNA strand renaturation** | | 6 | 4.09E-04 | -2.14 |  |
| GO:0031579 | **membrane raft organization** | | 24 | 3.34E-06 | -1.53 |  |
| GO:2000303 | **regulation of ceramide biosynthetic process** | | 3 | 6.84E-04 | -2.69 |  |
| GO:0045922 |  | *negative regulation of fatty acid metabolic process* | 2 | 5.19E-03 | -2.70 |  |
| GO:0022617 | **extracellular matrix disassembly** | | 34 | 7.16E-06 | -1.27 |  |
| GO:0043063 |  | *intercellular bridge organization* | 3 | 4.00E-03 | -2.38 |  |
| GO:0030198 |  | *extracellular matrix organization* | 103 | 5.79E-03 | -0.45 |  |
| GO:0085029 |  | *extracellular matrix assembly* | 4 | 5.88E-03 | -2.07 |  |
| GO:1901879 |  | *regulation of protein depolymerization* | 5 | 7.23E-04 | -2.22 |  |
| GO:0006517 | **protein deglycosylation** | | 17 | 5.54E-04 | -1.37 |  |
| GO:0006527 | **arginine catabolic process** | | 13 | 3.38E-04 | -1.60 |  |
| GO:0006396 | **RNA processing** | | 61 | 1.03E-03 | -0.70 |  |
| GO:0010468 |  | *regulation of gene expression* | 118 | 4.03E-04 | -0.54 |  |
| GO:0006429 | **leucyl-tRNA aminoacylation** | | 8 | 1.85E-03 | -1.74 |  |
| GO:0006508 | **proteolysis** | | 260 | 5.86E-09 | -0.60 |  |
| GO:0046856 | **phosphatidylinositol dephosphorylation** | | 27 | 2.62E-04 | -1.17 |  |
| GO:0006446 | **regulation of translational initiation** | | 36 | 2.60E-03 | -0.84 |  |
| GO:0017014 | **protein nitrosylation** | | 1 | 4.43E-03 | -3.98 |  |
| GO:0006099 | **tricarboxylic acid cycle** | | 75 | 5.16E-03 | -0.53 |  |
| GO:0042157 | **lipoprotein metabolic process** | | 25 | 2.93E-03 | -0.99 |  |
| GO:0050808 | **synapse organization** | | 50 | 1.04E-04 | -0.92 |  |
| GO:0033344 | **cholesterol efflux** | | 36 | 1.76E-08 | -1.52 |  |
| GO:0006869 |  | *lipid transport* | 63 | 3.13E-04 | -0.76 |  |
| GO:0032383 |  | *regulation of intracellular cholesterol transport* | 1 | 4.97E-03 | -4.73 |  |
| GO:0030301 |  | *cholesterol transport* | 24 | 5.52E-05 | -1.35 |  |
| GO:0052697 | **xenobiotic glucuronidation** | | 4 | 2.14E-03 | -2.24 |  |
| GO:2001030 |  | *negative regulation of cellular glucuronidation* | 2 | 5.19E-03 | -2.70 |  |
| GO:0000272 | **polysaccharide catabolic process** | | 65 | 3.37E-09 | -1.22 |  |
| GO:0006032 |  | *chitin catabolic process* | 62 | 2.44E-08 | -1.18 |  |
| GO:0006096 |  | *glycolytic process* | 62 | 3.73E-03 | -0.61 |  |
| GO:0016925 | **protein sumoylation** | | 72 | 2.60E-07 | -1.01 |  |
| GO:0016579 |  | *protein deubiquitination* | 53 | 5.94E-04 | -0.79 |  |
| GO:0071108 |  | *protein K48-linked deubiquitination* | 18 | 2.81E-03 | -1.17 |  |
| GO:0000209 |  | *protein polyubiquitination* | 117 | 6.05E-04 | -0.52 |  |
| GO:0031647 | **regulation of protein stability** | | 54 | 6.54E-04 | -0.77 |  |
| GO:0006810 | **transport** | | 218 | 6.67E-04 | -0.38 |  |
| GO:0055085 |  | *transmembrane transport* | 166 | 5.36E-03 | -0.35 |  |
| GO:0000052 | **citrulline metabolic process** | | 6 | 2.79E-03 | -1.89 |  |
| GO:0042264 | **peptidyl-aspartic acid hydroxylation** | | 5 | 7.23E-04 | -2.22 |  |
| GO:0006013 | **mannose metabolic process** | | 24 | 9.68E-05 | -1.31 |  |
| GO:0006004 |  | *fucose metabolic process* | 13 | 5.31E-03 | -1.28 |  |
| GO:0010499 | **proteasomal ubiquitin-independent protein catabolic process** | | 21 | 1.94E-05 | -1.51 |  |
| GO:0030433 |  | *ER-associated ubiquitin-dependent protein catabolic process* | 57 | 3.50E-05 | -0.92 |  |
| GO:1900101 |  | *regulation of endoplasmic reticulum unfolded protein response* | 6 | 3.18E-03 | -1.87 |  |
| GO:0042176 |  | *regulation of protein catabolic process* | 19 | 9.15E-04 | -1.26 |  |
| GO:0051603 |  | *proteolysis involved in cellular protein catabolic process* | 60 | 1.52E-04 | -0.82 |  |
| GO:0006511 |  | *ubiquitin-dependent protein catabolic process* | 138 | 2.51E-05 | -0.59 |  |
| GO:0042787 |  | *protein ubiquitination involved in ubiquitin-dependent protein catabolic process* | 185 | 2.76E-03 | -0.36 |  |
| GO:0019853 | **L-ascorbic acid biosynthetic process** | | 8 | 1.44E-04 | -2.03 |  |
| GO:0043171 | **peptide catabolic process** | | 39 | 7.79E-04 | -0.90 |  |
| GO:0006518 |  | *peptide metabolic process* | 42 | 2.71E-03 | -0.77 |  |
| GO:0007269 | **neurotransmitter secretion** | | 104 | 3.19E-03 | -0.48 |  |
| GO:0042493 | **response to drug** | | 250 | 5.75E-06 | -0.47 |  |
| GO:0072488 | **ammonium transmembrane transport** | | 10 | 6.14E-03 | -1.41 |  |
| GO:0016055 | **Wnt signaling pathway** | | 138 | 4.05E-06 | -0.65 |  |
| GO:0042058 |  | *regulation of epidermal growth factor receptor signaling pathway* | 22 | 2.00E-03 | -1.10 |  |
| GO:0007224 |  | *smoothened signaling pathway* | 78 | 3.16E-03 | -0.55 |  |
| GO:0008063 |  | *Toll signaling pathway* | 48 | 1.10E-05 | -1.06 |  |
| GO:0007219 |  | *Notch signaling pathway* | 139 | 2.56E-03 | -0.42 |  |
| GO:0007169 |  | *transmembrane receptor protein tyrosine kinase signaling pathway* | 79 | 5.12E-03 | -0.52 |  |
| GO:0007173 |  | *epidermal growth factor receptor signaling pathway* | 50 | 3.91E-03 | -0.68 |  |
| GO:0036323 |  | *vascular endothelial growth factor receptor-1 signaling pathway* | 2 | 3.60E-03 | -2.79 |  |
| GO:0006457 | **protein folding** | | 172 | 1.20E-07 | -0.67 |  |
| GO:0016242 | **negative regulation of macroautophagy** | | 34 | 3.28E-06 | -1.31 |  |
| GO:1900745 |  | *positive regulation of p38MAPK cascade* | 11 | 3.18E-03 | -1.45 |  |
| GO:1900034 |  | *regulation of cellular response to heat* | 25 | 6.11E-05 | -1.32 |  |
| GO:0007584 |  | *response to nutrient* | 98 | 5.65E-03 | -0.46 |  |
| GO:0007311 | **maternal specification of dorsal/ventral axis, oocyte, germ-line encoded** | | 23 | 5.20E-07 | -1.67 |  |
| GO:0007367 |  | *segment polarity determination* | 44 | 4.06E-04 | -0.89 |  |
| GO:0007379 |  | *segment specification* | 33 | 6.30E-06 | -1.30 |  |
| GO:0007295 |  | *growth of a germarium-derived egg chamber* | 8 | 1.59E-03 | -1.76 |  |
| GO:0007279 |  | *pole cell formation* | 28 | 9.71E-04 | -1.04 |  |
| GO:0007620 |  | *copulation* | 37 | 3.46E-03 | -0.80 |  |
| GO:0007530 |  | *sex determination* | 31 | 5.84E-03 | -0.83 |  |
| GO:0009950 |  | *dorsal/ventral axis specification* | 56 | 1.94E-04 | -0.83 |  |
| GO:0009954 |  | *proximal/distal pattern formation* | 10 | 2.84E-03 | -1.53 |  |
| GO:0009953 |  | *dorsal/ventral pattern formation* | 65 | 8.04E-04 | -0.69 |  |
| GO:0009301 | **snRNA transcription** | | 2 | 1.40E-04 | -3.60 |  |
| GO:0008380 | **RNA splicing** | | 161 | 4.31E-11 | -0.87 |  |
| GO:0006364 |  | *rRNA processing* | 101 | 3.07E-03 | -0.48 |  |
| GO:0002548 | **monocyte chemotaxis** | | 2 | 3.60E-03 | -2.79 |  |
| GO:0010632 |  | *regulation of epithelial cell migration* | 30 | 4.35E-03 | -0.87 |  |
| GO:0061580 |  | *colon epithelial cell migration* | 22 | 5.33E-03 | -0.99 |  |
| GO:0040017 |  | *positive regulation of locomotion* | 20 | 5.76E-03 | -1.03 |  |
| GO:0006270 | **DNA replication initiation** | | 36 | 3.92E-03 | -0.80 |  |
| GO:0060052 | **neurofilament cytoskeleton organization** | | 10 | 4.54E-03 | -1.46 |  |
| GO:0034316 |  | *negative regulation of Arp2/3 complex-mediated actin nucleation* | 9 | 6.03E-03 | -1.49 |  |
| GO:0030866 |  | *cortical actin cytoskeleton organization* | 32 | 5.82E-03 | -0.81 |  |
| GO:0045467 | **R7 cell development** | | 67 | 5.41E-07 | -1.02 |  |
| GO:0016318 |  | *ommatidial rotation* | 41 | 1.10E-05 | -1.14 |  |
| GO:0046548 |  | *retinal rod cell development* | 4 | 2.77E-04 | -2.54 |  |
| GO:0045463 |  | *R8 cell development* | 18 | 6.52E-06 | -1.69 |  |
| GO:0060042 |  | *retina morphogenesis in camera-type eye* | 14 | 5.83E-04 | -1.49 |  |
| GO:0046667 |  | *compound eye retinal cell programmed cell death* | 39 | 4.07E-03 | -0.77 |  |
| GO:0022416 |  | *chaeta development* | 47 | 5.31E-04 | -0.84 |  |
| GO:0019985 | **translesion synthesis** | | 12 | 2.94E-03 | -1.40 |  |
| GO:0008090 | **retrograde axon cargo transport** | | 7 | 5.17E-03 | -1.68 |  |
| GO:0006978 | **DNA damage response, signal transduction by p53 class mediator resulting in transcription of p21 class mediator** | | 6 | 1.43E-03 | -1.98 |  |
| GO:0010906 | **regulation of glucose metabolic process** | | 138 | 4.17E-03 | -0.40 |  |
| GO:0046686 | **response to cadmium ion** | | 55 | 7.55E-06 | -1.01 |  |

| **Supplementary Table 3.** Molecular function GO terms significantly associated with down-regulation in lowered pH, as evaluated by Babelomics’ logistic regression. As in ST2. | | | | | |
| --- | --- | --- | --- | --- | --- |
| GO ID | GO Description | | # contigs | Adj *p-*value | LOR |
| GO:0003700 | **sequence-specific DNA binding transcription factor activity** | | 807 | 3.15E-04 | -0.20 |
| GO:0000981 | **sequence-specific DNA binding RNA polymerase II transcription factor activity** | | 109 | 3.06E-02 | -0.34 |
| GO:0003713 | **transcription coactivator activity** | | 132 | 5.65E-05 | -0.58 |
| GO:0000989 |  | *transcription factor binding transcription factor activity* | 11 | 4.01E-02 | -1.03 |
| GO:0001104 |  | *RNA polymerase II transcription cofactor activity* | 34 | 2.93E-03 | -0.85 |
| GO:0003824 | **catalytic activity** | | 70 | 1.52E-03 | -0.63 |
| GO:0004867 | **serine-type endopeptidase inhibitor activity** | | 88 | 5.99E-12 | -1.22 |
| GO:0008047 |  | *enzyme activator activity* | 28 | 1.06E-03 | -1.03 |
| GO:0008607 |  | *phosphorylase kinase regulator activity* | 17 | 1.27E-02 | -1.01 |
| GO:0019887 |  | *protein kinase regulator activity* | 20 | 1.34E-03 | -1.19 |
| GO:0016504 |  | *peptidase activator activity* | 30 | 8.09E-05 | -1.19 |
| GO:0005096 |  | *GTPase activator activity* | 231 | 2.78E-05 | -0.45 |
| GO:0004872 | **receptor activity** | | 179 | 5.34E-11 | -0.82 |
| GO:0004709 |  | *MAP kinase activity* | 24 | 1.11E-02 | -0.87 |
| GO:0004714 |  | *transmembrane receptor protein tyrosine kinase activity* | 54 | 2.70E-03 | -0.68 |
| GO:0004702 |  | *receptor signaling protein serine/threonine kinase activity* | 33 | 4.74E-02 | -0.57 |
| GO:0008158 |  | *hedgehog receptor activity* | 8 | 4.22E-02 | -1.19 |
| GO:0036327 |  | *VEGF-B-activated receptor activity* | 2 | 3.60E-03 | -2.79 |
| GO:0036326 |  | *VEGF-A-activated receptor activity* | 2 | 3.60E-03 | -2.79 |
| GO:0036332 |  | *placental growth factor-activated receptor activity* | 2 | 3.60E-03 | -2.79 |
| GO:0004888 |  | *transmembrane signaling receptor activity* | 60 | 1.62E-04 | -0.81 |
| GO:0005198 | **structural molecule activity** | | 94 | 3.47E-03 | -0.50 |
| GO:0005215 | **transporter activity** | | 129 | 1.65E-02 | -0.34 |
| GO:0008233 | **peptidase activity** | | 82 | 1.10E-09 | -1.12 |
| GO:0008565 | **protein transporter activity** | | 66 | 1.80E-06 | -0.98 |
| GO:0009055 | **electron carrier activity** | | 153 | 1.85E-05 | -0.57 |
| GO:0016015 | **morphogen activity** | | 18 | 2.71E-02 | -0.87 |
| GO:0017056 | **structural constituent of nuclear pore** | | 23 | 1.57E-06 | -1.61 |
| GO:0044822 | **poly(A) RNA binding** | | 678 | 1.08E-19 | -0.58 |
| GO:0019172 | **glyoxalase III activity** | | 2 | 1.47E-02 | -2.44 |
| GO:0034647 | **histone demethylase activity (H3-trimethyl-K4 specific)** | | 13 | 7.57E-03 | -1.23 |
| GO:0051747 |  | *cytosine C-5 DNA demethylase activity* | 2 | 1.35E-02 | -2.47 |
| GO:0051864 |  | *histone demethylase activity (H3-K36 specific)* | 23 | 2.29E-02 | -0.79 |
| GO:0016207 | **4-coumarate-CoA ligase activity** | | 14 | 1.58E-03 | -1.38 |
| GO:0031957 |  | *very long-chain fatty acid-CoA ligase activity* | 33 | 4.57E-02 | -0.58 |
| GO:0004467 |  | *long-chain fatty acid-CoA ligase activity* | 80 | 4.77E-03 | -0.52 |
| GO:0004169 | **dolichyl-phosphate-mannose-protein mannosyltransferase activity** | | 9 | 2.55E-07 | -2.45 |
| GO:0016763 |  | *transferase activity, transferring pentosyl groups* | 20 | 1.78E-04 | -1.37 |
| GO:0000030 |  | *mannosyltransferase activity* | 11 | 6.71E-03 | -1.34 |
| GO:0008417 |  | *fucosyltransferase activity* | 45 | 6.67E-04 | -0.85 |
| GO:0004579 |  | *dolichyl-diphosphooligosaccharide-protein glycotransferase activity* | 6 | 2.54E-02 | -1.48 |
| GO:0016702 | **oxidoreductase activity, acting on single donors with incorporation of molecular oxygen, incorporation of two atoms of oxygen** | | 51 | 1.36E-06 | -1.13 |
| GO:0043734 |  | *DNA-N1-methyladenine dioxygenase activity* | 2 | 1.35E-02 | -2.47 |
| GO:0004597 |  | *peptide-aspartate beta-dioxygenase activity* | 5 | 7.23E-04 | -2.22 |
| GO:0004656 |  | *procollagen-proline 4-dioxygenase activity* | 35 | 2.33E-05 | -1.19 |
| GO:0008475 |  | *procollagen-lysine 5-dioxygenase activity* | 4 | 1.62E-02 | -1.86 |
| GO:0016708 |  | *oxidoreductase activity, acting on paired donors, with incorporation or reduction of molecular oxygen, NAD(P)H as one donor, and incorporation of two atoms of oxygen into one donor* | 1 | 9.25E-03 | -3.43 |
| GO:0031545 |  | *peptidyl-proline 4-dioxygenase activity* | 8 | 4.73E-02 | -1.16 |
| GO:0018576 |  | *catechol 1,2-dioxygenase activity* | 1 | 3.47E-02 | -2.84 |
| GO:0018581 |  | *hydroxyquinol 1,2-dioxygenase activity* | 1 | 3.47E-02 | -2.84 |
| GO:0003755 | **peptidyl-prolyl cis-trans isomerase activity** | | 61 | 3.05E-06 | -1.00 |
| GO:0070577 | **lysine-acetylated histone binding** | | 30 | 2.02E-07 | -1.54 |
| GO:0035064 |  | *methylated histone binding* | 47 | 1.66E-02 | -0.58 |
| GO:0016874 | **ligase activity** | | 342 | 1.83E-04 | -0.33 |
| GO:0008061 | **chitin binding** | | 66 | 1.38E-04 | -0.78 |
| GO:0016829 | **lyase activity** | | 24 | 7.52E-03 | -0.91 |
| GO:0042277 | **peptide binding** | | 65 | 2.64E-04 | -0.75 |
| GO:0008139 |  | *nuclear localization sequence binding* | 21 | 8.44E-04 | -1.21 |
| GO:0043295 |  | *glutathione binding* | 10 | 1.25E-02 | -1.30 |
| GO:0003682 | **chromatin binding** | | 377 | 3.73E-12 | -0.59 |
| GO:1990841 |  | *promoter-specific chromatin binding* | 20 | 1.49E-02 | -0.91 |
| GO:0016491 | **oxidoreductase activity** | | 190 | 5.76E-06 | -0.54 |
| GO:0008270 | **zinc ion binding** | | 1482 | 1.40E-18 | -0.38 |
| GO:0046872 |  | *metal ion binding* | 2666 | 8.10E-15 | -0.25 |
| GO:0030151 |  | *molybdenum ion binding* | 21 | 4.85E-03 | -1.03 |
| GO:0005506 |  | *iron ion binding* | 254 | 4.97E-05 | -0.42 |
| GO:0005507 |  | *copper ion binding* | 63 | 2.22E-03 | -0.64 |
| GO:0008289 | **lipid binding** | | 111 | 6.76E-04 | -0.53 |
| GO:0005528 | **FK506 binding** | | 26 | 3.02E-03 | -0.97 |
| GO:0008144 | **drug binding** | | 47 | 1.77E-02 | -0.57 |
| GO:0051537 | **2 iron, 2 sulfur cluster binding** | | 51 | 1.16E-05 | -1.03 |
| GO:0051539 |  | *4 iron, 4 sulfur cluster binding* | 57 | 2.95E-03 | -0.65 |
| GO:0030246 | **carbohydrate binding** | | 229 | 6.68E-10 | -0.68 |
| GO:0016787 | **hydrolase activity** | | 146 | 9.14E-03 | -0.35 |
| GO:0031404 | **chloride ion binding** | | 11 | 1.50E-02 | -1.21 |
| GO:0046976 | **histone methyltransferase activity (H3-K27 specific)** | | 9 | 6.76E-03 | -1.47 |
| GO:0046974 |  | *histone methyltransferase activity (H3-K9 specific)* | 8 | 1.59E-02 | -1.39 |
| GO:0042800 |  | *histone methyltransferase activity (H3-K4 specific)* | 27 | 1.84E-02 | -0.76 |
| GO:0061599 | **molybdopterin molybdotransferase activity** | | 4 | 1.25E-02 | -1.92 |
| GO:0003676 | **nucleic acid binding** | | 161 | 1.20E-04 | -0.50 |
| GO:0004364 | **glutathione transferase activity** | | 50 | 6.59E-07 | -1.17 |
| GO:0004766 |  | *spermidine synthase activity* | 1 | 1.91E-02 | -3.09 |
| GO:0031418 | **L-ascorbic acid binding** | | 57 | 2.56E-06 | -1.04 |
| GO:0004402 | **histone acetyltransferase activity** | | 27 | 3.30E-04 | -1.15 |
| GO:0015485 | **cholesterol binding** | | 71 | 3.26E-06 | -0.92 |
| GO:0032266 |  | *phosphatidylinositol-3-phosphate binding* | 26 | 2.34E-02 | -0.74 |
| GO:0035091 |  | *phosphatidylinositol binding* | 86 | 3.11E-02 | -0.38 |
| GO:0008429 |  | *phosphatidylethanolamine binding* | 12 | 2.27E-02 | -1.09 |
| GO:0031210 |  | *phosphatidylcholine binding* | 12 | 3.82E-02 | -1.00 |
| GO:0005504 |  | *fatty acid binding* | 12 | 4.11E-02 | -0.98 |
| GO:0005543 |  | *phospholipid binding* | 40 | 1.09E-02 | -0.67 |
| GO:0070615 | **nucleosome-dependent ATPase activity** | | 4 | 5.01E-03 | -2.10 |
| GO:0042301 | **phosphate ion binding** | | 14 | 4.56E-03 | -1.25 |
| GO:0016403 | **dimethylargininase activity** | | 4 | 1.31E-03 | -2.32 |
| GO:0004301 | **epoxide hydrolase activity** | | 11 | 1.61E-03 | -1.54 |
| GO:0035671 | **enone reductase activity** | | 3 | 3.29E-02 | -1.90 |
| GO:0047734 | **CDP-glycerol diphosphatase activity** | | 2 | 2.83E-02 | -2.26 |
| GO:0004031 | **aldehyde oxidase activity** | | 8 | 3.79E-02 | -1.22 |
| GO:0008201 | **heparin binding** | | 81 | 2.38E-03 | -0.56 |
| GO:0000062 |  | *fatty-acyl-CoA binding* | 64 | 1.17E-02 | -0.52 |
| GO:0004674 | **protein serine/threonine kinase activity** | | 390 | 2.56E-03 | -0.25 |
| GO:0004672 |  | *protein kinase activity* | 189 | 2.33E-02 | -0.27 |
| GO:0004504 | **peptidylglycine monooxygenase activity** | | 14 | 2.88E-05 | -1.76 |
| GO:0008146 | **sulfotransferase activity** | | 46 | 3.72E-02 | -0.51 |
| GO:0004842 | **ubiquitin-protein transferase activity** | | 283 | 3.97E-03 | -0.28 |
| GO:0019789 |  | *SUMO transferase activity* | 18 | 6.44E-03 | -1.07 |
| GO:0061630 |  | *ubiquitin protein ligase activity* | 185 | 3.32E-02 | -0.25 |
| GO:0008131 | **primary amine oxidase activity** | | 1 | 2.73E-02 | -2.94 |
| GO:0050660 | **flavin adenine dinucleotide binding** | | 208 | 1.79E-04 | -0.43 |
| GO:0010181 |  | *FMN binding* | 36 | 9.25E-04 | -0.92 |
| GO:0050661 |  | *NADP binding* | 80 | 1.87E-02 | -0.43 |
| GO:0030170 |  | *pyridoxal phosphate binding* | 103 | 8.02E-03 | -0.43 |
| GO:0070402 |  | *NADPH binding* | 12 | 3.08E-02 | -1.04 |
| GO:0004854 | **xanthine dehydrogenase activity** | | 24 | 6.84E-05 | -1.34 |
| GO:0070674 |  | *hypoxanthine dehydrogenase activity* | 5 | 3.83E-03 | -1.97 |
| GO:0004748 |  | *ribonucleoside-diphosphate reductase activity, thioredoxin disulfide as acceptor* | 9 | 3.57E-02 | -1.16 |
| GO:0004855 |  | *xanthine oxidase activity* | 17 | 2.50E-04 | -1.45 |
| GO:0051903 | **S-(hydroxymethyl)glutathione dehydrogenase activity** | | 4 | 1.70E-02 | -1.85 |
| GO:0004598 | **peptidylamidoglycolate lyase activity** | | 7 | 4.62E-02 | -1.25 |
| GO:0004568 | **chitinase activity** | | 53 | 2.63E-08 | -1.26 |
| GO:0033932 |  | *1,3-alpha-L-fucosidase activity* | 16 | 1.10E-04 | -1.56 |
| GO:0004557 |  | *alpha-galactosidase activity* | 5 | 1.50E-02 | -1.72 |
| GO:0004560 |  | *alpha-L-fucosidase activity* | 4 | 1.85E-04 | -2.60 |
| GO:0004559 |  | *alpha-mannosidase activity* | 18 | 1.77E-05 | -1.62 |
| GO:0004556 |  | *alpha-amylase activity* | 5 | 2.20E-03 | -2.06 |
| GO:0004565 |  | *beta-galactosidase activity* | 6 | 3.37E-03 | -1.86 |
| GO:0004571 |  | *mannosyl-oligosaccharide 1,2-alpha-mannosidase activity* | 9 | 5.55E-04 | -1.80 |
| GO:0005487 | **nucleocytoplasmic transporter activity** | | 22 | 4.76E-06 | -1.57 |
| GO:0043565 | **sequence-specific DNA binding** | | 455 | 3.52E-05 | -0.32 |
| GO:0003976 | **UDP-N-acetylglucosamine-lysosomal-enzyme N-acetylglucosaminephosphotransferase activity** | | 6 | 3.33E-03 | -1.86 |
| GO:0061598 | **molybdopterin adenylyltransferase activity** | | 4 | 1.25E-02 | -1.92 |
| GO:0004781 |  | *sulfate adenylyltransferase (ATP) activity* | 8 | 2.88E-02 | -1.28 |
| GO:0034437 | **glycoprotein transporter activity** | | 5 | 2.03E-02 | -1.65 |
| GO:0003678 | **DNA helicase activity** | | 37 | 6.38E-07 | -1.35 |
| GO:0043140 |  | *ATP-dependent 3'-5' DNA helicase activity* | 14 | 2.11E-03 | -1.35 |
| GO:0004004 |  | *ATP-dependent RNA helicase activity* | 90 | 2.33E-04 | -0.64 |
| GO:0009378 |  | *four-way junction helicase activity* | 12 | 6.50E-03 | -1.29 |
| GO:0031402 | **sodium ion binding** | | 20 | 3.91E-03 | -1.07 |
| GO:0004222 | **metalloendopeptidase activity** | | 222 | 1.08E-05 | -0.49 |
| GO:0016805 |  | *dipeptidase activity* | 15 | 8.85E-03 | -1.12 |
| GO:0004197 |  | *cysteine-type endopeptidase activity* | 81 | 1.15E-04 | -0.71 |
| GO:0004185 |  | *serine-type carboxypeptidase activity* | 48 | 3.34E-04 | -0.86 |
| GO:0004190 |  | *aspartic-type endopeptidase activity* | 8 | 6.27E-03 | -1.56 |
| GO:0004180 |  | *carboxypeptidase activity* | 48 | 1.66E-02 | -0.57 |
| GO:0004177 |  | *aminopeptidase activity* | 56 | 3.97E-04 | -0.79 |
| GO:0004175 |  | *endopeptidase activity* | 41 | 5.52E-04 | -0.90 |
| GO:0004252 |  | *serine-type endopeptidase activity* | 360 | 4.83E-05 | -0.35 |
| GO:0004298 |  | *threonine-type endopeptidase activity* | 16 | 2.04E-02 | -0.97 |
| GO:0004843 |  | *ubiquitin-specific protease activity* | 59 | 1.09E-03 | -0.71 |
| GO:0043130 |  | *ubiquitin binding* | 52 | 4.83E-02 | -0.45 |
| GO:0008798 |  | *beta-aspartyl-peptidase activity* | 4 | 6.22E-03 | -2.06 |
| GO:0008236 |  | *serine-type peptidase activity* | 61 | 4.93E-03 | -0.60 |
| GO:0008234 |  | *cysteine-type peptidase activity* | 34 | 9.73E-03 | -0.74 |
| GO:0008239 |  | *dipeptidyl-peptidase activity* | 22 | 6.18E-03 | -0.97 |
| GO:0008238 |  | *exopeptidase activity* | 11 | 3.45E-02 | -1.06 |
| GO:0008237 |  | *metallopeptidase activity* | 115 | 2.45E-04 | -0.57 |
| GO:0070006 |  | *metalloaminopeptidase activity* | 39 | 2.79E-02 | -0.58 |
| GO:0052689 | **carboxylic ester hydrolase activity** | | 68 | 3.00E-07 | -1.04 |
| GO:0097157 | **pre-mRNA intronic binding** | | 7 | 3.89E-02 | -1.29 |
| GO:0004332 | **fructose-bisphosphate aldolase activity** | | 4 | 1.56E-02 | -1.87 |
| GO:0016903 | **oxidoreductase activity, acting on the aldehyde or oxo group of donors** | | 17 | 2.40E-04 | -1.45 |
| GO:0000976 | **transcription regulatory region sequence-specific DNA binding** | | 43 | 4.64E-04 | -0.89 |
| GO:0044212 |  | *transcription regulatory region DNA binding* | 126 | 8.45E-03 | -0.38 |
| GO:0000977 |  | *RNA polymerase II regulatory region sequence-specific DNA binding* | 149 | 9.49E-03 | -0.35 |
| GO:0000978 |  | *RNA polymerase II core promoter proximal region sequence-specific DNA binding* | 197 | 2.67E-03 | -0.35 |
| GO:0000980 |  | *RNA polymerase II distal enhancer sequence-specific DNA binding* | 45 | 2.85E-03 | -0.74 |
| GO:0000975 |  | *regulatory region DNA binding* | 2 | 2.73E-02 | -2.27 |
| GO:0035326 |  | *enhancer binding* | 4 | 1.88E-02 | -1.83 |
| GO:0031711 | **bradykinin receptor binding** | | 2 | 2.45E-02 | -2.30 |
| GO:0015248 | **sterol transporter activity** | | 12 | 1.16E-04 | -1.75 |
| GO:0004012 |  | *phospholipid-translocating ATPase activity* | 37 | 1.61E-02 | -0.66 |
| GO:0005548 |  | *phospholipid transporter activity* | 10 | 2.93E-02 | -1.14 |
| GO:0097108 | **hedgehog family protein binding** | | 5 | 4.50E-03 | -1.95 |
| GO:0017064 | **fatty acid amide hydrolase activity** | | 2 | 1.09E-02 | -2.52 |
| GO:0030794 | **(S)-coclaurine-N-methyltransferase activity** | | 1 | 1.18E-02 | -3.31 |
| GO:0070052 | **collagen V binding** | | 1 | 1.67E-02 | -3.15 |
| GO:0036033 |  | *mediator complex binding* | 5 | 2.05E-02 | -1.65 |
| GO:0010997 |  | *anaphase-promoting complex binding* | 3 | 2.79E-02 | -1.94 |
| GO:0030957 | **Tat protein binding** | | 1 | 1.26E-02 | -3.28 |
| GO:0001085 |  | *RNA polymerase II transcription factor binding* | 40 | 4.14E-02 | -0.53 |
| GO:0070491 |  | *repressing transcription factor binding* | 38 | 1.30E-02 | -0.67 |
| GO:0003689 | **DNA clamp loader activity** | | 7 | 4.35E-02 | -1.26 |
| GO:0034185 | **apolipoprotein binding** | | 18 | 1.34E-02 | -0.97 |
| GO:0016772 | **transferase activity, transferring phosphorus-containing groups** | | 1 | 4.24E-02 | -2.75 |
| GO:0015216 | **purine nucleotide transmembrane transporter activity** | | 1 | 1.14E-02 | -3.33 |
| GO:0070410 | **co-SMAD binding** | | 5 | 4.50E-02 | -1.46 |
| GO:0032183 | **SUMO binding** | | 13 | 2.68E-02 | -1.02 |
| GO:0071889 | **14-3-3 protein binding** | | 17 | 2.91E-02 | -0.88 |
| GO:0050431 | **transforming growth factor beta binding** | | 18 | 1.81E-02 | -0.93 |
| GO:0051139 | **metal ion:proton antiporter activity** | | 2 | 6.23E-03 | -2.66 |
| GO:0015293 |  | *symporter activity* | 147 | 2.32E-02 | -0.30 |
| GO:0004823 | **leucine-tRNA ligase activity** | | 8 | 1.85E-03 | -1.74 |
| GO:0004821 |  | *histidine-tRNA ligase activity* | 3 | 2.64E-02 | -1.96 |
| GO:0001681 | **sialate O-acetylesterase activity** | | 12 | 1.07E-03 | -1.53 |
| GO:0004104 |  | *cholinesterase activity* | 6 | 7.64E-03 | -1.72 |
| GO:0004341 |  | *gluconolactonase activity* | 1 | 7.42E-03 | -3.55 |
| GO:0003990 |  | *acetylcholinesterase activity* | 19 | 1.41E-02 | -0.94 |
| GO:0002161 |  | *aminoacyl-tRNA editing activity* | 21 | 3.89E-03 | -1.05 |
| GO:0008420 | **CTD phosphatase activity** | | 2 | 4.28E-04 | -3.28 |
| GO:0051213 | **dioxygenase activity** | | 38 | 8.40E-04 | -0.90 |
| GO:0005201 | **extracellular matrix structural constituent** | | 54 | 1.88E-03 | -0.70 |
| GO:0030234 | **enzyme regulator activity** | | 10 | 1.83E-05 | -2.05 |
| GO:0042768 | **ecdysteroid 2-hydroxylase activity** | | 2 | 2.44E-03 | -2.88 |
| GO:0004501 |  | *ecdysone 20-monooxygenase activity* | 1 | 3.02E-02 | -2.90 |
| GO:0016810 | **hydrolase activity, acting on carbon-nitrogen (but not peptide) bonds** | | 7 | 1.66E-02 | -1.47 |
| GO:0033885 | **10-hydroxy-9-(phosphonooxy)octadecanoate phosphatase activity** | | 2 | 1.39E-02 | -2.46 |
| GO:0003697 | **single-stranded DNA binding** | | 70 | 6.32E-03 | -0.54 |
| GO:0004534 | **5'-3' exoribonuclease activity** | | 4 | 1.07E-02 | -1.95 |
| GO:0000175 |  | *3'-5'-exoribonuclease activity* | 18 | 3.12E-02 | -0.85 |
| GO:0008408 |  | *3'-5' exonuclease activity* | 26 | 4.62E-02 | -0.65 |
| GO:0004667 | **prostaglandin-D synthase activity** | | 3 | 2.06E-04 | -2.88 |
| GO:0003684 | **damaged DNA binding** | | 56 | 1.19E-02 | -0.56 |
| GO:0005509 | **calcium ion binding** | | 1042 | 1.31E-05 | -0.22 |
| GO:0046029 | **mannitol dehydrogenase activity** | | 1 | 3.66E-02 | -2.82 |
| GO:0030339 | **fatty-acyl-ethyl-ester synthase activity** | | 1 | 3.36E-02 | -2.85 |
| GO:0008240 | **tripeptidyl-peptidase activity** | | 3 | 1.33E-02 | -2.13 |
| GO:0034417 | **bisphosphoglycerate 3-phosphatase activity** | | 4 | 3.49E-02 | -1.67 |
| GO:0015321 | **sodium-dependent phosphate transmembrane transporter activity** | | 21 | 4.15E-05 | -1.46 |
| GO:0005436 |  | *sodium:phosphate symporter activity* | 14 | 5.97E-03 | -1.22 |
| GO:0015098 |  | *molybdate ion transmembrane transporter activity* | 7 | 2.29E-02 | -1.40 |
| GO:0003696 | **satellite DNA binding** | | 2 | 1.13E-02 | -2.51 |
| GO:0004065 | **arylsulfatase activity** | | 25 | 1.58E-03 | -1.05 |
| GO:0003943 |  | *N-acetylgalactosamine-4-sulfatase activity* | 22 | 5.33E-03 | -0.99 |
| GO:0042281 | **dolichyl pyrophosphate Man9GlcNAc2 alpha-1,3-glucosyltransferase activity** | | 4 | 1.94E-03 | -2.26 |
| GO:0052658 | **inositol-1,4,5-trisphosphate 5-phosphatase activity** | | 9 | 4.32E-03 | -1.54 |
| GO:0052826 |  | *inositol hexakisphosphate 2-phosphatase activity* | 4 | 3.49E-02 | -1.67 |
| GO:0052833 |  | *inositol monophosphate 4-phosphatase activity* | 9 | 1.77E-02 | -1.30 |
| GO:0052832 |  | *inositol monophosphate 3-phosphatase activity* | 9 | 1.77E-02 | -1.30 |
| GO:0008934 |  | *inositol monophosphate 1-phosphatase activity* | 9 | 1.77E-02 | -1.30 |
| GO:0052659 |  | *inositol-1,3,4,5-tetrakisphosphate 5-phosphatase activity* | 8 | 6.43E-03 | -1.55 |
| GO:0003777 | **microtubule motor activity** | | 124 | 2.97E-02 | -0.32 |
| GO:0003729 | **mRNA binding** | | 145 | 7.08E-04 | -0.46 |
| GO:0019133 | **choline monooxygenase activity** | | 1 | 9.25E-03 | -3.43 |
| GO:0016614 | **oxidoreductase activity, acting on CH-OH group of donors** | | 52 | 7.21E-04 | -0.78 |
| GO:0008327 | **methyl-CpG binding** | | 17 | 4.16E-03 | -1.15 |
| GO:0047876 | **endoglycosylceramidase activity** | | 4 | 4.68E-03 | -2.11 |
| GO:0016627 | **oxidoreductase activity, acting on the CH-CH group of donors** | | 10 | 4.47E-02 | -1.06 |
| GO:0004439 | **phosphatidylinositol-4,5-bisphosphate 5-phosphatase activity** | | 16 | 1.84E-03 | -1.28 |
| GO:0004438 |  | *phosphatidylinositol-3-phosphatase activity* | 14 | 2.91E-02 | -0.97 |
| GO:0003993 |  | *acid phosphatase activity* | 9 | 4.56E-02 | -1.11 |
| GO:0034595 |  | *phosphatidylinositol phosphate 5-phosphatase activity* | 8 | 2.16E-02 | -1.33 |
| GO:0034596 |  | *phosphatidylinositol phosphate 4-phosphatase activity* | 5 | 3.21E-02 | -1.55 |
| GO:0052629 |  | *phosphatidylinositol-3,5-bisphosphate 3-phosphatase activity* | 7 | 4.10E-02 | -1.27 |
| GO:0008106 | **alcohol dehydrogenase (NADP+) activity** | | 22 | 3.12E-02 | -0.77 |
| GO:0005319 | **lipid transporter activity** | | 23 | 1.62E-02 | -0.84 |
| GO:0003677 | **DNA binding** | | 1462 | 4.44E-25 | -0.45 |
| GO:0042802 | **identical protein binding** | | 384 | 2.97E-07 | -0.43 |
| GO:0046983 |  | *protein dimerization activity* | 35 | 3.76E-02 | -0.58 |
| GO:0046982 |  | *protein heterodimerization activity* | 222 | 6.13E-04 | -0.38 |
| GO:0032403 |  | *protein complex binding* | 99 | 1.14E-04 | -0.64 |
| GO:0008134 |  | *transcription factor binding* | 166 | 3.17E-06 | -0.60 |
| GO:0019899 |  | *enzyme binding* | 175 | 3.37E-05 | -0.52 |
| GO:0019904 |  | *protein domain specific binding* | 98 | 1.37E-03 | -0.53 |
| GO:0008092 |  | *cytoskeletal protein binding* | 46 | 1.25E-02 | -0.61 |
| GO:0031072 |  | *heat shock protein binding* | 23 | 4.60E-02 | -0.69 |
| GO:0051082 |  | *unfolded protein binding* | 34 | 3.91E-02 | -0.59 |
| GO:0005102 |  | *receptor binding* | 143 | 8.43E-03 | -0.36 |
| GO:0042803 |  | *protein homodimerization activity* | 705 | 2.52E-03 | -0.18 |
| GO:0004561 | **alpha-N-acetylglucosaminidase activity** | | 3 | 3.61E-05 | -3.16 |
| GO:0008456 |  | *alpha-N-acetylgalactosaminidase activity* | 4 | 8.88E-03 | -1.99 |
| GO:0004563 |  | *beta-N-acetylhexosaminidase activity* | 17 | 3.63E-02 | -0.85 |
| GO:0033919 | **glucan 1,3-alpha-glucosidase activity** | | 4 | 8.64E-03 | -1.99 |
| GO:0004338 |  | *glucan exo-1,3-beta-glucosidase activity* | 4 | 1.08E-02 | -1.95 |
| GO:0004574 |  | *oligo-1,6-glucosidase activity* | 3 | 4.56E-02 | -1.80 |
| GO:0097110 | **scaffold protein binding** | | 25 | 9.04E-04 | -1.10 |
| GO:0005524 | **ATP binding** | | 2526 | 8.04E-19 | -0.29 |
| GO:0001882 |  | *nucleoside binding* | 4 | 3.31E-02 | -1.69 |
| GO:0032549 |  | *ribonucleoside binding* | 7 | 6.07E-03 | -1.65 |
| GO:0000166 |  | *nucleotide binding* | 361 | 9.05E-17 | -0.73 |
| GO:0047499 | **calcium-independent phospholipase A2 activity** | | 7 | 6.76E-03 | -1.63 |
| GO:0004621 |  | *glycosylphosphatidylinositol phospholipase D activity* | 2 | 1.56E-02 | -2.43 |
| GO:0050290 |  | *sphingomyelin phosphodiesterase D activity* | 2 | 3.02E-02 | -2.24 |
| GO:0004771 |  | *sterol esterase activity* | 6 | 1.13E-02 | -1.65 |
| GO:0004435 |  | *phosphatidylinositol phospholipase C activity* | 25 | 2.44E-02 | -0.75 |
| GO:0047714 |  | *galactolipase activity* | 3 | 3.35E-02 | -1.89 |
| GO:0018738 | **S-formylglutathione hydrolase activity** | | 2 | 4.78E-02 | -2.09 |
| GO:2001065 | **mannan binding** | | 1 | 7.60E-03 | -3.53 |
| GO:0035798 | **2-alkenal reductase (NADP+) activity** | | 3 | 3.29E-02 | -1.90 |
| GO:0017147 | **Wnt-protein binding** | | 16 | 1.93E-02 | -0.98 |
| GO:0003887 | **DNA-directed DNA polymerase activity** | | 34 | 4.87E-03 | -0.81 |
| GO:0016301 |  | *kinase activity* | 72 | 1.19E-03 | -0.63 |
| GO:0003899 |  | *DNA-directed RNA polymerase activity* | 41 | 2.09E-02 | -0.60 |
| GO:0003743 | **translation initiation factor activity** | | 67 | 1.10E-02 | -0.51 |
| GO:0004797 | **thymidine kinase activity** | | 3 | 3.19E-02 | -1.91 |
| GO:0019894 | **kinesin binding** | | 17 | 1.76E-02 | -0.96 |
| GO:0045296 | **cadherin binding** | | 51 | 1.96E-02 | -0.54 |
| GO:0016922 | **ligand-dependent nuclear receptor binding** | | 10 | 4.41E-02 | -1.06 |
| GO:0070905 | **serine binding** | | 5 | 3.26E-02 | -1.54 |
| GO:0002039 | **p53 binding** | | 52 | 1.20E-02 | -0.58 |
| GO:0051219 | **phosphoprotein binding** | | 12 | 9.76E-03 | -1.23 |
| GO:0044325 | **ion channel binding** | | 72 | 3.95E-02 | -0.40 |
| GO:0042162 | **telomeric DNA binding** | | 12 | 3.77E-03 | -1.37 |
| GO:0030165 | **PDZ domain binding** | | 78 | 3.64E-03 | -0.54 |
| GO:0070742 |  | *C2H2 zinc finger domain binding* | 11 | 1.12E-02 | -1.26 |
| GO:0031208 |  | *POZ domain binding* | 79 | 1.58E-02 | -0.45 |
| GO:0070087 |  | *chromo shadow domain binding* | 5 | 1.40E-02 | -1.73 |
| GO:0047485 | **protein N-terminus binding** | | 52 | 3.31E-02 | -0.49 |
| GO:0004583 | **dolichyl-phosphate-glucose-glycolipid alpha-glucosyltransferase activity** | | 1 | 1.23E-02 | -3.29 |
| GO:0008013 | **beta-catenin binding** | | 58 | 1.36E-04 | -0.84 |
| GO:0008384 | **IkappaB kinase activity** | | 3 | 2.25E-02 | -2.00 |
| GO:0008767 | **UDP-galactopyranose mutase activity** | | 4 | 3.66E-02 | -1.66 |
| GO:0016811 | **hydrolase activity, acting on carbon-nitrogen (but not peptide) bonds, in linear amides** | | 5 | 1.82E-02 | -1.68 |
| GO:0032041 |  | *NAD-dependent histone deacetylase activity (H3-K14 specific)* | 13 | 3.26E-02 | -0.99 |
| GO:0043546 | **molybdopterin cofactor binding** | | 23 | 2.22E-03 | -1.06 |
| GO:0046920 | **alpha-(1-&gt** | | 36 | 1.45E-04 | -1.06 |
| GO:0018392 |  | *glycoprotein 3-alpha-L-fucosyltransferase activity* | 16 | 1.10E-04 | -1.56 |
| GO:0004020 | **adenylylsulfate kinase activity** | | 8 | 2.88E-02 | -1.28 |
| GO:0000287 | **magnesium ion binding** | | 226 | 6.09E-05 | -0.44 |
| GO:0008022 | **protein C-terminus binding** | | 109 | 2.39E-02 | -0.35 |
| GO:0050839 | **cell adhesion molecule binding** | | 98 | 3.72E-04 | -0.60 |
| GO:0003756 | **protein disulfide isomerase activity** | | 41 | 3.88E-04 | -0.93 |
| GO:0004653 | **polypeptide N-acetylgalactosaminyltransferase activity** | | 44 | 4.00E-05 | -1.03 |
| GO:0015020 |  | *glucuronosyltransferase activity* | 25 | 3.97E-02 | -0.68 |
| GO:0003830 |  | *beta-1,4-mannosylglycoprotein 4-beta-N-acetylglucosaminyltransferase activity* | 3 | 2.07E-02 | -2.02 |
| GO:0008375 |  | *acetylglucosaminyltransferase activity* | 25 | 4.13E-02 | -0.68 |
| GO:0003980 |  | *UDP-glucose:glycoprotein glucosyltransferase activity* | 3 | 8.08E-03 | -2.24 |
| GO:0004971 | **alpha-amino-3-hydroxy-5-methyl-4-isoxazole propionate selective glutamate receptor activity** | | 5 | 2.85E-03 | -2.02 |
| GO:0005229 |  | *intracellular calcium activated chloride channel activity* | 52 | 2.19E-02 | -0.52 |
| GO:0005231 |  | *excitatory extracellular ligand-gated ion channel activity* | 2 | 3.05E-02 | -2.24 |
| GO:0005227 |  | *calcium activated cation channel activity* | 22 | 1.16E-02 | -0.90 |
| GO:0046915 |  | *transition metal ion transmembrane transporter activity* | 3 | 1.41E-02 | -2.11 |
| GO:0005262 |  | *calcium channel activity* | 100 | 5.82E-03 | -0.45 |
| GO:0005261 |  | *cation channel activity* | 72 | 3.11E-02 | -0.42 |
| GO:0005245 |  | *voltage-gated calcium channel activity* | 49 | 2.46E-02 | -0.53 |
| GO:0004517 | **nitric-oxide synthase activity** | | 23 | 1.17E-02 | -0.88 |
| GO:0001077 | **RNA polymerase II core promoter proximal region sequence-specific DNA binding transcription factor activity involved in positive regulation of transcription** | | 132 | 5.27E-03 | -0.40 |
| GO:0001618 | **virus receptor activity** | | 41 | 2.84E-03 | -0.78 |
| GO:0042393 | **histone binding** | | 66 | 2.22E-04 | -0.76 |
| GO:0004566 | **beta-glucuronidase activity** | | 2 | 1.15E-03 | -3.05 |
| GO:0004022 | **alcohol dehydrogenase (NAD) activity** | | 3 | 3.19E-02 | -1.91 |
| GO:0016615 |  | *malate dehydrogenase activity* | 4 | 4.74E-02 | -1.59 |
| GO:0050129 | **N-formylglutamate deformylase activity** | | 3 | 4.00E-02 | -1.84 |
| GO:0016884 | **carbon-nitrogen ligase activity, with glutamine as amido-N-donor** | | 7 | 1.66E-02 | -1.47 |
| GO:0031625 | **ubiquitin protein ligase binding** | | 149 | 2.35E-04 | -0.50 |
| GO:0008536 |  | *Ran GTPase binding* | 16 | 3.95E-04 | -1.44 |
| GO:0019901 |  | *protein kinase binding* | 220 | 3.96E-02 | -0.22 |
| GO:0019903 |  | *protein phosphatase binding* | 19 | 3.66E-03 | -1.11 |
| GO:0017137 |  | *Rab GTPase binding* | 107 | 1.06E-03 | -0.52 |
| GO:0043422 |  | *protein kinase B binding* | 8 | 3.32E-02 | -1.24 |
| GO:0005080 |  | *protein kinase C binding* | 23 | 4.09E-02 | -0.71 |
| GO:0005001 | **transmembrane receptor protein tyrosine phosphatase activity** | | 15 | 3.11E-02 | -0.93 |
| GO:0047676 | **arachidonate-CoA ligase activity** | | 17 | 3.99E-02 | -0.83 |
| GO:0008353 | **RNA polymerase II carboxy-terminal domain kinase activity** | | 16 | 4.62E-03 | -1.18 |
| GO:0030362 | **protein phosphatase type 4 regulator activity** | | 5 | 4.33E-02 | -1.47 |
| GO:0004693 | **cyclin-dependent protein serine/threonine kinase activity** | | 33 | 1.81E-02 | -0.68 |
| GO:0003688 | **DNA replication origin binding** | | 11 | 1.48E-02 | -1.22 |
| GO:0016715 | **oxidoreductase activity, acting on paired donors, with incorporation or reduction of molecular oxygen, reduced ascorbate as one donor, and incorporation of one atom of oxygen** | | 4 | 3.81E-02 | -1.65 |
| GO:0003723 | **RNA binding** | | 479 | 2.73E-15 | -0.60 |
| GO:0004386 | **helicase activity** | | 87 | 1.08E-05 | -0.79 |
| GO:0042626 |  | *ATPase activity, coupled to transmembrane movement of substances* | 122 | 1.97E-02 | -0.34 |
| GO:0008094 |  | *DNA-dependent ATPase activity* | 33 | 3.10E-05 | -1.20 |
| GO:0016887 |  | *ATPase activity* | 249 | 5.97E-06 | -0.47 |
| GO:0004649 | **polyADP-ribose glycohydrolase activity** | | 5 | 4.81E-02 | -1.44 |
| GO:0004806 | **triglyceride lipase activity** | | 29 | 4.86E-02 | -0.61 |
| GO:0019136 | **deoxynucleoside kinase activity** | | 2 | 4.88E-02 | -2.09 |
| GO:0047372 | **acylglycerol lipase activity** | | 22 | 4.92E-02 | -0.70 |
| GO:0102009 | **proline dipeptidase activity** | | 2 | 3.03E-02 | -2.24 |
| GO:0103073 | **anandamide amidohydrolase activity** | | 7 | 1.66E-02 | -1.47 |

| **Supplementary Table 4.** KEGG pathways significantly down-regulated with lowered pH. As in ST2. | | | | |
| --- | --- | --- | --- | --- |
| KEGG Pathway | Pathway Description | # contigs | Adj *p-*value | LOR |
| map:KO01100 | Metabolic pathways | 1997 | 3.94E-08 | -0.17 |
| map:KO04142 | Lysosome | 216 | 1.13E-05 | -0.41 |
| map:KO00980 | Metabolism of xenobiotics by cytochrome P450 | 62 | 9.51E-05 | -0.67 |
| map:KO00982 | Drug metabolism - cytochrome P450 | 48 | 9.61E-05 | -0.75 |
| map:KO00330 | Arginine and proline metabolism | 106 | 1.49E-04 | -0.50 |
| map:KO03040 | Spliceosome | 173 | 1.68E-04 | -0.39 |
| map:KO00512 | Mucin type O-glycan biosynthesis | 75 | 2.23E-04 | -0.58 |
| map:KO00511 | Other glycan degradation | 36 | 4.13E-04 | -0.78 |
| map:KO03460 | Fanconi anemia pathway | 66 | 2.10E-03 | -0.52 |
| map:KO04146 | Peroxisome | 164 | 2.19E-03 | -0.33 |
| map:KO00513 | Various types of N-glycan biosynthesis | 109 | 3.46E-03 | -0.38 |
| map:KO03013 | RNA transport | 175 | 3.50E-03 | -0.30 |
| map:KO00053 | Ascorbate and aldarate metabolism | 31 | 7.32E-03 | -0.65 |
| map:KO00944 | Flavone and flavonol biosynthesis | 2 | 7.80E-03 | -1.85 |
| map:KO00643 | Styrene degradation; xenobiotics biodegradation | 13 | 8.71E-03 | -0.94 |
| map:KO05204 | Chemical carcinogenesis | 79 | 8.88E-03 | -0.40 |

| **Supplementary Table 5.** Biological process GO terms significantly associated with up-regulation in lowered pH, as evaluated by Babelomics’ logistic regression. As in ST2. | | | | | | | | | | |
| --- | --- | --- | --- | --- | --- | --- | --- | --- | --- | --- |
| GO ID | | GO Description | | # contigs | | Adj *p-*value | | LOR | |  |
| GO:0002448 | **mast cell mediated immunity** | | | | 2 | | 3.74E-02 | | 1.63 | |
| GO:0031033 | **myosin filament organization** | | | | 123 | | 1.91E-08 | | 0.70 | |
| GO:0071689 | | | *muscle thin filament assembly* | | 126 | | 1.77E-07 | | 0.65 | |
| GO:0007527 | | | *adult somatic muscle development* | | 128 | | 1.37E-07 | | 0.65 | |
| GO:0055014 | | | *atrial cardiac muscle cell development* | | 6 | | 2.27E-03 | | 1.44 | |
| GO:0055015 | | | *ventricular cardiac muscle cell development* | | 9 | | 2.40E-02 | | 0.97 | |
| GO:0030239 | | | *myofibril assembly* | | 146 | | 5.19E-07 | | 0.58 | |
| GO:0030241 | | | *skeletal muscle myosin thick filament assembly* | | 142 | | 2.62E-08 | | 0.65 | |
| GO:0055003 | | | *cardiac myofibril assembly* | | 15 | | 4.86E-02 | | 0.70 | |
| GO:0045214 | | | *sarcomere organization* | | 204 | | 1.03E-06 | | 0.49 | |
| GO:0042692 | | | *muscle cell differentiation* | | 134 | | 6.65E-07 | | 0.60 | |
| GO:0040011 | **locomotion** | | | | 172 | | 4.84E-06 | | 0.50 | |
| GO:0060361 | **flight** | |  | | 126 | | 2.02E-08 | | 0.69 | |
| GO:0030167 | **proteoglycan catabolic process** | | | | 2 | | 2.80E-03 | | 2.17 | |
| GO:0045820 | | | *negative regulation of glycolytic process* | | 3 | | 1.44E-02 | | 1.58 | |
| GO:0060050 | | | *positive regulation of protein glycosylation* | | 5 | | 1.29E-02 | | 1.32 | |
| GO:0006936 | **muscle contraction** | | | | 212 | | 8.84E-06 | | 0.44 | |
| GO:0050955 | | | *thermoception* | | 1 | | 1.58E-02 | | 2.45 | |
| GO:0006939 | | | *smooth muscle contraction* | | 17 | | 1.19E-02 | | 0.81 | |
| GO:0006937 | | | *regulation of muscle contraction* | | 23 | | 1.50E-02 | | 0.69 | |
| GO:0080154 | **regulation of fertilization** | | | | 20 | | 3.68E-05 | | 1.14 | |
| GO:0019102 | | | *male somatic sex determination* | | 1 | | 1.00E-02 | | 2.63 | |
| GO:0042006 | | | *masculinization of hermaphroditic germ-line* | | 1 | | 1.00E-02 | | 2.63 | |
| GO:0001887 | **selenium compound metabolic process** | | | | 4 | | 1.93E-02 | | 1.37 | |
| GO:0032197 | **transposition, RNA-mediated** | | | | 4 | | 2.38E-02 | | 1.34 | |
| GO:0022406 | **membrane docking** | | | | 5 | | 1.29E-02 | | 1.32 | |
| GO:0051013 | **microtubule severing** | | | | 16 | | 2.33E-02 | | 0.76 | |
| GO:0071140 | **resolution of mitotic recombination intermediates** | | | | 3 | | 3.04E-02 | | 1.44 | |
| GO:0006730 | **one-carbon metabolic process** | | | | 43 | | 4.18E-02 | | 0.45 | |
| GO:1902548 | **negative regulation of cellular response to vascular endothelial growth factor stimulus** | | | | 2 | | 2.80E-03 | | 2.17 | |
| GO:2001113 | | | *negative regulation of cellular response to hepatocyte growth factor stimulus* | | 2 | | 2.80E-03 | | 2.17 | |
| GO:0071409 | | | *cellular response to cycloheximide* | | 4 | | 2.38E-02 | | 1.34 | |
| GO:0071305 | | | *cellular response to vitamin D* | | 2 | | 1.71E-02 | | 1.81 | |
| GO:0071347 | | | *cellular response to interleukin-1* | | 17 | | 1.39E-02 | | 0.80 | |
| GO:0032417 | **positive regulation of sodium:proton antiporter activity** | | | | 5 | | 1.29E-02 | | 1.32 | |
| GO:2001256 | | | *regulation of store-operated calcium entry* | | 1 | | 2.33E-02 | | 2.31 | |
| GO:0010884 | | | *positive regulation of lipid storage* | | 3 | | 4.87E-02 | | 1.34 | |
| GO:0008588 | | | *release of cytoplasmic sequestered NF-kappaB* | | 4 | | 2.42E-02 | | 1.33 | |
| GO:0018277 | **protein deamination** | | | | 2 | | 1.79E-02 | | 1.80 | |
| GO:2000609 | **regulation of thyroid hormone generation** | | | | 1 | | 3.70E-03 | | 3.67 | |
| GO:1904106 | **protein localization to microvillus** | | | | 1 | | 4.14E-02 | | 2.10 | |
| GO:0006557 | **S-adenosylmethioninamine biosynthetic process** | | | | 2 | | 9.11E-03 | | 1.94 | |
| GO:0005993 | **trehalose catabolic process** | | | | 2 | | 1.60E-02 | | 1.82 | |
| GO:0018009 | **N-terminal peptidyl-L-cysteine N-palmitoylation** | | | | 1 | | 4.98E-02 | | 2.04 | |
| GO:0048017 | **inositol lipid-mediated signaling** | | | | 11 | | 2.33E-02 | | 0.90 | |
| GO:0006679 | **glucosylceramide biosynthetic process** | | | | 1 | | 1.25E-02 | | 2.54 | |
| GO:0009609 | **response to symbiotic bacterium** | | | | 6 | | 2.66E-02 | | 1.12 | |
| GO:0006957 | | | *complement activation, alternative pathway* | | 2 | | 4.86E-02 | | 1.57 | |
| GO:0043152 | | | *induction of bacterial agglutination* | | 2 | | 3.74E-02 | | 1.63 | |
| GO:0048858 | **cell projection morphogenesis** | | | | 2 | | 2.53E-02 | | 1.72 | |
| GO:0042744 | **hydrogen peroxide catabolic process** | | | | 72 | | 2.34E-02 | | 0.39 | |
| GO:0032199 | **reverse transcription involved in RNA-mediated transposition** | | | | 4 | | 2.38E-02 | | 1.34 | |
| GO:0050821 | **protein stabilization** | | | | 215 | | 1.27E-02 | | 0.25 | |
| GO:0006048 | **UDP-N-acetylglucosamine biosynthetic process** | | | | 14 | | 3.43E-02 | | 0.76 | |
| GO:0019417 | **sulfur oxidation** | | | | 5 | | 1.34E-02 | | 1.31 | |
| GO:0006528 | **asparagine metabolic process** | | | | 4 | | 3.87E-02 | | 1.25 | |
| GO:0036369 | **transcription factor catabolic process** | | | | 1 | | 1.00E-02 | | 2.63 | |
| GO:0042750 | **hibernation** | | | | 1 | | 7.88E-03 | | 2.73 | |
| GO:0010735 | **positive regulation of transcription via serum response element binding** | | | | 4 | | 1.46E-02 | | 1.42 | |
| GO:0051247 | | | *positive regulation of protein metabolic process* | | 4 | | 2.38E-02 | | 1.34 | |
| GO:1902203 | **negative regulation of hepatocyte growth factor receptor signaling pathway** | | | | 2 | | 2.80E-03 | | 2.17 | |
| GO:0097192 | **extrinsic apoptotic signaling pathway in absence of ligand** | | | | 9 | | 2.55E-02 | | 0.96 | |
| GO:0007225 | **patched ligand maturation** | | | | 1 | | 4.98E-02 | | 2.04 | |
| GO:0044240 | **multicellular organismal lipid catabolic process** | | | | 1 | | 1.99E-02 | | 2.36 | |
| GO:0007427 | **epithelial cell migration, open tracheal system** | | | | 162 | | 3.29E-04 | | 0.41 | |
| GO:0003380 | | | *establishment or maintenance of cytoskeleton polarity involved in gastrulation* | | 2 | | 9.48E-03 | | 1.93 | |
| GO:0007298 | | | *border follicle cell migration* | | 242 | | 1.00E-02 | | 0.25 | |
| GO:0007304 | | | *chorion-containing eggshell formation* | | 18 | | 4.37E-02 | | 0.66 | |
| GO:0007306 | | | *eggshell chorion assembly* | | 27 | | 2.18E-02 | | 0.62 | |
| GO:0007316 | | | *pole plasm RNA localization* | | 5 | | 2.19E-02 | | 1.24 | |
| GO:0009991 | **response to extracellular stimulus** | | | | 9 | | 3.77E-02 | | 0.90 | |
| GO:1900408 | **negative regulation of cellular response to oxidative stress** | | | | 1 | | 5.07E-03 | | 2.98 | |
| GO:0030317 | **sperm motility** | | | | 46 | | 7.35E-03 | | 0.56 | |
| GO:0031424 | **keratinization** | | | | 13 | | 3.08E-04 | | 1.22 | |
| GO:0060537 | | | *muscle tissue development* | | 10 | | 4.46E-02 | | 0.84 | |
| GO:0060562 | | | *epithelial tube morphogenesis* | | 1 | | 4.30E-02 | | 2.09 | |
| GO:0072080 | | | *nephron tubule development* | | 1 | | 1.86E-02 | | 2.39 | |
| GO:2000696 | | | *regulation of epithelial cell differentiation involved in kidney development* | | 1 | | 4.30E-02 | | 2.09 | |
| GO:1901250 | | | *negative regulation of lung goblet cell differentiation* | | 3 | | 4.14E-02 | | 1.38 | |
| GO:0021536 | | | *diencephalon development* | | 4 | | 4.12E-02 | | 1.23 | |
| GO:0032331 | | | *negative regulation of chondrocyte differentiation* | | 8 | | 1.38E-02 | | 1.09 | |
| GO:1901509 | | | *regulation of endothelial tube morphogenesis* | | 3 | | 3.68E-02 | | 1.40 | |
| GO:0060347 | | | *heart trabecula formation* | | 6 | | 2.27E-03 | | 1.44 | |
| GO:0061140 | | | *lung secretory cell differentiation* | | 3 | | 4.14E-02 | | 1.38 | |
| GO:0034968 | **histone lysine methylation** | | | | 61 | | 4.12E-02 | | 0.38 | |
| GO:0018026 | | | *peptidyl-lysine monomethylation* | | 51 | | 4.76E-02 | | 0.40 | |
| GO:0097029 | **mature conventional dendritic cell differentiation** | | | | 2 | | 1.85E-02 | | 1.79 | |
| GO:0050860 | **negative regulation of T cell receptor signaling pathway** | | | | 10 | | 5.01E-03 | | 1.11 | |
| GO:0070885 | | | *negative regulation of calcineurin-NFAT signaling cascade* | | 5 | | 1.29E-02 | | 1.32 | |
| GO:0050868 | | | *negative regulation of T cell activation* | | 6 | | 9.60E-03 | | 1.27 | |
| GO:0060907 | | | *positive regulation of macrophage cytokine production* | | 4 | | 3.20E-02 | | 1.28 | |

| \| **Supplementary Table 6.** Molecular function GO terms significantly associated with down-regulation in lowered pH, as evaluated by Babelomics’ logistic regression. As in ST2. \| \| \| \| \| \| \| --- \| --- \| --- \| --- \| --- \| --- \| \| GO ID \| GO Description \| \| # contigs \| Adj p-value \| LOR \| \| GO:0003774 \| **motor activity** \| \| 234 \| 1.59E-08 \| 0.53 \| \| GO:0003868 \| **4-hydroxyphenylpyruvate dioxygenase activity** \| \| 4 \| 4.40E-02 \| 1.22 \| \| GO:0004014 \| **adenosylmethionine decarboxylase activity** \| \| 2 \| 9.11E-03 \| 1.94 \| \| GO:0004343 \| **glucosamine 6-phosphate N-acetyltransferase activity** \| \| 2 \| 1.34E-02 \| 1.86 \| \| GO:0004555 \| \| alpha,alpha-trehalase activity \| 3 \| 3.70E-02 \| 1.40 \| \| GO:0004613 \| \| phosphoenolpyruvate carboxykinase (GTP) activity \| 1 \| 3.63E-02 \| 2.15 \| \| GO:0004623 \| **phospholipase A2 activity** \| \| 21 \| 5.75E-03 \| 0.81 \| \| GO:0004683 \| **calmodulin-dependent protein kinase activity** \| \| 36 \| 1.89E-04 \| 0.83 \| \| GO:0004691 \| \| cAMP-dependent protein kinase activity \| 19 \| 1.83E-02 \| 0.73 \| \| GO:0004720 \| **protein-lysine 6-oxidase activity** \| \| 3 \| 5.10E-03 \| 1.75 \| \| GO:0004850 \| **uridine phosphorylase activity** \| \| 1 \| 4.21E-02 \| 2.10 \| \| GO:0005122 \| **torso binding** \| \| 2 \| 1.15E-04 \| 2.90 \| \| GO:0005298 \| **proline:sodium symporter activity** \| \| 1 \| 4.22E-02 \| 2.10 \| \| GO:0008010 \| **structural constituent of chitin-based larval cuticle** \| \| 5 \| 6.31E-05 \| 1.90 \| \| GO:0008048 \| **calcium sensitive guanylate cyclase activator activity** \| \| 5 \| 1.68E-03 \| 1.58 \| \| GO:0008120 \| **ceramide glucosyltransferase activity** \| \| 1 \| 1.25E-02 \| 2.54 \| \| GO:0008307 \| **structural constituent of muscle** \| \| 182 \| 2.35E-08 \| 0.58 \| \| GO:0008440 \| **inositol-1,4,5-trisphosphate 3-kinase activity** \| \| 11 \| 4.54E-02 \| 0.81 \| \| GO:0008568 \| **microtubule-severing ATPase activity** \| \| 15 \| 2.83E-03 \| 0.99 \| \| GO:0009036 \| **Type II site-specific deoxyribonuclease activity** \| \| 4 \| 2.38E-02 \| 1.34 \| \| GO:0009931 \| \| Ca^2+^-dependent protein serine/threonine kinase act. \| 17 \| 1.90E-03 \| 0.97 \| \| GO:0015019 \| **heparan-α-glucosaminide N-acetyltransferase activity** \| \| 3 \| 1.90E-02 \| 1.53 \| \| GO:0015227 \| **acyl carnitine transmembrane transporter activity** \| \| 7 \| 3.81E-02 \| 1.00 \| \| GO:0015927 \| **trehalase activity** \| \| 1 \| 2.46E-02 \| 2.29 \| \| GO:0016279 \| **protein-lysine N-methyltransferase activity** \| \| 68 \| 2.75E-02 \| 0.39 \| \| GO:0017016 \| \| Ras GTPase binding \| 21 \| 4.35E-02 \| 0.61 \| \| GO:0019826 \| **oxygen sensor activity** \| \| 8 \| 2.39E-02 \| 1.02 \| \| GO:0030023 \| **extracellular matrix constituent conferring elasticity** \| \| 4 \| 2.73E-04 \| 1.92 \| \| GO:0030898 \| **actin-dependent ATPase activity** \| \| 136 \| 3.30E-08 \| 0.66 \| \| GO:0032184 \| **SUMO polymer binding** \| \| 4 \| 5.22E-03 \| 1.57 \| \| GO:0033549 \| **MAP kinase phosphatase activity** \| \| 6 \| 1.67E-03 \| 1.47 \| \| GO:0042132 \| **fructose 1,6-bisphosphate 1-phosphatase activity** \| \| 6 \| 1.94E-06 \| 2.03 \| \| GO:0042302 \| **structural constituent of cuticle** \| \| 49 \| 2.78E-06 \| 0.88 \| \| GO:0042623 \| \| ATPase activity, coupled \| 145 \| 1.09E-06 \| 0.57 \| \| GO:0043914 \| **NADPH:sulfur oxidoreductase activity** \| \| 5 \| 1.34E-02 \| 1.31 \| \| GO:0050998 \| **nitric-oxide synthase binding** \| \| 1 \| 3.79E-02 \| 2.13 \| \| GO:0070025 \| **carbon monoxide binding** \| \| 8 \| 2.39E-02 \| 1.02 \| \| GO:0070026 \| **nitric oxide binding** \| \| 8 \| 2.39E-02 \| 1.02 \| \| GO:0070492 \| **oligosaccharide binding** \| \| 3 \| 1.22E-02 \| 1.61 \| |
| --- | --- | --- | --- | --- | --- | --- | --- | --- | --- | --- | --- | --- | --- | --- | --- | --- | --- | --- | --- | --- | --- | --- | --- | --- | --- | --- | --- | --- | --- | --- | --- | --- | --- | --- | --- | --- | --- | --- | --- | --- | --- | --- | --- | --- | --- | --- | --- | --- | --- | --- | --- | --- | --- | --- | --- | --- | --- | --- | --- | --- | --- | --- | --- | --- | --- | --- | --- | --- | --- | --- | --- | --- | --- | --- | --- | --- | --- | --- | --- | --- | --- | --- | --- | --- | --- | --- | --- | --- | --- | --- | --- | --- | --- | --- | --- | --- | --- | --- | --- | --- | --- | --- | --- | --- | --- | --- | --- | --- | --- | --- | --- | --- | --- | --- | --- | --- | --- | --- | --- | --- | --- | --- | --- | --- | --- | --- | --- | --- | --- | --- | --- | --- | --- | --- | --- | --- | --- | --- | --- | --- | --- | --- | --- | --- | --- | --- | --- | --- | --- | --- | --- | --- | --- | --- | --- | --- | --- | --- | --- | --- | --- | --- | --- | --- | --- | --- | --- | --- | --- | --- | --- | --- | --- | --- | --- | --- | --- | --- | --- | --- | --- | --- | --- | --- | --- | --- | --- | --- | --- | --- | --- | --- | --- | --- | --- | --- | --- | --- | --- | --- | --- | --- | --- | --- | --- | --- | --- | --- | --- | --- | --- | --- | --- | --- | --- | --- | --- | --- | --- | --- | --- | --- | --- | --- | --- | --- | --- | --- | --- | --- | --- | --- | --- | --- | --- | --- | --- | --- | --- | --- | --- | --- | --- | --- | --- | --- |

| **Supplementary Table 7.** KEGG pathways significantly up-regulated with lowered pH. As in ST2. | | | | |
| --- | --- | --- | --- | --- |
| KEGG Pathway | Pathway Description | # contigs | Adj *p-*value | LOR |
| map:KO04530 | Tight junction | 254 | 2.74E-17 | 0.62 |
| map:KO05416 | Viral myocarditis | 150 | 5.81E-16 | 0.74 |
| map:KO04260 | Cardiac muscle contraction | 198 | 4.88E-13 | 0.61 |
| map:KO04261 | Adrenergic signaling in cardiomyocytes | 306 | 2.57E-12 | 0.49 |
| map:KO05130 | Pathogenic Escherichia coli infection | 70 | 4.21E-06 | 0.64 |
| map:KO03010 | Ribosome | 115 | 7.05E-04 | 0.39 |
| map:KO04921 | Oxytocin signaling pathway | 236 | 2.41E-03 | 0.26 |
| map:KO04744 | Phototransduction | 59 | 3.94E-03 | 0.46 |
| map:KO04152 | AMPK signaling pathway | 117 | 4.21E-03 | 0.34 |
| map:KO01523 | Antifolate resistance | 87 | 5.31E-03 | 0.37 |
| map:KO04626 | Plant-pathogen interaction | 74 | 5.95E-03 | 0.40 |
| map:KO04810 | Regulation of actin cytoskeleton | 185 | 9.70E-03 | 0.25 |
| map:KO04910 | Insulin signaling pathway | 205 | 9.74E-03 | 0.23 |

| **Supplementary Table 8.** Annotation of the 151 contigs that were significantly differentially expressed in *Calanus glacialis* nauplii exposed to a range of pH levels from 8.05 to 7.5. Contig sequences were matched against arthropod sequences in the National Center for Biotechnology Information (NCBI) non-redundant (nr) protein database and the entire Uniprot protein database using Blastx. For nr matches, GeneInfo (GI), GenBank (GB) and RefSeq protein identifiers are provided. Uniprot proteins were annotated with KEGG Gene ID, KEGG pathways, Gene Ontology (GO) terms and and GO keywords. The log2 fold change and p-value measure the slope and significance of the differential expression of contigs with pH, from DESeq2. Log2 fold change is the log2 of the relative change in the contig's expression with a reduction in one unit of pH. All differentially expressed contigs are provided, though only 64 had significant matches in nr and 39 in Uniprot. Three rows of annotation only are shown; the complete ST8 table and entire transcriptome is available in the online Supporting Information. | | | | | | | | | | | | |
| --- | --- | --- | --- | --- | --- | --- | --- | --- | --- | --- | --- | --- |
| **Contig Name** | **Contig Length (bp)** | **Top nr Match** | **Uniprot** | **Uniprot Description** | **KEGG GeneID** | **KEGG Pathway** | **GO terms: Biological process** | **GO terms: Molecular function** | **GO terms: Cellular component** | **Keywords** | **Log2 Fold Change** | ***p*- value** |
| contig# 52597 | 284 | gi\|586962512\|gb\|AHK05644.1\| ATP-binding cassette transporter sub-family C member 1 isoform X5 [Tigriopus japonicus] | O35379 | Multidrug resistance-associated protein 1 | mmu:17250 | mmu05206, mmu02010, mmu04977, mmu04071 | ATP binding(GO:0005524);ATPase activity(GO:0016887);ATPase activity, coupled to transmembrane movement of substances(GO:0042626); |  | basolateral plasma membrane(GO:0016323);extracellular exosome(GO:0070062);integral component of plasma membrane(GO:0005887);membrane(GO:0016020);plasma membrane(GO:0005886); | ATP-binding;Cell membrane;Complete proteome;Glycoprotein;Hydrolase;Membrane;Nucleotide-binding;Phosphoprotein;REFerence proteome;Repeat;Transmembrane;Transmembrane helix;Transport.; | -3.84 | 1.39E-08 |
| contig# 37085 | 537 |  |  |  |  |  |  |  |  |  | -3.59 | 6.07E-08 |
| contig# 20908 | 1031 | gi\|357624231\|gb\|EHJ75089.1\| molting fluid carboxypeptidase A [Danaus plexippus] | P04069 | Carboxypeptidase B |  |  | metallocarboxypeptidase activity(GO:0004181);zinc ion binding(GO:0008270); |  | extracellular region(GO:0005576); | Carboxypeptidase;Direct protein sequencing;Hydrolase;Metal-binding;Metalloprotease;Protease;Secreted;Zinc.; | -3.34 | 5.03E-07 |
| contig# 45384 | 270 |  |  |  |  |  |  |  |  |  | -3.27 | 4.02E-08 |
| contig# 29424 | 939 |  |  |  |  |  |  |  |  |  | -3.19 | 2.95E-06 |
| contig# 45102 | 206 |  |  |  |  |  |  |  |  |  | -3.17 | 2.43E-06 |
| contig# 4342 | 2700 | gi\|357608164\|gb\|EHJ65855.1\| Endoprotease FURIN [Danaus plexippus] | P30432 | Furin-like protease 2 | dme:Dmel_CG18734 | | serine-type endopeptidase activity(GO:0004252); | negative regulation of secretion(GO:0051048);protein processing(GO:0016485);proteolysis(GO:0006508);regulation of glucose metabolic process(GO:0010906); | integral component of membrane(GO:0016021);plasma membrane(GO:0005886); | Alternative splicing;Cleavage on pair of basic residues;Complete proteome;Disulfide bond;Glycoprotein;Hydrolase;Membrane;Protease;REFerence proteome;Repeat;Serine protease;Signal;Transmembrane;Transmembrane helix;Zymogen.; | -3.16 | 3.65E-06 |
| contig# 52412 | 203 |  |  |  |  |  |  |  |  |  | -3.15 | 4.14E-06 |
| contig# 23350 | 1049 |  |  |  |  |  |  |  |  |  | -3.11 | 8.55E-07 |
| contig# 49862 | 730 | gi\|1022774799\|gb\|KZS18781.1\| Uncharacterized protein APZ42_015340 [Daphnia magna] |  |  |  |  |  |  |  |  | -3.07 | 4.77E-06 |
| contig# 32563 | 233 | gi\|924564277\|gb\|ALC48902.1\| CG3108 [Drosophila busckii] | P04069 | Carboxypeptidase B |  |  | metallocarboxypeptidase activity(GO:0004181);zinc ion binding(GO:0008270); |  | extracellular region(GO:0005576); | Carboxypeptidase;Direct protein sequencing;Hydrolase;Metal-binding;Metalloprotease;Protease;Secreted;Zinc.; | -3.06 | 6.27E-06 |
| contig# 18663 | 1047 | gi\|225712486\|gb\|ACO12089.1\| Cuticle protein 19 [Lepeophtheirus salmonis] | P26967 | Adult-specific cuticular protein ACP-20 |  |  | structural constituent of cuticle(GO:0042302); |  |  | Cuticle;Direct protein sequencing;Repeat;Signal.; | -3.04 | 5.88E-06 |
| contig# 25825 | 406 |  |  |  |  |  |  |  |  |  | -3.02 | 7.47E-06 |
| contig# 24446 | 271 |  |  |  |  |  |  |  |  |  | -3.00 | 2.26E-06 |
| contig# 12244 | 4822 | gi\|1022763132\|gb\|KZS08792.1\| Uncharacterized protein APZ42_027471 [Daphnia magna] |  |  |  |  |  |  |  |  | -2.97 | 1.33E-05 |
| contig# 1996 | 591 |  |  |  |  |  |  |  |  |  | -2.95 | 1.51E-05 |
| contig# 26903 | 843 |  |  |  |  |  |  |  |  |  | -2.93 | 1.84E-05 |
| contig# 51461 | 435 |  |  |  |  |  |  |  |  |  | -2.92 | 1.44E-05 |
| contig# 28354 | 377 |  |  |  |  |  |  |  |  |  | -2.92 | 4.64E-07 |
| contig# 36253 | 302 | gi\|1022772025\|gb\|KZS16226.1\| C-type lectin CTL - galactose binding [Daphnia magna] |  |  |  |  |  |  |  |  | -2.90 | 9.11E-06 |
| contig# 12500 | 2129 | gi\|195160393\|ref\|XP_002021060.1\| GL25033 [Drosophila persimilis] >gi\|194118173\|gb\|EDW40216.1\| GL25033 [Drosophila persimilis] | Q7M3M6 | Ecdysone-induced protein 74EF |  |  | sequence-specific DNA binding(GO:0043565);transcription factor activity, sequence-specific DNA binding(GO:0003700); | multicellular organism development(GO:0007275);transcription, DNA-templated(GO:0006351); | nucleus(GO:0005634); | Coiled coil;Developmental protein;DNA-binding;Nucleus;Transcription;Transcription regulation.; | -2.89 | 1.14E-05 |
| contig# 5533 | 826 |  |  |  |  |  |  |  |  |  | -2.86 | 2.62E-05 |
| contig# 6429 | 534 |  |  |  |  |  |  |  |  |  | -2.84 | 1.74E-05 |
| contig# 15466 | 523 | gi\|409184026\|gb\|AFV27512.1\| cuticular protein R&R 1 [Apis cerana cerana] >gi\|409184028\|gb\|AFV27513.1\| cuticular protein R&R 1 [Apis cerana cerana] | P82120 | Cuticle protein 7 |  |  | structural constituent of cuticle(GO:0042302); |  |  | Cuticle;Direct protein sequencing;Repeat.; | -2.82 | 6.53E-06 |
| contig# 40451 | 335 | gi\|155966212\|gb\|ABU41060.1\| hypothetical protein [Lepeophtheirus salmonis] |  |  |  |  |  |  |  |  | -2.82 | 3.46E-05 |
| contig# 39238 | 1374 | gi\|1022771967\|gb\|KZS16182.1\| Uncharacterized protein APZ42_018109 [Daphnia magna] |  |  |  |  |  |  |  |  | -2.82 | 1.95E-05 |
| contig# 21923 | 1582 |  |  |  |  |  |  |  |  |  | -2.81 | 1.52E-05 |
| contig# 16018 | 968 |  |  |  |  |  |  |  |  |  | -2.81 | 3.56E-05 |
| contig# 52288 | 338 |  |  |  |  |  |  |  |  |  | -2.80 | 3.45E-05 |
| contig# 38526 | 759 |  |  |  |  |  |  |  |  |  | -2.80 | 3.41E-05 |
| contig# 8499 | 528 |  |  |  |  |  |  |  |  |  | -2.79 | 3.68E-05 |
| contig# 3739 | 1976 | gi\|1022770611\|gb\|KZS15011.1\| Dual oxidase maturation factor 2 [Daphnia magna] | Q1HG43 | Dual oxidase maturation factor 1 | hsa:90527 |  |  | hydrogen peroxide metabolic process(GO:0042743);positive regulation of neuron differentiation(GO:0045666);positive regulation of reactive oxygen species metabolic process(GO:2000379);protein transport(GO:0015031);regulation of inflammatory response(GO:0050727);regulation of thyroid hormone generation(GO:2000609); | endoplasmic reticulum membrane(GO:0005789);integral component of membrane(GO:0016021);membrane(GO:0016020);plasma membrane(GO:0005886); | Alternative splicing;Complete proteome;Glycoprotein;Membrane;Polymorphism;Protein transport;REFerence proteome;Transmembrane;Transmembrane helix;Transport.; | -2.78 | 1.13E-05 |
| contig# 9563 | 266 | gi\|299119867\|gb\|ADJ11736.1\| GA19326, partial [Drosophila miranda] >gi\|299119869\|gb\|ADJ11737.1\| GA19326, partial [Drosophila miranda] >gi\|299119871\|gb\|ADJ11738.1\| GA19326, partial [Drosophila miranda] >gi\|299119873\|gb\|ADJ11739.1\| GA19326, partial [Drosophila miranda] >gi\|299119875\|gb\|ADJ11740.1\| GA19326, partial [Drosophila miranda] >gi\|299119877\|gb\|ADJ11741.1\| GA19326, partial [Drosophila miranda] >gi\|299119879\|gb\|ADJ11742.1\| GA19326, partial [Drosophila miranda] >gi\|299119881\|gb\|ADJ11743.1\| GA19326, partial [Drosophila miranda] >gi\|299119883\|gb\|ADJ11744.1\| GA19326, partial [Drosophila miranda] >gi\|299119885\|gb\|ADJ11745.1\| GA19326, partial [Drosophila miranda] >gi\|299119887\|gb\|ADJ11746.1\| GA19326, partial [Drosophila miranda] >gi\|299119889\|gb\|ADJ11747.1\| GA19326, partial [Drosophila miranda] >gi\|299119891\|gb\|ADJ11748.1\| GA19326, partial [Drosophila miranda] >gi\|299119893\|gb\|ADJ11749.1\| GA19326, partial [Drosophila miranda] >gi\|299119895\|gb\|ADJ11750.1\| GA19326, partial [Drosophila miranda] >gi\|299119897\|gb\|ADJ11751.1\| GA19326, partial [Drosophila pseudoobscura] >gi\|299119899\|gb\|ADJ11752.1\| GA19326, partial [Drosophila pseudoobscura] >gi\|299119901\|gb\|ADJ11753.1\| GA19326, partial [Drosophila pseudoobscura] >gi\|299119903\|gb\|ADJ11754.1\| GA19326, partial [Drosophila pseudoobscura] >gi\|299119905\|gb\|ADJ11755.1\| GA19326, partial [Drosophila pseudoobscura] >gi\|299119907\|gb\|ADJ11756.1\| GA19326, partial [Drosophila pseudoobscura] >gi\|299119909\|gb\|ADJ11757.1\| GA19326, partial [Drosophila pseudoobscura] >gi\|299119911\|gb\|ADJ11758.1\| GA19326, partial [Drosophila pseudoobscura] >gi\|299119913\|gb\|ADJ11759.1\| GA19326, partial [Drosophila pseudoobscura] >gi\|299119915\|gb\|ADJ11760.1\| GA19326, partial [Drosophila pseudoobscura] >gi\|299119917\|gb\|ADJ11761.1\| GA19326, partial [Drosophila pseudoobscura] >gi\|299119919\|gb\|ADJ11762.1\| GA19326, partial [Drosophila pseudoobscura] >gi\|299119921\|gb\|ADJ11763.1\| GA19326, partial [Drosophila pseudoobscura] >gi\|299119923\|gb\|ADJ11764.1\| GA19326, partial [Drosophila pseudoobscura] >gi\|299119925\|gb\|ADJ11765.1\| GA19326, partial [Drosophila pseudoobscura] >gi\|299119927\|gb\|ADJ11766.1\| GA19326, partial [Drosophila pseudoobscura] |  |  |  |  |  |  |  |  | -2.78 | 3.76E-05 |
| contig# 30197 | 582 |  |  |  |  |  |  |  |  |  | -2.78 | 4.77E-05 |
| contig# 10636 | 1296 |  |  |  |  |  |  |  |  |  | -2.77 | 7.79E-06 |
| contig# 32662 | 230 |  |  |  |  |  |  |  |  |  | -2.77 | 5.13E-05 |
| contig# 35515 | 225 |  |  |  |  |  |  |  |  |  | -2.77 | 4.59E-05 |
| contig# 42475 | 509 |  |  |  |  |  |  |  |  |  | -2.77 | 3.77E-05 |
| contig# 8392 | 207 |  |  |  |  |  |  |  |  |  | -2.76 | 4.28E-05 |
| contig# 34735 | 295 |  |  |  |  |  |  |  |  |  | -2.76 | 5.44E-05 |
| contig# 5376 | 3212 | gi\|925684195\|gb\|KOX80717.1\| Serine/threonine-protein kinase Doa, partial [Melipona quadrifasciata] | P49762 | Serine/threonine-protein kinase Doa | dme:Dmel_CG42320 | | ATP binding(GO:0005524);protein kinase activity(GO:0004672);protein serine/threonine kinase activity(GO:0004674);protein serine/threonine/tyrosine kinase activity(GO:0004712);protein tyrosine kinase activity(GO:0004713); | autophagic cell death(GO:0048102);blastoderm segmentation(GO:0007350);brain morphogenesis(GO:0048854);central nervous system development(GO:0007417);chaeta morphogenesis(GO:0008407);chitin-based embryonic cuticle biosynthetic process(GO:0008362);compound eye development(GO:0048749);compound eye photoreceptor development(GO:0042051);karyosome formation(GO:0030717);locomotion involved in locomotory behavior(GO:0031987);negative regulation of male germ cell proliferation(GO:2000255);negative regulation of MyD88-dependent toll-like receptor signaling pathway(GO:0034125);nervous system development(GO:0007399);oogenesis(GO:0048477);photoreceptor cell maintenance(GO:0045494);protein autophosphorylation(GO:0046777);protein phosphorylation(GO:0006468);protein secretion(GO:0009306);regulation of alternative mRNA splicing, via spliceosome(GO:0000381);salivary gland cell autophagic cell death(GO:0035071);sex differentiation(GO:0007548);startle response(GO:0001964);visual perception(GO:0007601); | cytoplasm(GO:0005737);cytosol(GO:0005829);endoplasmic reticulum(GO:0005783);microtubule associated complex(GO:0005875);nucleus(GO:0005634); | Alternative splicing;ATP-binding;Complete proteome;Cytoplasm;Developmental protein;Kinase;Nucleotide-binding;Nucleus;Phosphoprotein;REFerence proteome;Sensory transduction;Serine/threonine-protein kinase;Transferase;Tyrosine-protein kinase;Vision.; | -2.76 | 1.15E-05 |
| contig# 30561 | 207 |  |  |  |  |  |  |  |  |  | -2.74 | 6.06E-05 |
| contig# 34734 | 243 |  |  |  |  |  |  |  |  |  | -2.74 | 5.93E-05 |
| contig# 36256 | 1726 | gi\|170035735\|ref\|XP_001845723.1\| coagulation factor XI [Culex quinquefasciatus] >gi\|167878029\|gb\|EDS41412.1\| coagulation factor XI [Culex quinquefasciatus] | P00765 | Trypsin-1 |  |  | metal ion binding(GO:0046872);serine-type endopeptidase activity(GO:0004252); | digestion(GO:0007586); | extracellular space(GO:0005615); | Calcium;Digestion;Direct protein sequencing;Disulfide bond;Hydrolase;Metal-binding;Protease;Secreted;Serine protease.; | -2.71 | 3.13E-05 |
| contig# 26623 | 369 | gi\|646704158\|gb\|KDR12496.1\| Urokinase-type plasminogen activator [Zootermopsis nevadensis] | P26262 | Plasma kallikrein | mmu:16621 | mmu04610 | serine-type endopeptidase activity(GO:0004252); | blood coagulation(GO:0007596);fibrinolysis(GO:0042730);inflammatory response(GO:0006954);liver regeneration(GO:0097421);plasminogen activation(GO:0031639);positive regulation of fibrinolysis(GO:0051919); | extracellular exosome(GO:0070062);extracellular space(GO:0005615); | Blood coagulation;Complete proteome;Direct protein sequencing;Disulfide bond;Fibrinolysis;Glycoprotein;Hemostasis;Hydrolase;Inflammatory response;Protease;REFerence proteome;Repeat;Secreted;Serine protease;Signal;Zymogen.; | -2.71 | 3.05E-05 |
| contig# 23653 | 220 |  |  |  |  |  |  |  |  |  | -2.70 | 6.27E-05 |
| contig# 19501 | 347 | gi\|290462717\|gb\|ADD24406.1\| Cuticle protein 7 [Lepeophtheirus salmonis] >gi\|290562577\|gb\|ADD38684.1\| Cuticle protein 7 [Lepeophtheirus salmonis] | P26967 | Adult-specific cuticular protein ACP-20 |  |  | structural constituent of cuticle(GO:0042302); |  |  | Cuticle;Direct protein sequencing;Repeat;Signal.; | -2.67 | 5.75E-05 |
| contig# 27619 | 556 | gi\|646722401\|gb\|KDR23414.1\| DmX-like protein 2 [Zootermopsis nevadensis] | Q8TDJ6 | DmX-like protein 2 | hsa:23312 |  | Rab GTPase binding(GO:0017137); |  | cell junction(GO:0030054);extracellular space(GO:0005615);synaptic vesicle(GO:0008021);synaptic vesicle membrane(GO:0030672); | Alternative splicing;Cell junction;Coiled coil;Complete proteome;Cytoplasmic vesicle;Diabetes mellitus;Direct protein sequencing;Disease mutation;Hypogonadotropic hypogonadism;Membrane;Neuropathy;Phosphoprotein;Polymorphism;REFerence proteome;Repeat;Synapse;WD repeat.; | -2.65 | 8.05E-05 |
| contig# 50069 | 359 | gi\|914098223\|ref\|NP_001298282.1\| uncharacterized LOC106111232 precursor [Papilio polytes] >gi\|389610659\|dbj\|BAM18941.1\| C-type lectin - galactose binding [Papilio polytes] |  |  |  |  |  |  |  |  | -2.65 | 7.59E-05 |
| contig# 22714 | 248 | gi\|155966212\|gb\|ABU41060.1\| hypothetical protein [Lepeophtheirus salmonis] |  |  |  |  |  |  |  |  | -2.64 | 1.13E-04 |
| contig# 37947 | 396 | gi\|155966212\|gb\|ABU41060.1\| hypothetical protein [Lepeophtheirus salmonis] |  |  |  |  |  |  |  |  | -2.64 | 1.16E-04 |
| contig# 11856 | 750 | gi\|1022777459\|gb\|KZS21013.1\| UNC93 protein [Daphnia magna] | Q9Y115 | UNC93-like protein | dme:Dmel_CG4928 | |  |  | integral component of membrane(GO:0016021);plasma membrane(GO:0005886); | Complete proteome;Glycoprotein;Membrane;REFerence proteome;Transmembrane;Transmembrane helix.; | -2.64 | 7.01E-05 |
| contig# 970 | 717 | gi\|155966139\|gb\|ABU41024.1\| hypothetical protein [Lepeophtheirus salmonis] |  |  |  |  |  |  |  |  | -2.63 | 1.09E-04 |
| contig# 23101 | 266 |  |  |  |  |  |  |  |  |  | -2.62 | 1.67E-05 |
| contig# 19305 | 391 | gi\|155966163\|gb\|ABU41036.1\| hypothetical protein [Lepeophtheirus salmonis] |  |  |  |  |  |  |  |  | -2.61 | 1.26E-04 |
| contig# 46312 | 487 |  |  |  |  |  |  |  |  |  | -2.61 | 1.24E-04 |
| contig# 42946 | 248 | gi\|170035729\|ref\|XP_001845720.1\| trypsin [Culex quinquefasciatus] >gi\|167878026\|gb\|EDS41409.1\| trypsin [Culex quinquefasciatus] | Q61096 | Myeloblastin | mmu:19152 |  | enzyme binding(GO:0019899);serine-type endopeptidase activity(GO:0004252); | collagen catabolic process(GO:0030574);mature conventional dendritic cell differentiation(GO:0097029);negative regulation of phagocytosis(GO:0050765);phagocytosis(GO:0006909); | cytosol(GO:0005829);extracellular exosome(GO:0070062);extracellular space(GO:0005615);plasma membrane(GO:0005886); | Collagen degradation;Complete proteome;Disulfide bond;Glycoprotein;Hydrolase;Protease;REFerence proteome;Serine protease;Signal;Zymogen.; | -2.59 | 5.01E-05 |
| contig# 11186 | 461 | gi\|225712486\|gb\|ACO12089.1\| Cuticle protein 19 [Lepeophtheirus salmonis] |  |  |  |  |  |  |  |  | -2.59 | 1.09E-04 |
| contig# 8889 | 416 |  |  |  |  |  |  |  |  |  | -2.57 | 1.68E-04 |
| contig# 23665 | 513 |  |  |  |  |  |  |  |  |  | -2.57 | 1.05E-04 |
| contig# 52156 | 383 |  |  |  |  |  |  |  |  |  | -2.57 | 1.63E-04 |
| contig# 42035 | 307 |  |  |  |  |  |  |  |  |  | -2.56 | 1.60E-04 |
| contig# 37993 | 778 | gi\|572263449\|ref\|XP_006609934.1\| PREDICTED: uncharacterized protein LOC102672049 isoform X1 [Apis dorsata] |  |  |  |  |  |  |  |  | -2.56 | 7.46E-05 |
| contig# 25926 | 1076 | gi\|288869500\|ref\|NP_001165857.1\| cuticular protein analogous to peritrophins 3-B precursor [Acyrthosiphon pisum] |  |  |  |  |  |  |  |  | -2.55 | 4.04E-05 |
| contig# 31070 | 453 |  |  |  |  |  |  |  |  |  | -2.55 | 5.64E-05 |
| contig# 13532 | 516 |  |  |  |  |  |  |  |  |  | -2.53 | 1.60E-04 |
| contig# 27406 | 239 | gi\|675368420\|gb\|KFM61322.1\| Protein lethal(2)essential for life, partial [Stegodyphus mimosarum] | Q3T149 | Heat shock protein beta-1 |  |  |  |  | cytoplasm(GO:0005737);nucleus(GO:0005634);spindle(GO:0005819); | Acetylation;Chaperone;Complete proteome;Cytoplasm;Cytoskeleton;Nucleus;Phosphoprotein;REFerence proteome;Stress response.; | -2.53 | 2.11E-04 |
| contig# 18310 | 594 | gi\|675390144\|gb\|KFM83041.1\| Superoxide dismutase [Cu-Zn], partial [Stegodyphus mimosarum] | P34461 | Extracellular superoxide dismutase [Cu-Zn] |  |  | copper ion binding(GO:0005507);superoxide dismutase activity(GO:0004784);zinc ion binding(GO:0008270); | superoxide metabolic process(GO:0006801); | cytoplasm(GO:0005737);extracellular space(GO:0005615);membrane(GO:0016020); | Alternative splicing;Antioxidant;Complete proteome;Copper;Disulfide bond;Glycoprotein;Membrane;Metal-binding;Oxidoreductase;REFerence proteome;Secreted;Signal;Zinc.; | -2.51 | 2.32E-04 |
| contig# 34937 | 981 | gi\|943456533\|gb\|ALL42061.1\| transcription factor SOX-4-like protein [Antheraea pernyi] | P40656 | Putative transcription factor SOX-14 | dme:Dmel_CG3090 | | DNA binding, bending(GO:0008301);sequence-specific DNA binding(GO:0043565); | dendrite morphogenesis(GO:0048813);metamorphosis(GO:0007552);neuron remodeling(GO:0016322);regulation of neuron remodeling(GO:1904799);regulation of transcription from RNA polymerase II promoter(GO:0006357);transcription, DNA-templated(GO:0006351); | nucleus(GO:0005634); | Complete proteome;DNA-binding;Nucleus;REFerence proteome;Transcription;Transcription regulation.; | -2.51 | 2.32E-04 |
| contig# 20334 | 465 | gi\|1022772025\|gb\|KZS16226.1\| C-type lectin CTL - galactose binding [Daphnia magna] |  |  |  |  |  |  |  |  | -2.51 | 4.44E-05 |
| contig# 1375 | 4791 | gi\|675389799\|gb\|KFM82696.1\| Myosin light chain kinase, smooth muscle, partial [Stegodyphus mimosarum] | Q28824 | Myosin light chain kinase, smooth muscle | bta:338037 | bta04971, bta04611, bta04022, bta04921, bta04270, bta04810, bta04510, bta04020 | ATP binding(GO:0005524);metal ion binding(GO:0046872);myosin light chain kinase activity(GO:0004687); | positive regulation of cell migration(GO:0030335);smooth muscle contraction(GO:0006939); | cleavage furrow(GO:0032154);cytoplasm(GO:0005737);cytoskeleton(GO:0005856);lamellipodium(GO:0030027); | Actin-binding;Alternative initiation;ATP-binding;Calcium;Calmodulin-binding;Cell projection;Complete proteome;Cytoplasm;Cytoskeleton;Disulfide bond;Immunoglobulin domain;Kinase;Magnesium;Metal-binding;Nucleotide-binding;Phosphoprotein;REFerence proteome;Repeat;Serine/threonine-protein kinase;Transferase.; | -2.51 | 2.45E-04 |
| contig# 46377 | 262 |  |  |  |  |  |  |  |  |  | -2.51 | 6.81E-05 |
| contig# 23208 | 851 |  |  |  |  |  |  |  |  |  | -2.50 | 1.90E-04 |
| contig# 17187 | 952 | gi\|1022763722\|gb\|KZS09273.1\| Aquaporin-4 [Daphnia magna] |  |  |  |  |  |  |  |  | -2.48 | 2.90E-04 |
| contig# 42921 | 244 |  |  |  |  |  |  |  |  |  | -2.47 | 2.92E-04 |
| contig# 15362 | 955 | gi\|155966163\|gb\|ABU41036.1\| hypothetical protein [Lepeophtheirus salmonis] |  |  |  |  |  |  |  |  | -2.47 | 2.90E-04 |
| contig# 3070 | 1244 |  |  |  |  |  |  |  |  |  | -2.46 | 1.52E-04 |
| contig# 17528 | 544 |  |  |  |  |  |  |  |  |  | -2.46 | 1.91E-04 |
| contig# 45273 | 231 |  |  |  |  |  |  |  |  |  | -2.46 | 2.66E-04 |
| contig# 25898 | 443 | gi\|155966358\|gb\|ABU41131.1\| hypothetical protein [Lepeophtheirus salmonis] |  |  |  |  |  |  |  |  | -2.46 | 2.97E-04 |
| contig# 40599 | 492 |  |  |  |  |  |  |  |  |  | -2.45 | 2.82E-04 |
| contig# 39520 | 420 | gi\|225712486\|gb\|ACO12089.1\| Cuticle protein 19 [Lepeophtheirus salmonis] | P84252 | Cuticle protein 16.8 |  |  | chitin binding(GO:0008061);structural constituent of cuticle(GO:0042302); |  |  | Chitin-binding;Cuticle;Direct protein sequencing;Pyrrolidone carboxylic acid.; | -2.45 | 2.81E-04 |
| contig# 6116 | 829 | gi\|155966163\|gb\|ABU41036.1\| hypothetical protein [Lepeophtheirus salmonis] |  |  |  |  |  |  |  |  | -2.44 | 3.21E-04 |
| contig# 20448 | 998 |  |  |  |  |  |  |  |  |  | -2.44 | 3.58E-04 |
| contig# 6437 | 250 |  |  |  |  |  |  |  |  |  | -2.44 | 2.81E-04 |
| contig# 26045 | 901 |  |  |  |  |  |  |  |  |  | -2.44 | 3.42E-04 |
| contig# 33487 | 467 |  |  |  |  |  |  |  |  |  | -2.44 | 3.11E-04 |
| contig# 33833 | 419 |  |  |  |  |  |  |  |  |  | -2.43 | 2.53E-04 |
| contig# 31593 | 562 |  |  |  |  |  |  |  |  |  | -2.43 | 1.99E-04 |
| contig# 34817 | 498 | gi\|134085585\|gb\|ABO52851.1\| IP18036p [Drosophila melanogaster] | Q9MZ19 | Potassium voltage-gated channel subfamily B member 1 {ECO:0000250\|UniProtKB:Q14721} | ocu:100008779 | | delayed rectifier potassium channel activity(GO:0005251);protein heterodimerization activity(GO:0046982); | action potential(GO:0001508);cellular response to glucose stimulus(GO:0071333);cellular response to nutrient levels(GO:0031669);glucose homeostasis(GO:0042593);glutamate receptor signaling pathway(GO:0007215);negative regulation of insulin secretion(GO:0046676);positive regulation of calcium ion-dependent exocytosis(GO:0045956);positive regulation of catecholamine secretion(GO:0033605);positive regulation of long term synaptic depression(GO:1900454);positive regulation of norepinephrine secretion(GO:0010701);positive regulation of protein targeting to membrane(GO:0090314);potassium ion transmembrane transport(GO:0071805);potassium ion transport(GO:0006813);protein homooligomerization(GO:0051260);protein targeting to plasma membrane(GO:0072661);regulation of action potential(GO:0098900);regulation of motor neuron apoptotic process(GO:2000671);vesicle docking involved in exocytosis(GO:0006904); | axon(GO:0030424);cell junction(GO:0030054);dendrite(GO:0030425);intracellular(GO:0005622);lateral plasma membrane(GO:0016328);neuronal cell body membrane(GO:0032809);perikaryon(GO:0043204);plasma membrane(GO:0005886);postsynaptic membrane(GO:0045211);sarcolemma(GO:0042383);voltage-gated potassium channel complex(GO:0008076); | Cell junction;Cell membrane;Cell projection;Complete proteome;Exocytosis;Ion channel;Ion transport;Isopeptide bond;Membrane;Phosphoprotein;Postsynaptic cell membrane;Potassium;Potassium channel;Potassium transport;REFerence proteome;Synapse;Synaptosome;Transmembrane;Transmembrane helix;Transport;Ubl conjugation;Voltage-gated channel.; | -2.43 | 2.31E-04 |
| contig# 15075 | 1761 |  |  |  |  |  |  |  |  |  | -2.42 | 1.65E-04 |
| contig# 33318 | 330 |  |  |  |  |  |  |  |  |  | -2.42 | 3.92E-04 |
| contig# 12602 | 5390 | gi\|646707193\|gb\|KDR14054.1\| Protein ELYS [Zootermopsis nevadensis] | Q5U249 | Protein ELYS | xla:397707 |  | DNA binding(GO:0003677); | mRNA transport(GO:0051028);protein transport(GO:0015031); | nuclear pore(GO:0005643); | DNA-binding;mRNA transport;Nuclear pore complex;Nucleus;Protein transport;Translocation;Transport.; | -2.42 | 3.01E-04 |
| contig# 46981 | 297 |  |  |  |  |  |  |  |  |  | -2.42 | 3.89E-04 |
| contig# 39249 | 201 |  |  |  |  |  |  |  |  |  | -2.42 | 4.01E-04 |
| contig# 23006 | 211 |  |  |  |  |  |  |  |  |  | -2.42 | 2.03E-04 |
| contig# 24428 | 270 | gi\|155966358\|gb\|ABU41131.1\| hypothetical protein [Lepeophtheirus salmonis] |  |  |  |  |  |  |  |  | -2.42 | 4.05E-04 |
| contig# 45283 | 383 |  |  |  |  |  |  |  |  |  | -2.41 | 1.84E-04 |
| contig# 48033 | 467 |  |  |  |  |  |  |  |  |  | -2.41 | 2.74E-04 |
| contig# 12886 | 609 |  |  |  |  |  |  |  |  |  | -2.40 | 3.88E-04 |
| contig# 15493 | 274 | gi\|391332048\|ref\|XP_003740450.1\| PREDICTED: uncharacterized protein LOC100899826 [Metaseiulus occidentalis] | P59171 | Acidic phospholipase A2 5 |  |  | calcium ion binding(GO:0005509);phospholipase A2 activity(GO:0004623); | lipid catabolic process(GO:0016042); | extracellular region(GO:0005576); | Calcium;Disulfide bond;Hydrolase;Lipid degradation;Lipid metabolism;Metal-binding;Secreted;Signal.; | -2.40 | 4.63E-04 |
| contig# 43397 | 779 |  |  |  |  |  |  |  |  |  | -2.39 | 1.85E-04 |
| contig# 40830 | 668 | gi\|195167958\|ref\|XP_002024799.1\| GL17911 [Drosophila persimilis] >gi\|194108229\|gb\|EDW30272.1\| GL17911 [Drosophila persimilis] | Q0VC71 | Probable tubulin polyglutamylase TTLL1 | bta:539530 |  | ATP binding(GO:0005524);ligase activity(GO:0016874); | axoneme assembly(GO:0035082);epithelial cilium movement(GO:0003351);protein polyglutamylation(GO:0018095); | cytoplasm(GO:0005737);microtubule(GO:0005874); | ATP-binding;Complete proteome;Cytoplasm;Cytoskeleton;Ligase;Microtubule;Nucleotide-binding;REFerence proteome.; | -2.38 | 1.36E-04 |
| contig# 17917 | 318 |  |  |  |  |  |  |  |  |  | -2.38 | 3.31E-05 |
| contig# 13971 | 1107 |  |  |  |  |  |  |  |  |  | -2.38 | 1.77E-04 |
| contig# 18830 | 353 | gi\|225711840\|gb\|ACO11766.1\| E-selectin precursor [Lepeophtheirus salmonis] | D8VNS6 | C-type lectin |  |  | carbohydrate binding(GO:0030246);metal ion binding(GO:0046872); |  | extracellular region(GO:0005576); | Calcium;Disulfide bond;Lectin;Metal-binding;Secreted;Signal;Toxin.; | -2.38 | 4.75E-04 |
| contig# 50835 | 226 |  |  |  |  |  |  |  |  |  | -2.37 | 4.40E-04 |
| contig# 38755 | 509 | gi\|332018514\|gb\|EGI59104.1\| Zinc finger protein 474 [Acromyrmex echinatior] | Q6V5K9 | Zinc finger protein 474 | mmu:66758 |  | metal ion binding(GO:0046872); |  |  | Complete proteome;Metal-binding;REFerence proteome;Zinc;Zinc-finger.; | -2.36 | 2.64E-04 |
| contig# 931 | 1011 |  |  |  |  |  |  |  |  |  | -2.36 | 1.26E-04 |
| contig# 25931 | 229 |  |  |  |  |  |  |  |  |  | -2.36 | 3.48E-04 |
| contig# 20164 | 549 |  |  |  |  |  |  |  |  |  | -2.35 | 1.75E-04 |
| contig# 20237 | 1216 |  |  |  |  |  |  |  |  |  | -2.34 | 3.90E-04 |
| contig# 22124 | 1930 | gi\|50293089\|gb\|AAT72921.1\| gastric caeca sugar transporter [Locusta migratoria] | Q8MKK4 | Facilitated trehalose transporter Tret1-2 homolog {ECO:0000303\|PubMed:20035867} | dme:Dmel_CG8234 | | glucose transmembrane transporter activity(GO:0005355);sugar(GO:0005351); | glucose import(GO:0046323);hexose transmembrane transport(GO:0035428); | integral component of plasma membrane(GO:0005887);membrane(GO:0016020);plasma membrane(GO:0005886); | Cell membrane;Complete proteome;Glycoprotein;Membrane;REFerence proteome;Transmembrane;Transmembrane helix.; | -2.33 | 4.55E-04 |
| contig# 12239 | 4205 | gi\|194877783\|ref\|XP_001973941.1\| uncharacterized protein Dere_GG21465, isoform A [Drosophila erecta] >gi\|190657128\|gb\|EDV54341.1\| uncharacterized protein Dere_GG21465, isoform A [Drosophila erecta] | Q6ZMW2 | Zinc finger protein 782 | hsa:158431 |  | DNA binding(GO:0003677);metal ion binding(GO:0046872);transcription factor activity, sequence-specific DNA binding(GO:0003700); | regulation of transcription, DNA-templated(GO:0006355);transcription, DNA-templated(GO:0006351); | nucleus(GO:0005634); | Complete proteome;DNA-binding;Metal-binding;Nucleus;Polymorphism;REFerence proteome;Repeat;Transcription;Transcription regulation;Zinc;Zinc-finger.; | -2.32 | 2.70E-04 |
| contig# 5850 | 1602 | gi\|1026546295\|gb\|OAD52285.1\| Clavesin-1 [Eufriesea mexicana] | A6JUQ6 | Clavesin-2 {ECO:0000303\|PubMed:19651769} |  |  | phosphatidylinositol-3,5-bisphosphate binding(GO:0080025);transporter activity(GO:0005215); | lysosome organization(GO:0007040); | clathrin-coated vesicle(GO:0030136);early endosome membrane(GO:0031901);endosome(GO:0005768);trans-Golgi network(GO:0005802); | Complete proteome;Cytoplasmic vesicle;Endosome;Golgi apparatus;Lipid-binding;Membrane;Phosphoprotein;REFerence proteome.; | -2.32 | 3.94E-04 |
| contig# 31064 | 426 |  |  |  |  |  |  |  |  |  | -2.32 | 9.42E-05 |
| contig# 23327 | 337 |  |  |  |  |  |  |  |  |  | -2.30 | 1.81E-04 |
| contig# 34114 | 764 | gi\|496214269\|gb\|AGL41863.1\| GA11431, partial [Drosophila pseudoobscura] | A2AGA4 | Rhomboid-related protein 2 | mmu:230726 | | serine-type endopeptidase activity(GO:0004252); | protein processing(GO:0016485); | integral component of membrane(GO:0016021);mitochondrial inner membrane(GO:0005743);plasma membrane(GO:0005886); | Cell membrane;Complete proteome;Hydrolase;Membrane;Protease;REFerence proteome;Serine protease;Transmembrane;Transmembrane helix.; | -2.29 | 4.27E-04 |
| contig# 20574 | 934 |  |  |  |  |  |  |  |  |  | -2.28 | 4.70E-04 |
| contig# 16976 | 981 |  |  |  |  |  |  |  |  |  | -2.28 | 3.05E-04 |
| contig# 38782 | 475 |  |  |  |  |  |  |  |  |  | -2.27 | 4.60E-04 |
| contig# 4711 | 291 |  |  |  |  |  |  |  |  |  | -2.26 | 2.83E-04 |
| contig# 8339 | 1073 | gi\|952541886\|gb\|KRT86426.1\| C-type lectin, partial [Oryctes borbonicus] |  |  |  |  |  |  |  |  | -2.26 | 4.50E-04 |
| contig# 45781 | 242 |  |  |  |  |  |  |  |  |  | -2.20 | 4.17E-04 |
| contig# 7243 | 6415 | gi\|307181425\|gb\|EFN69020.1\| FH2 domain-containing protein 1 [Camponotus floridanus] | Q9C0D6 | FH2 domain-containing protein 1 | hsa:85462 |  |  |  |  | Complete proteome;Phosphoprotein;Polymorphism;REFerence proteome.; | -2.19 | 4.29E-04 |
| contig# 46142 | 491 | gi\|328899290\|gb\|AEB54634.1\| receptor accessory protein 5 [Procambarus clarkii] | Q29RM3 | Receptor expression-enhancing protein 5 | bta:617543 |  |  |  | integral component of membrane(GO:0016021); | Complete proteome;Membrane;REFerence proteome;Transmembrane;Transmembrane helix.; | -2.16 | 4.20E-04 |
| contig# 13099 | 471 |  |  |  |  |  |  |  |  |  | -2.15 | 4.68E-04 |
| contig# 46525 | 720 |  |  |  |  |  |  |  |  |  | -2.10 | 3.30E-04 |
| contig# 11046 | 596 |  |  |  |  |  |  |  |  |  | -2.09 | 2.74E-04 |
| contig# 9989 | 1139 |  |  |  |  |  |  |  |  |  | -2.04 | 1.54E-04 |
| contig# 9743 | 203 |  |  |  |  |  |  |  |  |  | -1.94 | 4.64E-04 |
| contig# 8199 | 459 |  |  |  |  |  |  |  |  |  | -1.90 | 2.63E-04 |
| contig# 43979 | 1223 | gi\|657600344\|gb\|AID52833.1\| nuclear receptor [Tigriopus japonicus] | P33244 | Nuclear hormone receptor FTZ-F1 | dme:Dmel_CG4059 | | chromatin binding(GO:0003682);DNA binding(GO:0003677);protein heterodimerization activity(GO:0046982);RNA polymerase II transcription factor activity, ligand-activated sequence-specific DNA binding(GO:0004879);steroid hormone receptor activity(GO:0003707);transcription cofactor activity(GO:0003712);transcription factor binding(GO:0008134);transcription regulatory region sequence-specific DNA binding(GO:0000976);transcriptional activator activity, RNA polymerase II core promoter proximal region sequence-specific binding(GO:0001077);zinc ion binding(GO:0008270); | cell death(GO:0008219);dendrite morphogenesis(GO:0048813);imaginal disc-derived leg morphogenesis(GO:0007480);instar larval or pupal development(GO:0002165);juvenile hormone mediated signaling pathway(GO:0035626);lipid homeostasis(GO:0055088);metamorphosis(GO:0007552);mushroom body development(GO:0016319);neuron remodeling(GO:0016322);olfactory behavior(GO:0042048);periodic partitioning(GO:0007365);positive regulation of transcription from RNA polymerase II promoter(GO:0045944);pupariation(GO:0035073);pupation(GO:0035074);regulation of development, heterochronic(GO:0040034);regulation of glucose metabolic process(GO:0010906);regulation of transcription from RNA polymerase II promoter(GO:0006357);regulation of transcription, DNA-templated(GO:0006355);response to ecdysone(GO:0035075);response to hormone(GO:0009725);salivary gland cell autophagic cell death(GO:0035071); | cytoplasm(GO:0005737);nucleus(GO:0005634);RNA polymerase II transcription factor complex(GO:0090575); | 3D-structure;Activator;Alternative splicing;Complete proteome;Direct protein sequencing;DNA-binding;Metal-binding;Nucleus;Receptor;REFerence proteome;Transcription;Transcription regulation;Zinc;Zinc-finger.; | -1.72 | 2.36E-04 |
| contig# 24085 | 799 |  |  |  |  |  |  |  |  |  | -1.72 | 3.62E-04 |
| contig# 489 | 2524 | gi\|657600386\|gb\|AID52854.1\| hormone receptor 38 [Tigriopus japonicus] | P49869 | Probable nuclear hormone receptor HR38 | dme:Dmel_CG1864 | | RNA polymerase II regulatory region sequence-specific DNA binding(GO:0000977);RNA polymerase II transcription factor activity, ligand-activated sequence-specific DNA binding(GO:0004879);steroid hormone receptor activity(GO:0003707);zinc ion binding(GO:0008270); | cuticle development(GO:0042335);epidermis development(GO:0008544);phagocytosis(GO:0006909);positive regulation of transcription from RNA polymerase II promoter(GO:0045944);regulation of adult chitin-containing cuticle pigmentation(GO:0048082);regulation of glucose metabolic process(GO:0010906);transcription, DNA-templated(GO:0006351); | nucleus(GO:0005634); | 3D-structure;Alternative splicing;Complete proteome;Developmental protein;DNA-binding;Metal-binding;Nucleus;Receptor;REFerence proteome;Transcription;Transcription regulation;Zinc;Zinc-finger.; | -1.68 | 5.46E-06 |
| contig# 12325 | 1013 |  |  |  |  |  |  |  |  |  | -1.64 | 4.44E-04 |
| contig# 11128 | 5671 | gi\|1022764186\|gb\|KZS09667.1\| Uncharacterized protein APZ42_026052 [Daphnia magna] |  |  |  |  |  |  |  |  | -1.50 | 5.11E-07 |
| contig# 2280 | 3711 | gi\|646708142\|gb\|KDR14571.1\| Protein ovo [Zootermopsis nevadensis] | P51521 | Protein ovo | dme:Dmel_CG6824 | | DNA binding(GO:0003677);metal ion binding(GO:0046872);RNA polymerase II transcription factor activity, sequence-specific DNA binding(GO:0000981);sequence-specific DNA binding(GO:0043565); | adult feeding behavior(GO:0008343);cuticle development(GO:0042335);cuticle pattern formation(GO:0035017);cuticle pigmentation(GO:0048067);cytoskeleton organization(GO:0007010);epidermal cell differentiation(GO:0009913);female germ-line sex determination(GO:0019099);germ-line sex determination(GO:0018992);imaginal disc-derived leg joint morphogenesis(GO:0016348);negative regulation of transcription from RNA polymerase II promoter(GO:0000122);negative regulation of transcription, DNA-templated(GO:0045892);non-sensory hair organization(GO:0035316);oogenesis(GO:0048477);pheromone metabolic process(GO:0042810);positive regulation of transcription from RNA polymerase II promoter(GO:0045944);positive regulation of transcription, DNA-templated(GO:0045893);positive regulation of transposon integration(GO:0070896);regulation of cell shape(GO:0008360);regulation of transcription, DNA-templated(GO:0006355);transcription from RNA polymerase II promoter(GO:0006366); | cytoplasm(GO:0005737);nucleus(GO:0005634); | Alternative splicing;Complete proteome;Cytoplasm;Developmental protein;Differentiation;DNA-binding;Metal-binding;Nucleus;Oogenesis;REFerence proteome;Repeat;Transcription;Transcription regulation;Zinc;Zinc-finger.; | -1.41 | 1.36E-04 |
| contig# 5768 | 1341 | gi\|675379677\|gb\|KFM72579.1\| TSC22 domain family protein 1, partial [Stegodyphus mimosarum] | Q22544 | Uncharacterized protein T18D3.7 |  |  | transcription factor activity, sequence-specific DNA binding(GO:0003700); |  |  | Complete proteome;REFerence proteome.; | -1.07 | 6.58E-05 |
| contig# 16046 | 1891 | gi\|646713566\|gb\|KDR17870.1\| Serine/threonine-protein kinase Pim-3 [Zootermopsis nevadensis] | O70444 | Serine/threonine-protein kinase pim-3 | rno:64534 |  | ATP binding(GO:0005524);protein serine/threonine kinase activity(GO:0004674); | apoptotic process(GO:0006915);cell cycle(GO:0007049);histone phosphorylation(GO:0016572);negative regulation of apoptotic process(GO:0043066);negative regulation of insulin secretion involved in cellular response to glucose stimulus(GO:0061179);protein autophosphorylation(GO:0046777);protein phosphorylation(GO:0006468);regulation of mitotic cell cycle(GO:0007346); | cytoplasm(GO:0005737); | Apoptosis;ATP-binding;Cell cycle;Complete proteome;Cytoplasm;Kinase;Nucleotide-binding;Phosphoprotein;Proto-oncogene;REFerence proteome;Serine/threonine-protein kinase;Transferase;Ubl conjugation.; | -0.69 | 4.73E-04 |
| contig# 7251 | 934 | gi\|1764109\|gb\|AAC64661.1\| pacifastin light chain precursor [Pacifastacus leniusculus] |  |  |  |  |  |  |  |  | 1.15 | 1.73E-05 |
| contig# 15439 | 2593 |  |  |  |  |  |  |  |  |  | 1.33 | 3.62E-05 |
| contig# 7818 | 2745 | gi\|158289940\|ref\|XP_311553.4\| AGAP010394-PA [Anopheles gambiae str. PEST] >gi\|157018400\|gb\|EAA07199.5\| AGAP010394-PA [Anopheles gambiae str. PEST] | Q0EEE2 | Patched domain-containing protein 3 | mmu:74675 |  |  |  | integral component of membrane(GO:0016021);sperm midpiece(GO:0097225); | Alternative splicing;Complete proteome;Glycoprotein;Membrane;REFerence proteome;Transmembrane;Transmembrane helix.; | 1.38 | 2.88E-04 |
| contig# 6842 | 322 |  | P64918 | Uncharacterized protein Mb2023 | mbo:Mb2023 | | oxidoreductase activity(GO:0016491); |  |  | Complete proteome.; | 1.75 | 4.50E-04 |
| contig# 34102 | 945 | gi\|332031120\|gb\|EGI70697.1\| Integral membrane protein, partial [Acromyrmex echinatior] |  |  |  |  |  |  |  |  | 1.85 | 3.10E-04 |
| contig# 8474 | 251 |  |  |  |  |  |  |  |  |  | 1.88 | 1.01E-04 |
| contig# 3743 | 277 |  |  |  |  |  |  |  |  |  | 2.03 | 1.47E-04 |
| contig# 42282 | 325 | gi\|1022759724\|gb\|KZS06043.1\| Uncharacterized protein APZ42_030532 [Daphnia magna] |  |  |  |  |  |  |  |  | 2.20 | 3.45E-04 |
| contig# 32408 | 267 |  |  |  |  |  |  |  |  |  | 2.44 | 3.48E-04 |
| contig# 40540 | 231 |  |  |  |  |  |  |  |  |  | 2.45 | 1.72E-04 |
| contig# 20554 | 975 | gi\|1022765763\|gb\|KZS10896.1\| Uncharacterized protein APZ42_024564 [Daphnia magna] |  |  |  |  |  |  |  |  | 2.58 | 1.47E-04 |

**Tables and Figures**

| **Supplementary Table 9**. Mean (±SE) pH, temperature (°C), nutrients, and carbonate chemistry in experimental tanks, averaged over the experiment by tank and by treatment. Total scale pH measured spectrophotometrically (pH_T, spec_) and calculated from CT and A_T_ using CO2SYS (pH_T, calc_) are presented. After Table 1 in Bailey et al. (2016). | | | | | | | | | | | | | | | |
| --- | --- | --- | --- | --- | --- | --- | --- | --- | --- | --- | --- | --- | --- | --- | --- |
| pCO_2_  Treatment | Tank | pH_T, spec_ | Temp (°C) | pH_T, spec_ | pH_T, calc_ | pCO_2_ (μatm) | Salinity | A_T_ (μmol kg^-1^) | HCO_3_ (μmol kg^-1^) | CO_3_ (μmol kg^-1^) | CO_2_ (μmol kg^-1^) | Nitrite (μmol L^-1^) | Nitrate (μmol L^-1^) | Phos-phate (μmol L^-1^) | Silicate (μmol L^-1^) |
| **Low** | A | 8.07 ±0.01 | 2.09 ±0.06 | 8.05 ±0.01 | 8.12 ±0.02 | 322  ±15 | 34.2 ±0.1 | 2324 ±2 | 2022 ±11 | 118.9 ±4.5 | 18.7 ±0.9 | 0.08 ±0.01 | 7.09 ±1.55 | 1.98 ±0.53 | 6.63 ±0.39 |
|  | B | 8.04 ±0.02 | 2.07 ±0.05 |  |  |  |  |  |  |  |  |  |  |  |  |
|  | C | 8.04 ±0.02 | 2.04 ±0.05 |  |  |  |  |  |  |  |  |  |  |  |  |
| **Ambient** | A | 7.86 ±0.01 | 1.9 ±0.06 | 7.86 ±0.01 | 7.93 ±0.02 | 530  ±27 | 34.2 ±0.1 | 2324 ±1 | 2122 ±8 | 79.3 ±3.0 | 30.9 ±1.5 | 0.06 ±0.01 | 9.23 ±0.19 | 1.63 ±0.21 | 6.18 ±0.22 |
|  | B | 7.86 ±0.02 | 1.94 ±0.07 |  |  |  |  |  |  |  |  |  |  |  |  |
|  | C | 7.85 ±0.02 | 1.88 ±0.07 |  |  |  |  |  |  |  |  |  |  |  |  |
| **Mid** | A | 7.7 ±0.01 | 1.87 ±0.06 | 7.69 ±0.00 | 7.77 ±0.05 | 808  ±76 | 34.2 ±0.1 | 2325 ±1 | 2172 ±21 | 60.1 ±8.5 | 47.2 ±4.4 | 0.07 ±0.01 | 7.35 ±1.21 | 1.4 ±0.21 | 6.05 ±0.15 |
|  | B | 7.69 ±0.01 | 1.99 ±0.07 |  |  |  |  |  |  |  |  |  |  |  |  |
|  | C | 7.68 ±0.01 | 2.06 ±0.06 |  |  |  |  |  |  |  |  |  |  |  |  |
| **High** | A | 7.47 ±0.01 | 1.69 ±0.07 | 7.47 ±0.00 | 7.44 ±0.01 | 1698 ±37 | 34.2 ±0.1 | 2324 ±2 | 2253 ±2 | 27.4 ±0.5 | 99.3 ±2.0 | 0.06 ±0.01 | 7.33 ±1.22 | 1.53 ±0.31 | 6.14 ±0.19 |
|  | B | 7.47 ±0.00 | 1.85 ±0.08 |  |  |  |  |  |  |  |  |  |  |  |  |
|  | C | 7.47 ±0.01 | 1.79 ±0.07 |  |  |  |  |  |  |  |  |  |  |  |  |
